# Single Cell RNA-Seq Analysis of Human Red Cells

**DOI:** 10.3389/fphys.2022.828700

**Published:** 2022-04-20

**Authors:** Vaibhav Jain, Wen-Hsuan Yang, Jianli Wu, John D. Roback, Simon G. Gregory, Jen-Tsan Chi

**Affiliations:** ^1^ Department of Neurology, Durham, NC, United States; ^2^ Duke Molecular Physiology Institute, Durham, NC, United States; ^3^ Department of Molecular Genetics and Microbiology, Durham, NC, United States; ^4^ Center for Genomic and Computational Biology, Duke University School of Medicine, Durham, NC, United States; ^5^ Center for Transfusion and Cellular Therapies, Durham, NC, United States; ^6^ Department of Pathology and Laboratory Medicine, Emory University School of Medicine, Atlanta, GA, United States

**Keywords:** fetal hemoglobin, ACVR2B, HEMGN, NIX, Red cell, single cell RNA-Seq Storage, RNA

## Abstract

Human red blood cells (RBCs), or erythrocytes, are the most abundant blood cells responsible for gas exchange. RBC diseases affect hundreds of millions of people and impose enormous financial and personal burdens. One well-recognized, but poorly understood feature of RBC populations within the same individual are their phenotypic heterogeneity. The granular characterization of phenotypic RBC variation in normative and disease states may allow us to identify the genetic determinants of red cell diseases and reveal novel therapeutic approaches for their treatment. Previously, we discovered diverse RNA transcripts in RBCs that has allowed us to dissect the phenotypic heterogeneity and malaria resistance of sickle red cells. However, these analyses failed to capture the heterogeneity found in RBC sub-populations. To overcome this limitation, we have performed single cell RNA-Seq to analyze the transcriptional heterogeneity of RBCs from three adult healthy donors which have been stored in the blood bank conditions and assayed at day 1 and day 15. The expression pattern clearly separated RBCs into seven distinct clusters that include one RBC cluster that expresses HBG2 and a small population of RBCs that express fetal hemoglobin (HbF) that we annotated as F cells. Almost all HBG2-expessing cells also express HBB, suggesting bi-allelic expression in single RBC from the HBG2/HBB loci, and we annotated another cluster as reticulocytes based on canonical gene expression. Additional RBC clusters were also annotated based on the enriched expression of *NIX*, *ACVR2B* and *HEMGN*, previously shown to be involved in erythropoiesis. Finally, we found the storage of RBC was associated with an increase in the ACVR2B and F-cell clusters. Collectively, these data indicate the power of single RBC RNA-Seq to capture and discover known and unexpected heterogeneity of RBC population.

## Introduction

Human red blood cell (RBC or erythrocytes) disorders, including various anemia diseases and malaria, affect hundreds of millions of people across the world and exert a huge economic and human burden. Although the basis for many red blood cell disorders is well studied, there are still significant unknowns associated with their complex phenotypes and underlying disease heterogeneity. For example, the first identified “monogenetic molecular disease”, sickle cell disease (SCD) (Pauling et al., 1949), is still poorly understood in terms the heterogeneity associated with the severity of anemia and subsequent complications. Therefore, SCD is often referred as a monogenic disease with polygenetic manifestations (Driss et al., 2009). To understand SCD heterogeneity, previous efforts used GWAS and genomic sequencing to identify the genetic variants associated with the phenotypic heterogeneity of SCD (Piel et al., 2017). However, these associated alleles do not fully explain the enormous SCD heterogeneity.

Red blood cells expressing sickle hemoglobin are known to polymerize and cause sickling, which underlies the pathogenic mechanisms associated with SCD. Even different SCD patients that share the same sickle point mutation in HbS (Sickle Hemoglobin) present with heterogeneous clinical manifestation. One potential source of this heterogeneity may result from the phenotypic variations among RBC population within the same individual. Different RBCs vary significantly in their structure (Picas et al., 2013), density, adhesion, deformity and their response to various treatments (Wang et al., 2013; Alapan et al., 2014). However, RBC heterogeneity may also impact variation in hemolysis, vascular blockage, and clinical manifestation. For example, “dense cells” represent a small population of RBCs but have much higher cellular density often postulated to block microcirculation (Rodgers et al., 1985). Additionally, another RBC population, “F cells,” express fetal hemoglobin and have the ability to inhibit HbS polymerization. Many therapeutic approaches for SCD aim to increase HbF expression and the percentage of F cells. While we know such phenotypic heterogeneity among SCD patients exists we do not have effective methods to accurately characterize RBC heterogeneity, which in turn prevents the functional association of SCD risk alleles with the disease or the development of novel therapeutics.

RBC is routinely used for blood transfusion with more than 100 million units of blood collected worldwide annually (Organization 2014). Standard blood bank protocols allow for the storage of RBCs up to 42 days prior to transfusion. There are conflicting reports whether the RBC quality decreases proportionally as storage time increases, and whether poor clinical outcomes are associated with in the recipients of stored RBC (Tinmouth et al., 2006; Kim Shapiro et al., 2011; Berezina et al., 2002; D'Alessandro et al., 2015). These storage-related issues may be associated with various molecular and biochemical alterations during storage. These storage-associated changes, often collectively referred to as the “storage lesion” (Wolfe 1985), include morphological alteration, biochemical alternations (reduced ATP, 2,3-diphosphoglycerate, and glutathione), decreased oxygen delivery capacity, acidosis, altered cation homeostasis, phosphatidylserine exposure, and oxidative damage (Kim‐Shapiro et al., 2011; Berezina et al., 2002; D'Alessandro et al., 2015; D'Alessandro et al., 2010; Bennett-Guerrero et al., 2007). In addition, the storage of red cell is associated with changes in optic density, which can be used to separate fresh and stored RBC (Park et al., 2019; Park et al., 2021). Interestingly, some of these storage-associated changes are also observed during the physiological RBC aging (Antonelou et al., 2010), raising the possibility that *ex vivo* storage can be a model to investigate RBC aging. Regardless, little is known about the transcriptomic characteristics that drive the phenotypic differences of RBCs.

Given that mature human RBCs lose their nuclei during differentiation, the conventional belief has been that erythrocytes do not contain any nucleic acids. Contrary to this traditional belief, we discovered that human erythrocytes contain abundant and diverse species of microRNAs and other mRNA transcripts, a finding that has been validated by others (Rathjen et al., 2006; Chen et al., 2008; Xue et al., 2008; Sangokoya et al., 2010a; Kannan and Atreya 2010; Azzouzi et al., 2015; Sarachana et al., 2015). Our analysis of erythrocyte microRNAs identified a subgroup of HbSS with more severe anemia due to the repression of NRF2 (Nuclear factor erythroid 2-related factor 2) by the elevated expression of miR-144 (Sangokoya et al., 2010b). This study also revealed the therapeutic potential of NRF2 activation in sickle cell diseases (Doss et al., 2016; Belcher et al., 2017). Besides microRNAs, recent evidence indicates that mature RBCs have a significant number of large RNA species, including mRNA and non-coding RNA (Doss et al., 2015). Furthermore, we and other investigators have found that the RBC storage is associated with significant changes in the RBC transcriptome (Yang et al., 2018). However, these analyses used bulk analysis of RNA that inherently masks the known heterogeneous sub-populations of RBCs. New single cell analysis is needed to overcome the limitations of the previous RBC population studies. Recent advances in transcriptome technology allows us to capture a large number of individual cells to perform various genetic analysis and reveal RBC heterogeneity that has previously been masked in the bulk-cell analysis. For example, our own single cell analyses of the malaria parasites have revealed the male- and female-specific gametocytes (Walzer et al., 2018) and latent heterogeneity of asexual stage of malaria parasites (Walzer et al., 2019). However, the application of these single cell technology has yet to be applied to the discovery and interpretation of RBC heterogeneity.

In this study, we performed single red cell RNA-Seq of circulating RBC from three healthy donors that have been stored for one or 15 days. We found that the expression pattern of single RBCs clearly separates into distinct cellular clusters, including a cluster of reticulocytes with higher levels of RNA expression and a cluster of F-cells. Additional cell clusters are characterized by the expression of *ACVR2B*, *HEMGN* and *NIX*, Interestingly, the storage of RBC was associated an increase in the F-cell and *ACVR2B* cluster. We also employed these scRNA-Seq data to examine the allelic expression of different hemoglobin loci. Distinct subsets of RBCs express HBG2, together with HBA1, HBA2 and HBB. Almost all the HBG2-expressing F cells also express HBB, suggesting a bi-allelic expression of the HBG2/HBB in most F-cells. These results reveal novel insights into the gene expression regulation and heterogeneity among individual RBCs.

## Materials and Methods

### Blood Collection and RNA Purification

All studies were approved by the Institutional Review Board at Emory University. Following informed consent, half unit of the whole blood from healthy adults was drawn into CPD-ADSOL [AS-1] collection set and processed into leukodepleted packed RBC units following standard procedures. Units were stored at 1–6°C in a monitored refrigerator. At day 1 and 15 of storage, 10 ml aliquots were removed via a sterile-docking device fitted with a valve to ensure there was no re-entry of air, or other contamination, into the RBC unit.

### 10X Genomics Drop-Seq for Single Red Blood Cells Sequencing

Three pairs of blood units stored for one and 15 days were submitted for analysis by the 10X Genomics Chromium platform (Zheng et al., 2017). Libraries were prepared following the 10x Genomics Single Cell 3’ Reagent Kits v2 User Guide. Briefly, Single cells were dissociated, then washed and resuspended in a 1x PBS/0.04% BSA solution, at a concentration of 1,000 cells/ul. After size selection (<50um), the cell suspension was washed with a 1x PBS/0.04% BSA solution to remove debris, clumps, dead cells and contaminants, and a Cellometer (Nexcelom) was used to determine the cell viability and concentration to normalize to 1 × 10^6^ cells/ml. We titered each prep to contain ∼10,000 cells per library. Cells were then combined with a master mix that contains reverse transcription reagents. The gel beads carrying the Illumina P7 and R2 primer, a 16bp 10x barcode, a 10bp randomer and a poly-dT primer were loaded onto the chip, together with oil for the emulsion reaction. The Chromium Controller partitions the cells into nanoliter-scale gel beads in emulsion (GEMS) within which reverse-transcription occurs. All cDNAs within a GEM, i.e., from 1 cell, share a common barcode. After the RT reaction, the GEMs were broken and the full length cDNAs cleaned with both Silane Dynabeads and SPRI beads. After purification, the cDNAs were assayed on an Agilent 4200 TapeStation High Sensitivity D5000 ScreenTape for qualitative and quantitative analysis.

The cDNA was enzymatically sheared to a target size of ∼200 bp and then sequencing libraries constructed. This entailed end repair and A-tailing, adapter ligation, SPRI bead clean-up, a sample index PCR, and further SPRI bead clean-ups. The sample index PCR adds a unique sample index for sample multiplexing during sequencing, and Illumina P5 and R1 primer site addition. The final libraries contain P5 and P7 primers used in Illumina bridge amplification. Sequence was generated using paired end sequencing (one end to generate cell specific, barcoded sequence and the other to generate sequence of the expressed poly-A tailed mRNA) on an Illumina sequencing platform (NextSeq 4000).

### Data Analysis

The primary analytical pipeline for the SC analysis followed the recommended protocols from 10X Genomics. Briefly, we processed the raw FASTQ files using Cell Ranger software version 5.0^1^. The first steps of this program demultiplex the raw reads and align the reads to the 10X GRCh38 reference transcriptome and gene expression matrices created for all single cells in each sample. Due to the low RNA content of RBCs being sequenced along with erythrocytes, the “--force-cells” parameter of cellranger count was used to ensure capture of all RBC types.

The secondary statistical analysis was performed using R statistical methodology, R package Seurat^2^, which performs quality control and subsequent analyses on the gene expression matrices produced by CellRanger. In Seurat data was first normalized on the log scale after basic filtering for minimum gene and cell observance frequency cut-offs. We then closely examined the data and performed further filtering based on a range of metrics in attempt to identify and exclude possible multiplets (i.e. instances where more than 1 cell was present and sequenced in a single emulsified gel bead (Setup the Seurat Object 2011). The additional removal of further technical artifacts was performed using regression methods to reduce noise. After quality control procedures were complete, we calculated principal components using the most variably expressed genes in our dataset (Setup the Seurat Object 2011). Significant principal components for downstream analyses were determined through methods mirroring those implemented by Macosko et al. (Macosko et al., 2015), and these principal components were carried forward for two main purposes: to perform cell clustering and to enhance visualization. Cells were grouped into an optimal number of clusters for *de novo* cell type discovery using Seurat’s FindClusters () function (Setup the Seurat Object 2011), graph-based clustering approach with visualization of cells being achieved by the use of UMAP (McInnes et al., 2018), which reduced the information captured in the selected significant principal components to two dimensions, (McInnes et al., 2018). Differential expression of relevant cell marker genes was visualized on UMAP plot to reveal specific individual cell types.

### Cell Trajectory Analysis

The cell trajectory analysis was performed using R package Monocle2 that uses reversed graph embedding to describe multiple fate decisions in a fully unsupervised manner (Qiu et al., 2017). The dimensionality was reduced by performing a Principle Components Analysis (PCA) followed by t-SNE to project cells into two dimensions (Monocle2 Documentation [). Density peak clustering, based on each cell’s local density (Ρ) and the nearest distance (Δ) of a cell to another cell with higher distance, identifies cell clusters in 2-D t-SNE space (Qiu et al., 2017). The top significant genes across all clusters as input for the RGE algorithm were used to define progress through the trajectory (Monocle2 Documentation).

## Results

### Experimental Plans and Protocol Optimization

**FIGURE 1:**
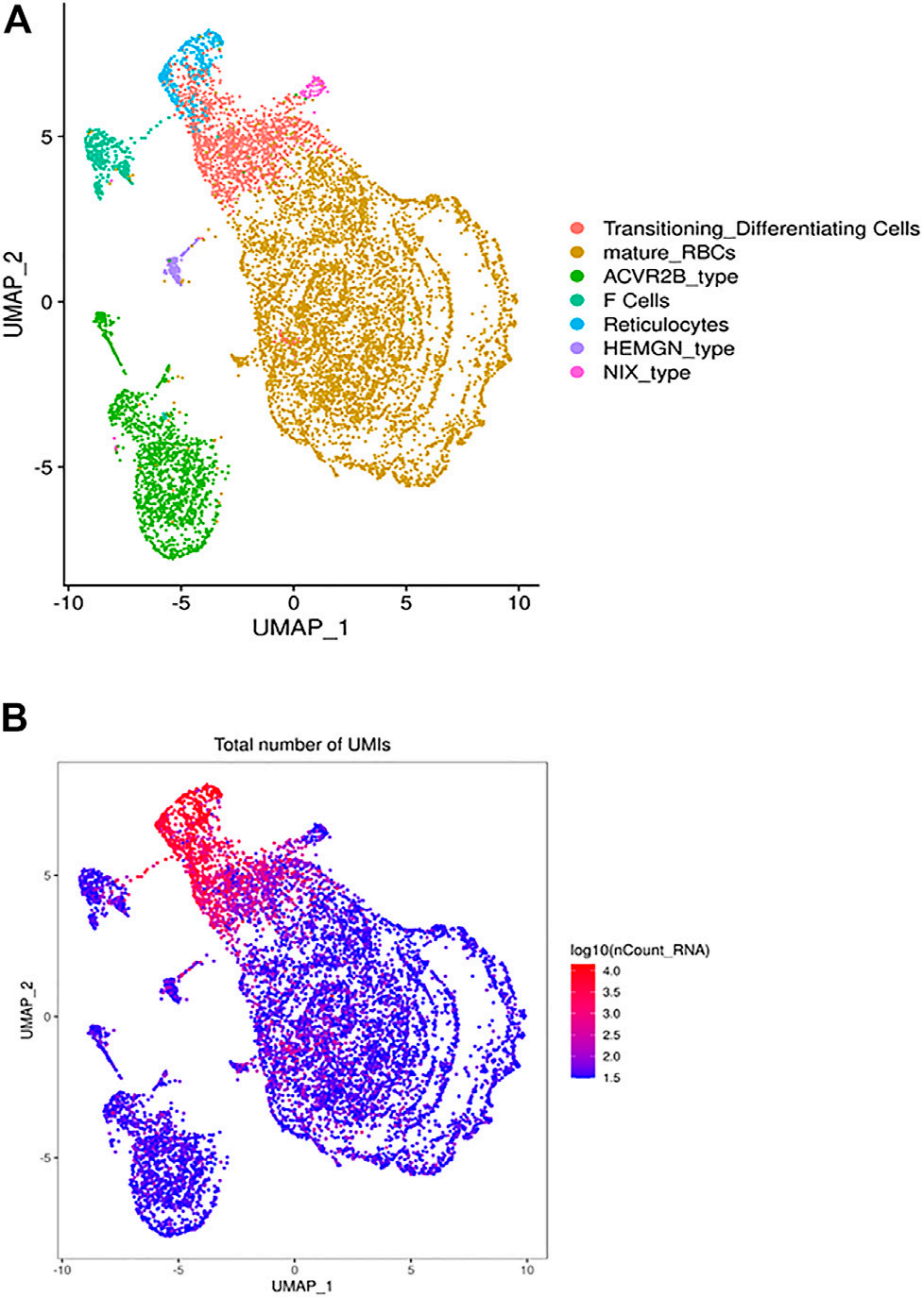
The heterogeneous clusters of the circulating RBCs and the number of expressed transcripts. Leukodepleted RBC units from three different individuals were incubated at 4°C for one and 15 days. Aliquots of RBCs were removed from the bag, centrifuged, subjected to the single cell RNA-Seq pipeline of Chromium. **(A)** The UMAP presentation of the heterogeneous clusters of RBCs with the clusters identified by expressed UMIs **(B)** and cluster-specific genes.

To define the single cell transcriptome of circulating red cells, blood samples were obtained from three healthy donors after consent according to the Institute of Review Board at Emory University. The blood samples were leukodepleted to remove leukocytes, washed and processed according to the protocols of American Red Cross. Aliquots of three leukodepleted AS-1 packed RBC units were sampled at day 1 (Day 1) and day 15 (D15) (Figure 1) (Sangokoya et al., 2010c). Drop-Seq analysis was carried out as recommended by 10x Genomics, but with a modification to the number of amplification cycles. Since each RBC contains significantly less RNA than regular nucleated cells, we increased the number of amplification cycles to generate cDNA library with broad size distribution, similar to other nucleated cells. The thus generated cDNA libraries were sequenced on an Illumina HiSeq 4000 at a mean depth of 58,061 reads/cell.

### Overall Clustering Patterns Based on the Gene Expression Patterns of Red Blood Cells

The UMAP analysis we identified 21 different clusters that were grouped into 7 cell types (Figure 1A) based on canonically expressed genes (Supplementary Table S1) and the relative levels of gene expression between the distinct clusters (Figure 1B). The distribution of UMIs in each individual cluster indicates that a small percentage of cells express 150–300 genes, include many ribosomal transcripts, while other RBCs expressed as few as 30 genes. Based on canonical and level of gene expression we annotated the transcriptionally active cluster as reticulocytes and young RBCs, while the adjacent lower expressing cluster represents mature RBCs. An intermediate cluster was characterized by the cluster-specific expression of large number of genes known to be expressed in RBCs, include many ribosomal transcripts. Further, this cluster is enriched for HBA1, HBA2 and HBM expression, which suggests that this cluster represents transitioning reticulocytes to mature RBCs (Figure 1A).

Next, we identified the cluster-specific genes (Supplementary Table S2) to generate a heatmap (Supplementary Figure S1) that further supported the further annotation of defined the RBC clusters. For example, one cluster is highly enriched in the expression of HBG2 and correspond to what are usually known as F cells. In the healthy individual, F-cell is expected to be present in 2–3% of the RBC, consistent with the 3.5% cells found in our analysis.

While the remaining RBC clusters have similar gene expression profiles they exhibit differential gene expression that may define RBC subtypes or at least expression state. For example, stratified clusters was enriched for expression of ACVR2B, which encodes Activin receptor type-2B, a transmembrane serine/threonine kinase receptor for activin. Activins are dimeric growth and differentiation TGF-beta family factors shown to regulate erythropoiesis. Luspatercept Acvr2b (L79D)-Fc) is a novel treatment for transfusion-dependent *β*-thalassemia patients that improves erythropoiesis. Another RBC cluster differentially expresses HEMGN, encoding hemogen or EDAG (Erythroid differentiation associated). EDAG is homologous to mouse Hemgn and rat RP59, is a hematopoietic specific transcriptional regulator involved in cell proliferation, differentiation, and apoptosis (Rodgers et al., 1985; Wang et al., 2013; Alapan et al., 2014). Finally, another cluster via its enriched expression of BNIP3L that encodes NIX which essential for the mitochondria clearance during the terminal differentiation of RBCs (Schweers et al., 2007; Sandoval et al., 2008).

### Hemoglobin Expression in Individual Red Blood Cells

**FIGURE 2:**
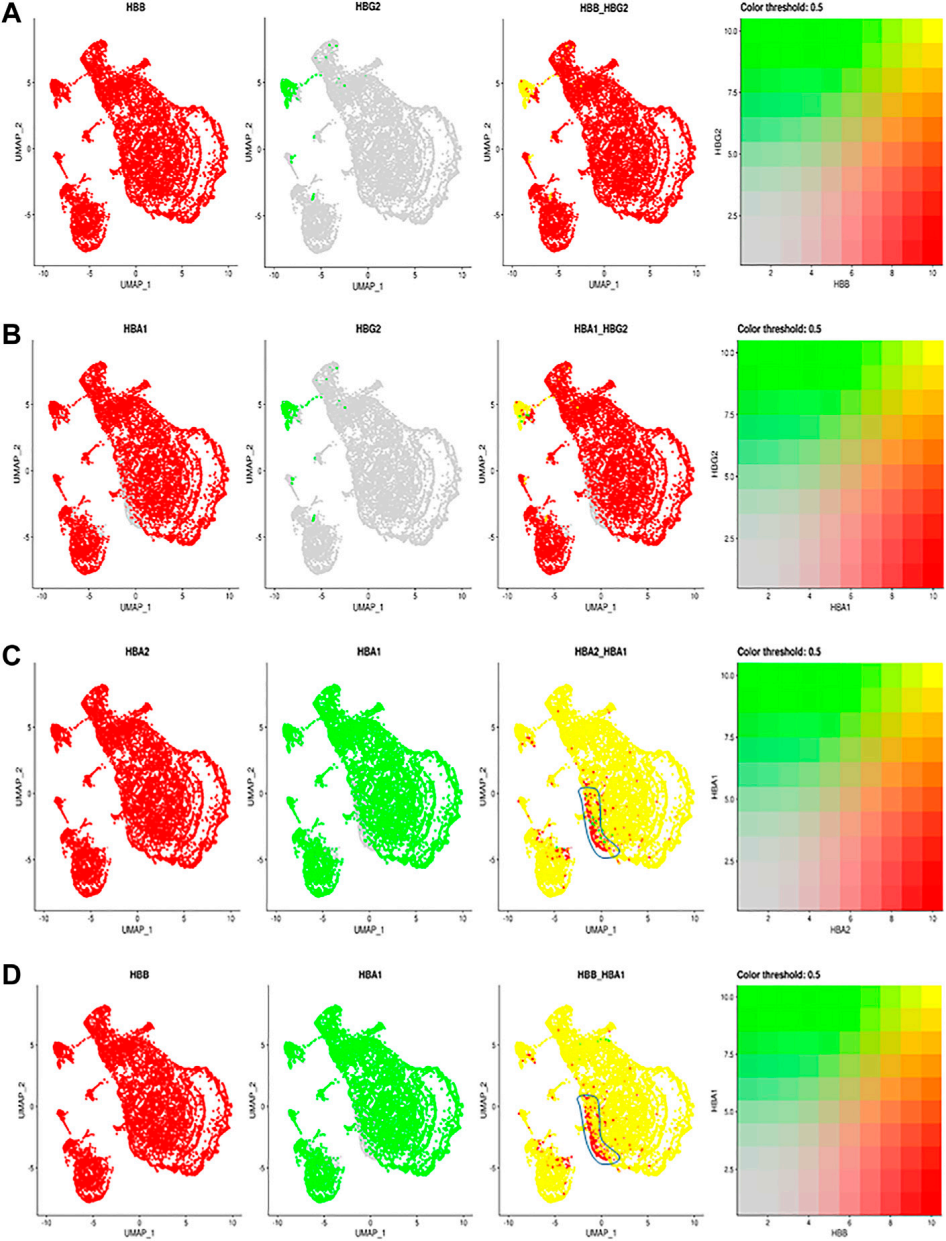
The comparisons and correlations of different hemoglobin genes in the individual RBCs. Pairwise comparison of hemoglobin genes within the overall UMAP projection including **(A)** HBB vs. HBG2 **(B)** HBA1 vs., HBG2 **(C)** HBA2 vs. HBA1 and **(D)** HBB vs. HBA, where each gene is assigned a color red or green and the intersection of expression is yellow, or the absence of expression is reflected in the primary gene color (right of each figure).

Given the importance of hemoglobins in RBC function, we next examined the allelic expression of individual hemoglobin transcripts in individual RBCs (Figure 2). We identified the expression of the two human fetal (*HBG2* and *HBG1*) globin genes within our annotated clusters (Figure 1A; Supplementary Figure S1A), including *HbG2* encoding fetal hemoglobin. Interestingly almost all RBCs in this cluster also express HbB (Figure 2A). During the hemoglobin switching, the locus control region (LCR) is supposed to switch from the fetal HbG locus to adult HbB (Sankaran and Orkin 2013). Since each LCR can interact with and activate the expression of either HbG2 or HbB gene at a time (Wijgerde et al., 1995), these data suggest that only one Hb allele has been switched from HBG2 to HBB, while the other allele still maintains the HBG2 expression. These results suggest an allelic specific hemoglobin switching in all the individual RBCs.

Other than F cells, almost all remaining RBC express both HBA2 and HBB (Figure 2B). Although most RBCs expressing HBA2 also express HBA1, a small portion of RBC cells in different RBC clusters only expressed HBA2 (Figures 2C,D, blue circle). These results indicate a modest heterogeneity in the allelic expression of the two HbA loci among different RBCs. The significance of such difference will require further exploration.

### The Changes of the Red Blood Cell Clustering During Red Blood Cell Storage

**FIGURE 3:**
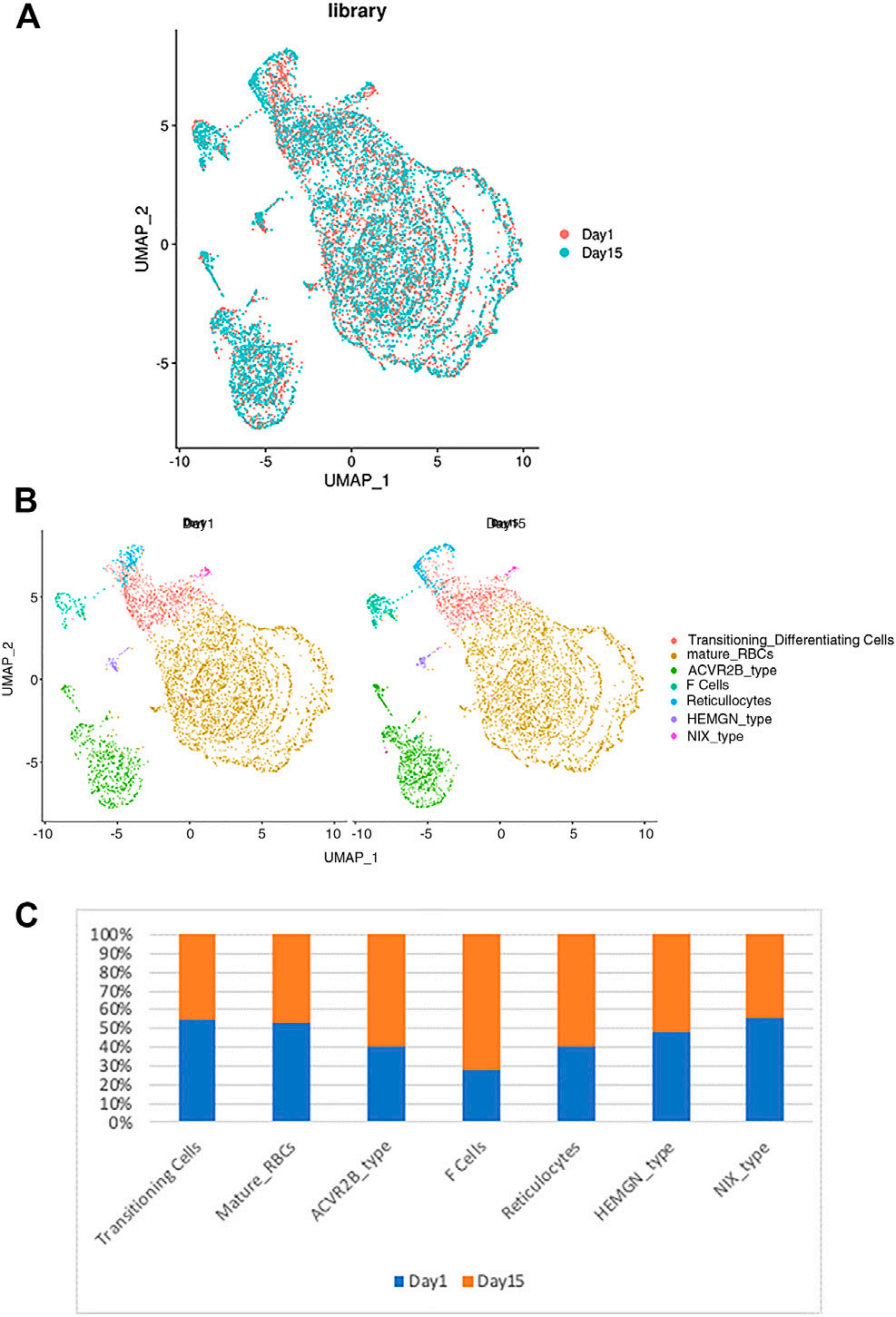
The relationship between the RBC clusters and storage times. **(A)** The indicated storage days of individual RBCs were projected into the UMAP clusters of RBCs. **(B)** The UMAP presentation of the heterogeneous clusters of separate RBCs which have been stored for one (left) or 15 days (right). **(C)** The relative frequency of the seven representative RBC clusters in day 1 vs. 15 showing an increase in ACVR2B and F-cells in *ex vivo* stored RBCs.

Next, we determined the effects of storage on RBC clustering by comparing the expression of day 1 vs. day 15 samples. As shown in Figure 3A, both day 1 and day 15 samples were well represented in each cluster of RBCs, indicating a similar RBC heterogeneity and lack of day-specific artifacts in the expression of RBCs. However, when the frequency distribution within different RBC clusters was compared between day 1 vs. day 15 samples, we noted that the sizes of several clusters are different among the day 1 vs. 15 samples (Figure 3B). When the relative number of cells within each cluster was compared between day 1 vs. 15 samples we noticed an increase in the number of F-cell and ACVR2B cells after 14 days of storage (Figure 3C). Enrichment in these RBC cell types suggests that they respond differently to *ex vivo* storage, which could have therapeutic implications and requires further study.

### Trajectory Analysis of Different Red Blood Cell Clusters

**FIGURE 4:**
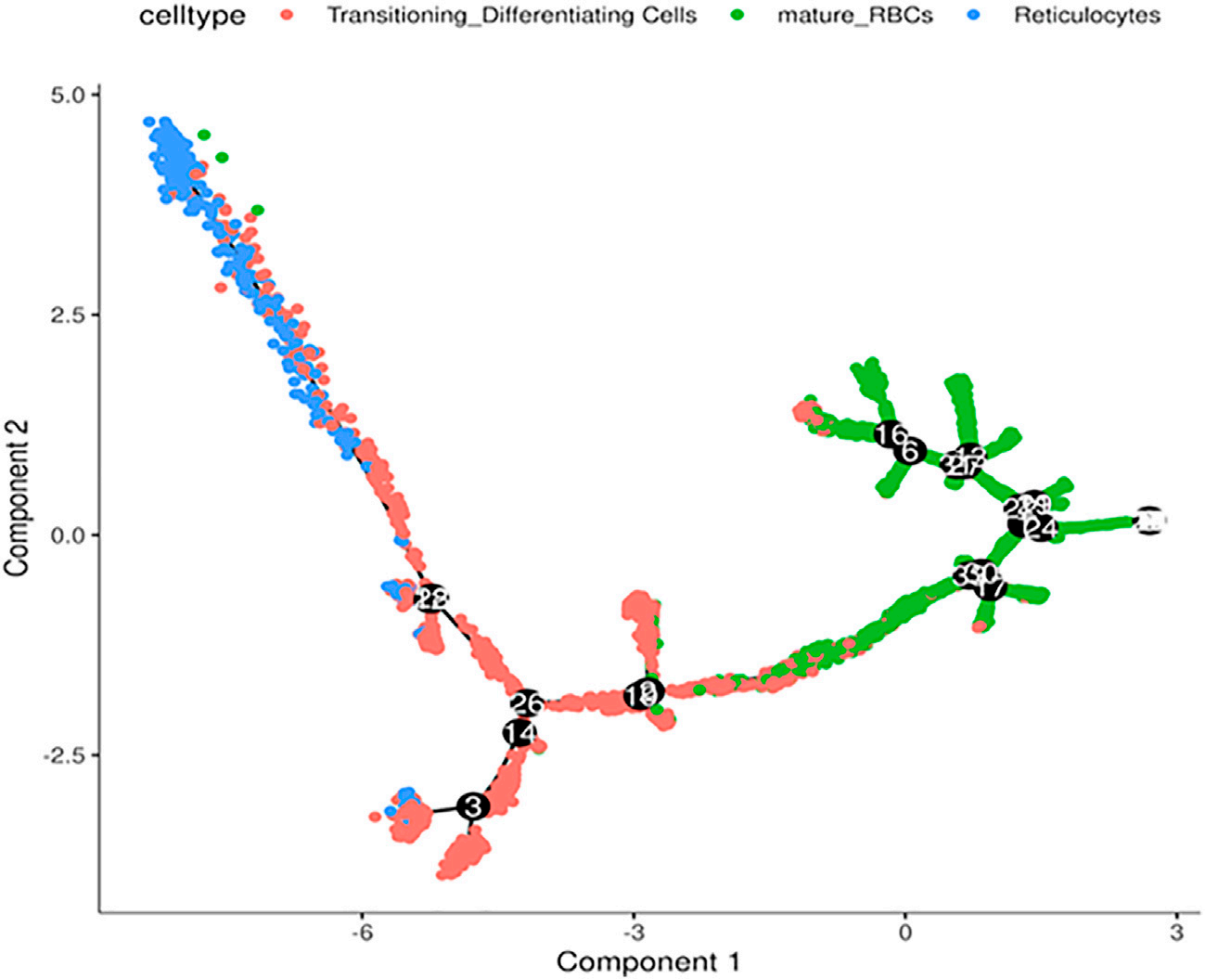
Trajectory analysis of differentiating RBCs. *In silico* analysis of differentiating RBC from reticulocytes to transitioning cells and mature RBCs colored separately.

Next, we performed *in silico* pseudotime trajectory analysis of the three main RBC clusters, including the reticulocytes, transitioning cells and mature RBCs. We found that the differentiation path was derived from reticulocytes to transitioning cells and finally mature RBC (Figure 4). This is consistent with the expected differentiation paths between these different RBC populations during terminal differentiation.

## Discussion

In this study, we have established that single RBC RNA-Seq can be used to identify heterogeneity among RBCs with seven distinct cell clusters. Based on the cluster-specific expression, we annotated reticulocytes and F cells as previously appreciated RBC sub-population. However, such analysis also identified some novel sub-populations which may suggest distinct RBC populations.

Conventional wisdom held that the human mature RBC do not contain DNA or RNA. However, during the past decade a large number of studies have shown the presence of abundant and diverse RNA species in the mature RBC (Chen et al., 2017). Importantly, the RBC transcriptome can serve as biomarkers for human diseases (Chen et al., 2017) and mediates the host-pathogen interactions (Walzer and Chi, 2017) and intercellular communication (Mantel et al., 2016). Furthermore, RBCs are a major source of the cell-free microRNAs that may complicate the findings of many blood-based biomarkers (McDonald et al., 2011). Despite overwhelming evidence, concerns remain as to whether RBC RNA may originate from contaminating reticulocytes. However, the data we describe herein not only establishes that reticulocytes express a diverse arrays of RNA species, but trajectory analysis of our scRNA-Seq data suggests that we can detect a continuum of expression from the reticulocytes to transitioning cells to mature RBCs (Figure 4). Therefore, our single cell analysis provides the most compelling evidence to date for the existence of dynamic RBC transcriptome that can be used for subtype analysis.

The RBC transcriptome has been used as biomarkers in a wide of variety of human diseases, including SCD, thalassemia and various auto-immune diseases (Chen et al., 2017). These studies are based on the expression analysis of the bulk cell analysis of the circulating RBCs or blood cells. The distinct RBC clusters identified in this study may allow us to quantify a specific cell population as novel biomarkers that would have been masked by previous bulk-cell analyses. For example, F cells have been used to monitor the severity and treatment response of sickle cell disease. Our approach may allow us to better characterize the proportion and distribution of RBCs in these clusters that may change during disease processes or treatments as more robust and reproducible source of biomarkers that characterize these processes. Another significant opportunity arising from our data is the identification of “storage-associated” cluster frequency differences that may represent the activation of specific pathways to prolong the life span of RBCs and mimic the “aging” processes. Further, the expression profile of yet other clusters of cells from our analysis may yield insight into a better understanding of the pathology of hemolytic anemia because of their expression of auto-antigens or reduced anti-stress capacity as reflected by their gene expression.

Some of the cluster-specific genes may suggest the distinct development pathway of these RBCs as illustrated by the cluster 5 (ACVR2B) and 6 (HEMGN). For example, cluster 6 is distinct based on the expression of HEMGN, encoding hemogen or EDAG (Erythroid differentiation associated). EDAG is homologous to mouse Hemgn and rat RP59, is a hematopoietic specific transcriptional regulator involved in cell proliferation, differentiation, and apoptosis (Rodgers et al., 1985; Wang et al., 2013; Alapan et al., 2014). Overexpression of EDAG induced erythroid differentiation of CD34 ^+^ cells *in vitro* and *in vivo* using immunodeficient mice. Conversely, EDAG knockdown reduced erythroid differentiation in EPO‐treated CD34 ^+^ cells. Another cluster has elevated expression of ACVR2B, which encodes Activin receptor type-2B Transmembrane serine/threonine kinase activin receptor. The Fc fusion of ACVR2B can be used to boost the late-stage erythrocyte (red blood cell) precursor cell differentiation and maturation independent of EPO pathways (Sherman et al., 2013; Attie et al., 2014). Finally, the cluster-specific expression of BNIP3L, which encodes NIX essential for the mitochondria clearance during the terminal differentiation of RBCs (Schweers et al., 2007; Sandoval et al., 2008). This cluster may, therefore, represent RBCs with varying levels of NIX transcripts and mitochondrial clearance.

### Limitation of Red Blood Cell RNA Transcriptome

It is important to point out some important limitation of our study. First, given the lack of robust translation in mature RBCs, the cluster-specific mRNAs we have identified may not be readily lead to corresponding protein expression and functional phenotypes. In addition, there are significantly fewer transcripts per RBC, compared with the other nucleated cells. This lower number of transcripts leads to a simpler principal component structure, leading to a less robust clustering pattern more easily effected by stochastic processes. Finally, our analysis focus on polyA + mRNA, which is not the most abundant RNA species in the mature RBC whereas the majority of the RBCs are microRNAs and other small-sized RNA. Therefore, the classification by the mRNAs may not fully capture RBC heterogeneity as defined by the microRNA transcriptome. Therefore, future works will incorporate the microRNA expression patterns to further refine the RBC heterogeneity to gain a better understanding of their functional relevance in the developmental history and functional relevance.

## Data Availability

The datasets presented in this study can be found in online repositories. The names of the repository/repositories and accession number(s) can be found below: https://www.ncbi.nlm.nih.gov/geo/query/acc.cgi?acc=GSE184916.

## Footnotes

1 Cell Rangers of 10X Genomics.

2 Seurat R Package.

## References

[B1] Alapan Y. Little J. A. Gurkan U. A. (2014). Heterogeneous Red Blood Cell Adhesion and Deformability in Sickle Cell Disease. Sci. Rep. 4, 7173. 10.1038/srep07173 25417696 PMC4241514

[B2] Antonelou M. H. Kriebardis A. G. Papassideri I. S. (2010). Aging and Death Signalling in Mature Red Cells: From Basic Science to Transfusion Practice. Blood Transfus. 8 Suppl 3 (Suppl. 3), s39–47. 10.2450/2010.007S 20606748 PMC2897187

[B3] Attie K. M. Allison M. J. McClure T. Boyd I. E. Wilson D. M. Pearsall A. E. (2014). A Phase 1 Study of ACE‐536, a Regulator of Erythroid Differentiation, in Healthy Volunteers. Am. J. Hematol. 89, 766–770. 10.1002/ajh.23732 24715706 PMC4173124

[B4] Azzouzi I. Moest H. Wollscheid B. Schmugge M. Eekels J. J. M. Speer O. (2015). Deep Sequencing and Proteomic Analysis of the microRNA-Induced Silencing Complex in Human Red Blood Cells. Exp. Hematol. 43, 382–392. 10.1016/j.exphem.2015.01.007 25681748

[B5] Belcher J. D. Chen C. Nguyen J. Zhang P. Abdulla F. Nguyen P. (2017). Control of Oxidative Stress and Inflammation in Sickle Cell Disease with the Nrf2 Activator Dimethyl Fumarate. Antioxid. Redox Signaling 26, 748–762. 10.1089/ars.2015.6571 PMC5421647 26914345

[B6] Bennett-Guerrero E. Veldman T. H. Doctor A. Telen M. J. Ortel T. L. Reid T. S. (2007). Evolution of Adverse Changes in Stored RBCs. Proc. Natl. Acad. Sci. U.S.A. 104, 17063–17068. 10.1073/pnas.0708160104 17940021 PMC2040393

[B7] Berezina T. L. Zaets S. B. Morgan C. Spillert C. R. Kamiyama M. Spolarics Z. (2002). Influence of Storage on Red Blood Cell Rheological Properties. J. Surg. Res. 102, 6–12. 10.1006/jsre.2001.6306 11792145

[B8] Chen P.-H. Hong J. Chi J.-T. (2017). Discovery, Genomic Analysis, and Functional Role of the Erythrocyte RNAs. Curr. Pathobiology Rep., 1–6. 10.1007/s40139-017-0124-z

[B9] Chen S.-Y. Wang Y. Telen M. J. Chi J.-T. (2008). The Genomic Analysis of Erythrocyte microRNA Expression in Sickle Cell Diseases. PLoS One 3, e2360. 10.1371/journal.pone.0002360 18523662 PMC2408759

[B10] D'Alessandro A. Liumbruno G. Grazzini G. Zolla L. (2010). Red Blood Cell Storage: The Story So Far. Blood Transfus. 8, 82–88. 10.2450/2009.0122-09 20383300 PMC2851210

[B11] D'Alessandro A. Kriebardis A. G. Rinalducci S. Antonelou M. H. Hansen K. C. Papassideri I. S. (2015). An Update on Red Blood Cell Storage Lesions, as Gleaned Through Biochemistry and Omics Technologies. Transfusion 55, 205–219. 10.1111/trf.12804 25130459

[B12] Doss J. F. Corcoran D. L. Jima D. D. Telen M. J. Dave S. S. Chi J.-T. (2015). A Comprehensive Joint Analysis of the Long and Short RNA Transcriptomes of Human Erythrocytes. BMC Genomics 16, 952. 10.1186/s12864-015-2156-2 26573221 PMC4647483

[B13] Doss J. F. Jonassaint J. C. Garrett M. E. Ashley-Koch A. E. Telen M. J. Chi J.-T. (2016). Phase 1 Study of a Sulforaphane-Containing Broccoli Sprout Homogenate for Sickle Cell Disease. PLoS One 11, e0152895. 10.1371/journal.pone.0152895 27071063 PMC4829228

[B14] Driss A. Asare K. O. Hibbert J. M. Gee B. E. Adamkiewicz T. V. Stiles J. K. (2009). Sickle Cell Disease in the Post Genomic Era: A Monogenic Disease with a Polygenic Phenotype. Genomics Insights 2009, 23–48. 10.4137/gei.s2626 20401335 PMC2855197

[B15] Kannan M. Atreya C. (2010). Differential Profiling of Human Red Blood Cells During Storage for 52 Selected microRNAs. Transfusion 50, 1581–1588. 10.1111/j.1537-2995.2010.02585.x 20158686

[B16] Kim‐Shapiro D. B. Lee J. Gladwin M. T. (2011). Storage Lesion: Role of Red Blood Cell Breakdown. Transfusion 51, 844–851. 21496045 10.1111/j.1537-2995.2011.03100.x PMC3080238

[B17] Macosko E. Z. Basu A. Satija R. NemeshShekhar J. K. ShekharBialas K. I. Tirosh A. R. (2015). Highly Parallel Genome-Wide Expression Profiling of Individual Cells Using Nanoliter Droplets. Cell 161, 1202–1214. 10.1016/j.cell.2015.05.002 26000488 PMC4481139

[B18] Mantel P.-Y. Hjelmqvist D. Walch M. Kharoubi-Hess S. Nilsson S. Ravel D. (2016). Infected Erythrocyte-Derived Extracellular Vesicles Alter Vascular Function via Regulatory Ago2-miRNA Complexes in Malaria. Nat. Commun. 7, 12727. 10.1038/ncomms12727 27721445 PMC5062468

[B19] McDonald J. S. Milosevic D. Reddi H. V. Grebe S. K. Algeciras-Schimnich A. (2011). Analysis of Circulating microRNA: Preanalytical and Analytical Challenges. Clin. Chem. 57, 833–840. 10.1373/clinchem.2010.157198 21487102

[B20] McInnes L. Healy J. Melville J. (2018). UMAP: Uniform Manifold Approximation and Projection for Dimension Reduction, 03426. arXiv 1802.

[B21] Monocle2 Documentation.

[B22] World Health Organization (2014). Blood Safety and Availability. Geneva, Switzerland: WHO Fact Sheet.

[B23] Park H.-S. Price H. Ceballos S. Chi J.-T. Wax A. (2021). Single Cell Analysis of Stored Red Blood Cells Using Ultra-High Throughput Holographic Cytometry. Cells 10, 2455. 10.3390/cells10092455 34572104 PMC8465484

[B24] Park H. S. Eldridge W. J. Yang W.-H. Crose M. Ceballos S. Roback J. D. (2019). Quantitative Phase Imaging of Erythrocytes Under Microfluidic Constriction in a High Refractive Index Medium Reveals Water Content Changes. Microsyst. Nanoeng. 5, 63. 10.1038/s41378-019-0113-y 31814994 PMC6885519

[B25] Pauling L. Itano H. A. Singer S. J. Wells I. C. (1949). Sickle Cell Anemia, a Molecular Disease. Science 110, 543–548. 10.1126/science.110.2865.543 15395398

[B26] Picas L. Rico F. Deforet M. Scheuring S. (2013). Structural and Mechanical Heterogeneity of the Erythrocyte Membrane Reveals Hallmarks of Membrane Stability. ACS Nano 7, 1054–1063. 10.1021/nn303824j 23347043

[B27] Piel F. B. Steinberg M. H. Rees D. C. (2017). Sickle Cell Disease. N. Engl. J. Med. 376, 1561–1573. 10.1056/nejmra1510865 28423290

[B28] Qiu X. Mao Q. Tang Y. Wang L. Chawla R. Pliner H. A. (2017). Reversed Graph Embedding Resolves Complex Single-Cell Trajectories. Nat. Methods 14, 979–982. 10.1038/nmeth.4402 28825705 PMC5764547

[B29] Rathjen T. Nicol C. McConkey G. Dalmay T. (2006). Analysis of Short RNAs in the Malaria Parasite and its Red Blood Cell Host. FEBS Lett. 580, 5185–5188. 10.1016/j.febslet.2006.08.063 16963026

[B30] Rodgers G. P. Schechter A. N. Noguchi C. T. (1985). Cell Heterogeneity in Sickle Cell Disease: Quantitation of the Erythrocyte Density Profile. J. Lab. Clin. Med. 106, 30–37. 4009021

[B31] Sandoval H. Thiagarajan P. Dasgupta S. K. Schumacher A. Prchal J. T. Chen M. (2008). Essential Role for Nix in Autophagic Maturation of Erythroid Cells. Nature 454, 232–235. 10.1038/nature07006 18454133 PMC2570948

[B32] Sangokoya C. LaMonte G. Chi J.-T. (2010). Isolation and Characterization of microRNAs of Human Mature Erythrocytes. Methods Mol. Biol. 667, 193–203. 10.1007/978-1-60761-811-9_13 20827535 PMC4347925

[B33] Sangokoya C. LaMonte G. Chi J.-T. (2010). Isolation and Characterization of microRNAs of Human Mature Erythrocytes. Methods Mol. Biol. (Clifton, N.J.) 667, 193–203. 10.1007/978-1-60761-811-9_13 PMC4347925 20827535

[B34] Sangokoya C. Telen M. J. Chi J.-T. (2010). microRNA miR-144 Modulates Oxidative Stress Tolerance and Associates with Anemia Severity in Sickle Cell Disease. Blood 116, 4338–4348. 10.1182/blood-2009-04-214817 20709907 PMC2993631

[B35] Sankaran V. G. Orkin S. H. (2013). The Switch from Fetal to Adult Hemoglobin. Cold Spring Harbor Perspect. Med. 3, a011643. 10.1101/cshperspect.a011643 PMC3530042 23209159

[B36] Sarachana T. Kulkarni S. Atreya C. D. (2015). Evaluation of Small Noncoding RNAs in *Ex Vivo* Stored Human Mature Red Blood Cells: Changes in Noncoding RNA Levels Correlate with Storage Lesion Events Transfusion. 10.1111/trf.13235 26174076

[B37] Schweers R. L. Zhang J. Randall M. S. Loyd M. R. Li W. Dorsey F. C. (2007). NIX Is Required for Programmed Mitochondrial Clearance during Reticulocyte Maturation. Proc. Natl. Acad. Sci. U.S.A. 104, 19500–19505. 10.1073/pnas.0708818104 18048346 PMC2148318

[B38] Setup the Seurat Object 2011. Seurat - Guided Clustering Tutorial.

[B39] Sherman M. L. Borgstein N. G. Mook L. Wilson D. Yang Y. Chen N. (2013). Multiple‐dose, Safety, Pharmacokinetic, and Pharmacodynamic Study of Sotatercept (ActRIIA‐IgG1), a Novel Erythropoietic Agent, in Healthy Postmenopausal Women. J. Clin. Pharmacol. 53, 1121–1130. 10.1002/jcph.160 23939631

[B40] Tinmouth A. Fergusson D. Yee I. C. Hébert P. C. (2006). Clinical Consequences of Red Cell Storage in the Critically Ill. Transfusion 46, 2014–2027. 10.1111/j.1537-2995.2006.01026.x 17076859

[B41] Walzer K. A. Kubicki D. M. Tang X. Chi J. T. (2018). Single-Cell Analysis Reveals Distinct Gene Expression and Heterogeneity in Male and Female Plasmodium Falciparum Gametocytes. mSphere 3. 10.1128/mSphere.00130-18 PMC5909122 29643077

[B42] Walzer K. A. Fradin H. Emerson L. Y. Corcoran D. L. Chi J.-T. (2019). Latent Transcriptional Variations of Individual Plasmodium Falciparum Uncovered by Single-Cell RNA-Seq and Fluorescence Imaging. Plos Genet. 15, e1008506. 10.1371/journal.pgen.1008506 31856180 PMC6952112

[B43] Walzer K. A. Chi J. T. (2017). Trans-Kingdom Small RNA Transfer During Host-Pathogen Interactions: The Case of *P. falciparum* and Erythrocytes. RNA Biology (Abingdon, United Kingdom: Taylor & Francis Group) 14 (4), 442–449. 10.1080/15476286.2017.1294307 28277932 PMC5411123

[B44] Wang J. Wagner-Britz L. Bogdanova A. Ruppenthal S. Wiesen K. Kaiser E. (2013). Morphologically Homogeneous Red Blood Cells Present a Heterogeneous Response to Hormonal Stimulation. PLoS One 8, e67697. 10.1371/journal.pone.0067697 23840765 PMC3695909

[B45] Wijgerde M. Grosveld F. Fraser P. (1995). Transcription Complex Stability and Chromatin Dynamics *In Vivo* . Nature 377, 209–213. 10.1038/377209a0 7675106

[B46] Wolfe L. (1985). The Membrane and the Lesions of Storage in Preserved Red Cells. Transfusion 25, 185–203. 10.1046/j.1537-2995.1985.25385219897.x 3890284

[B47] Xue X. Zhang Q. Huang Y. Feng L. Pan W. (2008). No miRNA Were Found in Plasmodium and the Ones Identified in Erythrocytes Could Not Be Correlated with Infection. Malar. J. 7, 47. 10.1186/1475-2875-7-47 18328111 PMC2329658

[B48] Yang W. H. Doss J. F. Walzer K. A. McNulty S. M. Wu J. Roback J. D. (2018). Angiogenin-Mediated tRNA Cleavage as a Novel Feature of Stored Red Blood Cells. Br. J. Haematol. 10.1111/bjh.15605 PMC6472987 30368767

[B49] Zheng G. X. Y. Terry J. M. Belgrader P. Ryvkin P. Bent Z. W. Wilson R. (2017). Massively Parallel Digital Transcriptional Profiling of Single Cells. Nat. Commun. 8, 14049. 10.1038/ncomms14049 28091601 PMC5241818

