# Supplementary material for: Single Cell RNA-Seq Analysis of Human Red Cells
Source: Front Physiol. 2022 Apr 20;13:828700. doi: 10.3389/fphys.2022.828700 (PMC9065680; doi:10.3389/fphys.2022.828700)
Supplement: Supplementary file 1 [file DataSheet2.PDF]

| | p_val | avg_log2FC | pct.1 | pct.2 | p_val_adj | cluster | gene |
|---|---|---|---|---|---|---|---|
| RPL21 | 0 | 2.3123597 | 0.488 | 0.061 | 0 | Transitioning_Differentiating Cells | RPL21 |
| UBA52 | 0 | 2.200099439 | 0.721 | 0.099 | 0 | Transitioning_Differentiating Cells | UBA52 |
| RPS12 | 0 | 2.17885211 | 0.596 | 0.08 | 0 | Transitioning_Differentiating Cells | RPS12 |
| SLC25A37 | 0 | 2.092146743 | 0.649 | 0.088 | 0 | Transitioning_Differentiating Cells | SLC25A37 |
| HBA2 | 0 | 0.787653806 | 1 | 1 | 0 | Transitioning_Differentiating Cells | HBA2 |
| RPL41 | 1.77E-273 | 2.186363127 | 0.291 | 0.034 | 3.75E-270 | Transitioning_Differentiating Cells | RPL41 |
| RPLP2 | 2.04E-183 | 2.039839702 | 0.191 | 0.021 | 4.31E-180 | Transitioning_Differentiating Cells | RPLP2 |
| SERF2 | 2.53E-177 | 1.354848734 | 0.257 | 0.044 | 5.35E-174 | Transitioning_Differentiating Cells | SERF2 |
| TPT1 | 2.18E-163 | 1.074457662 | 0.24 | 0.04 | 4.62E-160 | Transitioning_Differentiating Cells | TPT1 |
| RPS15A | 3.51E-133 | 2.463580161 | 0.141 | 0.016 | 7.43E-130 | Transitioning_Differentiating Cells | RPS15A |
| YBX1 | 1.89E-123 | 1.002956086 | 0.183 | 0.032 | 4.00E-120 | Transitioning_Differentiating Cells | YBX1 |
| RPL32 | 4.15E-119 | 1.870033605 | 0.132 | 0.015 | 8.78E-116 | Transitioning_Differentiating Cells | RPL32 |
| RPS11 | 1.36E-114 | 1.515908824 | 0.134 | 0.017 | 2.88E-111 | Transitioning_Differentiating Cells | RPS11 |
| HBM | 1.61E-114 | 0.994438736 | 0.184 | 0.034 | 3.42E-111 | Transitioning_Differentiating Cells | HBM |
| RPL35A | 1.48E-111 | 1.932556854 | 0.12 | 0.013 | 3.14E-108 | Transitioning_Differentiating Cells | RPL35A |
| AC130456.3 | 3.04E-103 | 1.096234156 | 0.122 | 0.015 | 6.45E-100 | Transitioning_Differentiating Cells | AC130456.3 |
| RPL38 | 8.61E-97 | 1.719556785 | 0.111 | 0.013 | 1.83E-93 | Transitioning_Differentiating Cells | RPL38 |
| RPS14 | 1.71E-91 | 1.168512115 | 0.117 | 0.017 | 3.62E-88 | Transitioning_Differentiating Cells | RPS14 |
| PFDN5 | 4.70E-90 | 1.170500787 | 0.118 | 0.017 | 9.96E-87 | Transitioning_Differentiating Cells | PFDN5 |
| RPL30 | 7.17E-88 | 1.028717547 | 0.123 | 0.019 | 1.52E-84 | Transitioning_Differentiating Cells | RPL30 |
| RPLP1 | 6.25E-87 | 0.770253027 | 0.114 | 0.017 | 1.32E-83 | Transitioning_Differentiating Cells | RPLP1 |
| RPL12 | 4.61E-86 | 1.395500079 | 0.119 | 0.019 | 9.76E-83 | Transitioning_Differentiating Cells | RPL12 |
| RPS15 | 1.31E-81 | 1.205895037 | 0.114 | 0.018 | 2.77E-78 | Transitioning_Differentiating Cells | RPS15 |
| BTF3 | 7.17E-79 | 1.221622025 | 0.122 | 0.022 | 1.52E-75 | Transitioning_Differentiating Cells | BTF3 |
| HBA1 | 1.09E-78 | 0.441552168 | 0.999 | 0.968 | 2.31E-75 | Transitioning_Differentiating Cells | HBA1 |
| ATP5F1E | 3.42E-77 | 0.824765375 | 0.136 | 0.028 | 7.25E-74 | Transitioning_Differentiating Cells | ATP5F1E |
| BAG1 | 2.94E-60 | 0.797010704 | 0.12 | 0.028 | 6.24E-57 | Transitioning_Differentiating Cells | BAG1 |
| BNIP3L | 1.17E-51 | 0.531257538 | 0.118 | 0.03 | 2.47E-48 | Transitioning_Differentiating Cells | BNIP3L |
| HBB | 0 | 0.299615028 | 1 | 1 | 0 | mature_RBCs | HBB |
| GUK1 | 1.16E-86 | 0.515561187 | 0.031 | 0.141 | 2.47E-83 | mature_RBCs | GUK1 |
| OAZ1 | 2.55E-76 | 0.412951952 | 0.054 | 0.177 | 5.40E-73 | mature_RBCs | OAZ1 |
| SLC25A39 | 1.73E-61 | 0.514347279 | 0.099 | 0.239 | 3.66E-58 | mature_RBCs | SLC25A39 |
| UBB | 5.41E-57 | 0.49951806 | 0.044 | 0.138 | 1.15E-53 | mature_RBCs | UBB |
| ALAS2 | 5.65E-36 | 0.697157666 | 0.037 | 0.102 | 1.20E-32 | mature_RBCs | ALAS2 |

| | | | | | | | |
|---|---|---|---|---|---|---|---|
| DONSON | 6.89E-05 | 0.648043009 | 0.157 | 0.135 | 0.145955964 | mature_RBCs | DONSON |
| GPM6A | 0 | 5.050929112 | 0.212 | 0.007 | 0 | ACVR2B_type | GPM6A |
| ACVR2B | 0 | 4.954955811 | 0.25 | 0.008 | 0 | ACVR2B_type | ACVR2B |
| BDH1 | 0 | 4.922396364 | 0.3 | 0.011 | 0 | ACVR2B_type | BDH1 |
| PIGN | 2.04E-281 | 5.086417658 | 0.176 | 0.005 | 4.31E-278 | ACVR2B_type | PIGN |
| GABRE | 4.90E-242 | 5.006105292 | 0.147 | 0.003 | 1.04E-238 | ACVR2B_type | GABRE |
| OAZ11 | 1.27E-11 | 0.280719602 | 0.051 | 0.111 | 2.69E-08 | ACVR2B_type | OAZ1 |
| FTL | 6.33E-10 | 0.273007887 | 0.156 | 0.249 | 1.34E-06 | ACVR2B_type | FTL |
| GYPC | 9.36E-07 | 0.417583944 | 0.117 | 0.18 | 0.001982529 | ACVR2B_type | GYPC |
| DONSON1 | 0.000238592 | 0.442462297 | 0.178 | 0.143 | 0.505577401 | ACVR2B_type | DONSON |
| HBG2 | 0 | 6.805282983 | 0.823 | 0.005 | 0 | F Cells | HBG2 |
| BTF31 | 9.78E-59 | 4.383827826 | 0.189 | 0.03 | 2.07E-55 | F Cells | BTF3 |
| HBD | 0 | 1.108903666 | 0.374 | 0.012 | 0 | Reticulocytes | HBD |
| FAM210B | 0 | 0.916359977 | 0.535 | 0.009 | 0 | Reticulocytes | FAM210B |
| SNCA | 0 | 0.689578752 | 0.516 | 0.016 | 0 | Reticulocytes | SNCA |
| RNF10 | 0 | 0.495450547 | 0.487 | 0.007 | 0 | Reticulocytes | RNF10 |
| ADIPOR1 | 0 | 0.490993401 | 0.716 | 0.023 | 0 | Reticulocytes | ADIPOR1 |
| GUCD1 | 0 | 0.455970438 | 0.187 | 0.001 | 0 | Reticulocytes | GUCD1 |
| MKRN1 | 0 | 0.393542103 | 0.481 | 0.01 | 0 | Reticulocytes | MKRN1 |
| DCAF12 | 0 | 0.382004219 | 0.506 | 0.013 | 0 | Reticulocytes | DCAF12 |
| GMPR | 3.36E-301 | 0.585670154 | 0.177 | 0.001 | 7.13E-298 | Reticulocytes | GMPR |
| AHSP | 7.57E-215 | 0.283658062 | 0.31 | 0.017 | 1.60E-211 | Reticulocytes | AHSP |
| PITHD1 | 9.16E-162 | 0.285151009 | 0.11 | 0.001 | 1.94E-158 | Reticulocytes | PITHD1 |
| BPGM | 2.42E-145 | 0.442630287 | 0.11 | 0.002 | 5.12E-142 | Reticulocytes | BPGM |
| HBA11 | 5.58E-91 | 0.791267207 | 1 | 0.971 | 1.18E-87 | Reticulocytes | HBA1 |
| HBA21 | 4.14E-69 | 0.392488987 | 1 | 1 | 8.76E-66 | Reticulocytes | HBA2 |
| HEMGN | 0 | 7.643992512 | 1 | 0.009 | 0 | HEMGN_type | HEMGN |
| BNIP3L1 | 0 | 6.554557301 | 0.988 | 0.035 | 0 | NIX_type | BNIP3L |
