# Supplementary figures and images for: Single Cell RNA-Seq Analysis of Human Red Cells
Source: Front Physiol. 2022 Apr 20;13:828700. doi: 10.3389/fphys.2022.828700 (PMC9065680; doi:10.3389/fphys.2022.828700)

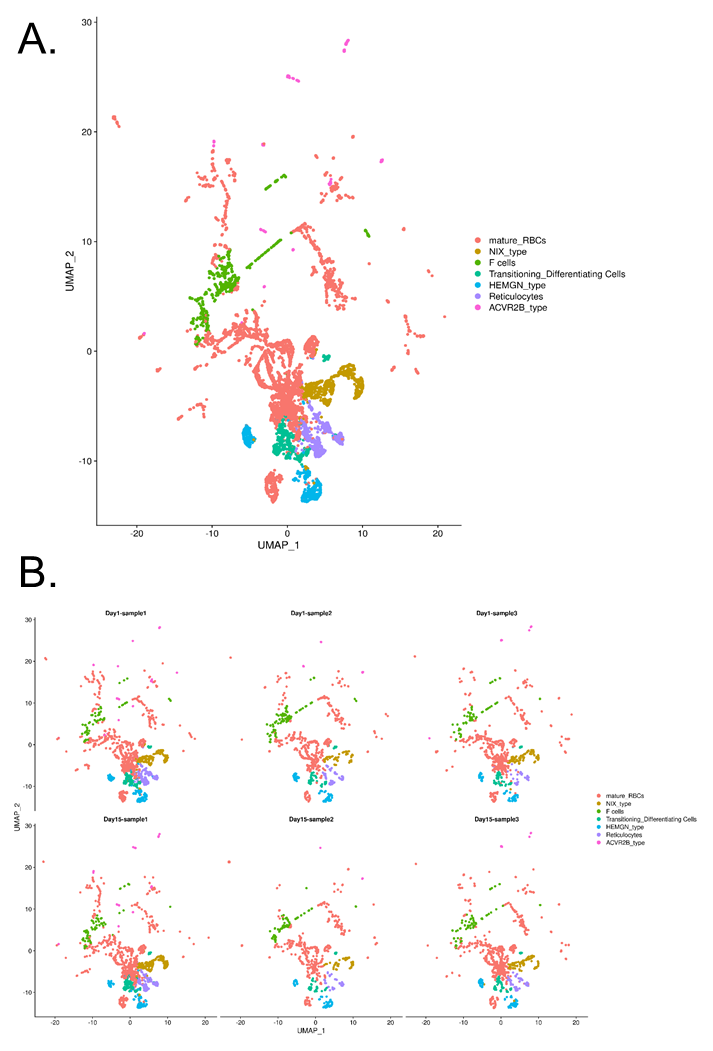

Supplement: Supplementary file 2 [file Image2.tif]

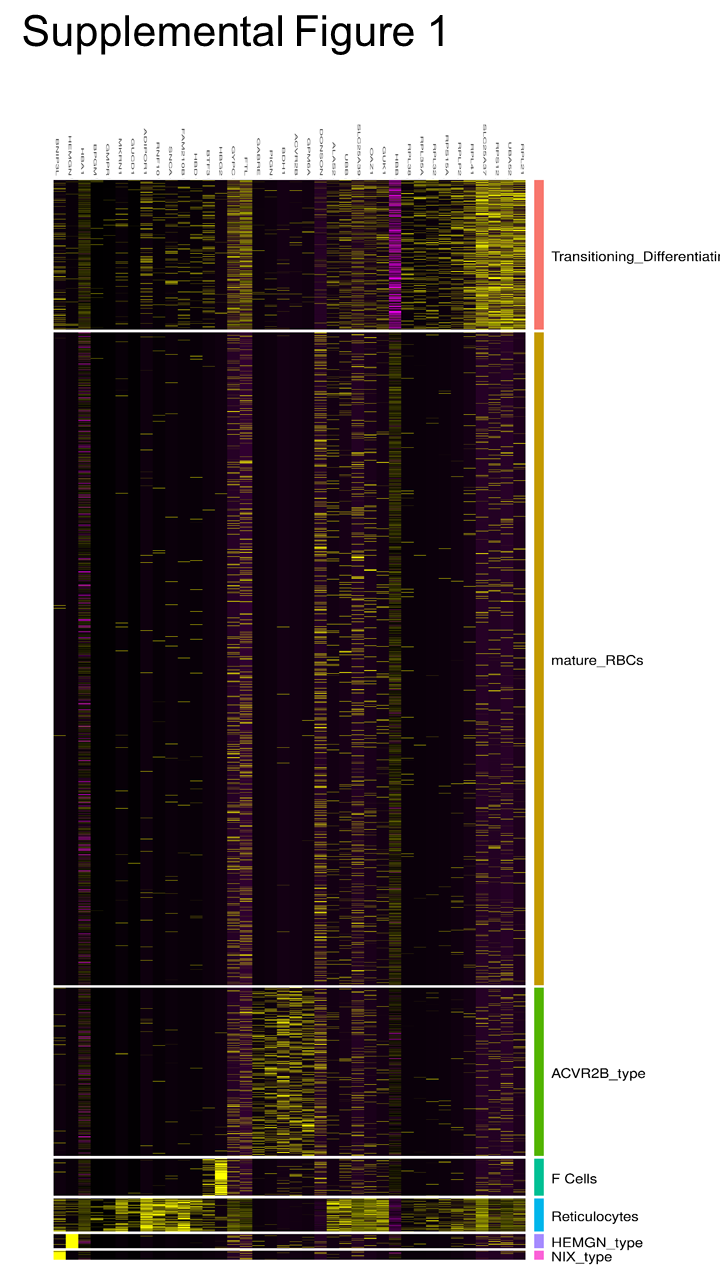

Supplement: Supplementary file 3 [file Image1.TIF]
