# Supplementary material for: Single Cell RNA-Seq Analysis of Human Red Cells
Source: Front Physiol. 2022 Apr 20;13:828700. doi: 10.3389/fphys.2022.828700 (PMC9065680; doi:10.3389/fphys.2022.828700)
Supplement: Supplementary file 4 [file DataSheet1.PDF]

	RNA.Transitioning_Di	RNA.mature_RBCs	RNA.ACVR2B_type	RNA.F.Cells	RNA.Reticulocytes	RNA.HEMC	RNA.NIX_t
FAM41C	0	0	0	0	0.004834842	0	0
MRPL20	0	0	0	0	0.003587418	0	0
MMP23B	0	0	0	0	0.003564033	0	0
SLC35E2B	0	0	0	0	0.003248546	0	0
FAAP20	0	0	0	0	0.003090445	0	0
RER1	0	0	0	0	0.004168247	0	0
PEX10	0.001199548	0	0	0	0	0	0
TPRG1L	0	0	0	0	0.003583831	0	0
SMIM1	0.37276784	0.218308674	0.957701197	0	0.146361036	0	0
RPL22	0.226834597	0.102191874	0.100398582	0.579491056	0.082945626	0	0
ESPN	0.004749897	0	0	0	0.035112591	0	0
UBE4B	0	0.047925313	0.112948405	0	0.005313468	0	0
DFFA	0	0.026281623	0	0	0.004517936	0	0
MIIP	0	0	0	0	0.005885302	0	0
DDI2	0	0	0	0	0.009490954	0	0
SZRD1	0.01364676	0.038796682	0	0	0.006529973	0	0
NECAP2	0	0	0	0	0.003926249	0	0
SDHB	0.002742022	0	0	0	0	0	0
UBXN10	0	0	0	0	0.003821593	0	0
PINK1	0.000944118	0.100176621	0	0	0.036648263	0	0
HP1BP3	0	0.036210237	0	0	0.006457259	0	0
RAP1GAP	0.001661365	0.034669376	0.297451151	0.052328624	0.032434435	0	0
CDC42	0.146334825	0.025068625	0	0	0.009622801	0	0
E2F2	0.001088244	0	0	0	0.015824483	0	0
RPL11	1.169283165	0.05834764	0.175697519	0	0.392047251	1.429388	0
ELOA-AS1	0.001026624	0	0	0.006563618	0	0	0
PITHD1	0.018506381	0.251109158	0.387833818	0	0.492710148	0	0
SRSF10	0.003400632	0	0.154270992	0	0	0	0
RHD	0	0	0	0	0.012967643	0	0
MACO1	0.007327135	0.030744541	0	0	0	0	0
SH3BGRL3	0.330076359	2.327405241	1.868039505	1.214539957	0.534235758	3.903952	3.650968
NUDC	0	0.039742943	0	0	0.010098632	0	0
WDTC1	0.019771941	0.042880543	0	0	0.00802437	0	0
WASF2	0.50680412	1.014430953	1.267716037	0.352733686	0.434432998	0.647501	3.650968

EPB41	2.057832697	1.37358321	0.929399	0.277896003	0.376092811	0	2.738226
AL445235.	0.052717607	0.517941496	7.755655761	1.529561722	0.008182493	0	0
PEF1	0	0	0	0	0.002942449	0	0
PTP4A2	0.003418625	0.025068625	0	0	0.012176316	2.047502	0
YARS	0	0.040736516	0	0	0.004144683	0	0
SMIM12	0.010752376	0	0	0	0	0	0
TRAPPC3	0	0	0	0	0.003942565	0	0
THRAP3	0.035372157	0	0	0	0.004168247	0	0
STK40	0	0	0	0	0.003587418	0	0
LSM10	0.174131644	0	0	0	0.028580636	0	0
C1orf109	0	0	0	0	0.0144655	0	0
C1orf122	0.001904427	0	0	0	0	0	0
AKIRIN1	0	0	0	0	0.006892619	0	0
YBX1	4.942319288	1.788417583	2.04264708	4.041337606	2.929616889	2.714559	0
C1orf50	0.001020287	0	0	0	0	0	0
SVBP	0.008833134	0.116345959	0.119341711	0.634920635	0.026253105	0	0
ERMAP	0.025114207	0	0	0	0.13213874	0	0
SLC2A1	0.007383469	0.146859545	0.126502214	0.892857143	0.032601781	0	0
SLC2A1-AS	0.02242057	0.013692947	0	0	0	0	0
ATP6V0B	0	0	0	0	0.006994377	0	0
SLC6A9	0.007297044	0	0	0	0.004341013	0	0
AL139220.	0.069689356	0.010319941	0	0	0.076534469	0	0
RPS8	0.664955123	0.229240282	0.613314081	0.016806723	0.084689834	0	0
UROD	0.001529114	0.046556019	0	0	0.02122686	0	0
TESK2	0.161530012	0	0	0	0	0	0
PRDX1	0	0	0	0	0.008809063	0	0
UQCRH	0.184349865	0.071978055	0	0.004175278	0.014244643	1.147842	0
PDZK1IP1	1.138459301	1.337815986	2.121986407	0.820501809	0.726967884	4.942398	0
TAL1	0.032145689	0	0.154270992	0.030362836	0.007987678	0	0
CMPK1	0	0	0	0	0.003279258	0	0
RNF11	0.111205246	0.049467177	0	0	0.137013116	0	0
TTC39A	0.173349281	0	0	0	0.003942565	0	0
NRDC	0.001163037	0.13873117	0.204035829	0	0.021279795	0	0
TMEM59	0	0	0	0	0.079136846	0	0
SSBP3	0	0	0	0	0.014705118	0	0

NFIA	0.034379865	0	0.048654698	0	0.014127885	0	0
DOCK7	0.001497224	0.045262796	0.180717448	0	0.00426074	0	0
MIER1	0.003177166	0	0	0	0	0	0
SERBP1	0.033043946	0.049387573	0	0	0.0140128	0	0
GADD45A	0	0.009875519	0	0	0.009153205	0	0
CTBS	0	0	0	0	0.003071027	0	0
SH3GLB1	0.004310759	0.037033197	0	0	0.003587418	0	0
EVI5	0.002249152	0	0	0	0	0	0
RPL5	0.091338476	0.100566681	0.140558015	0	0.025479555	0	0
CCDC18	0	0.049377595	0.372387469	0	0.005985909	0	0
DNTTIP2	0.001605449	0	0	0	0	0	0
GCLM	0	0	0	0	0.004471592	0	0
TMEM56	0.049701542	0	0	0	0.009211722	0	0
AC092802.	0	0	0	0	0.002405702	0.591856	0
DBT	0	0.098755191	0	0	0	0	0
RTCA-AS1	0.044983041	0.046556019	0	0	0.004811046	0	0
RTCA	0.111051883	0.013247648	0	0	0.012216137	0	0
CLCC1	0	0	0	0	0.003568765	0	0
SARS	0	0	0	0	0.005884897	0	0
GNAI3	0	0.050920645	0	0	0	0	0
LAMTOR5	0	0.041781042	0	0	0.597610351	0	0
RAP1A	0	0	0	0	0.002405702	0	0
SLC16A1	0	0.004893275	0	0	0.004160184	0	0
CSDE1	0	0.042880543	0.083225141	0	0.041716152	0	0
MAN1A2	0.174523403	0	0	0	0.01724671	0	0
TENT5C	0.516485528	0.307033851	0.523610632	0.680272109	0.34334445	1.147842	2.231147
POLR3C	0.001840321	0.001020326	0.316255534	0	0.003208162	0	0
RBM8A	0.008175143	0	0	0.840336134	0.008006469	0	0
PPIAL4G	0.001246024	0	0	0	0	0	0
MRPS21	0.001082938	0	0.105418511	0	0.004683227	0	0
RPRD2	0	0.042880543	0	0	0	0	0
ENSA	0.001157733	0	0	0	0.008263517	0	0
GOLPH3L	0	0	0.191670021	0	0.009251335	0	0
PSMD4	0	0.038796682	0	0	0.042171418	0	0
SELENBP1	0.195733538	1.318532886	1.089500338	0.751879699	0.548342536	0	0

THEM5	0	0	0	0	0.006456772	0	0
CHTOP	0	0	0	0	0.003993818	0	0
JTB	0.100749054	0.049171078	0	0.016806723	0.06131758	0	0
RAB13	0.260941584	0	0	0	0.011907739	0	0
RPS27	2.021097448	1.520949587	1.664999372	2.870756161	0.101520169	2.16337	0
C1orf43	0.018463759	0.047781701	0	0	0.020708857	0	0
KRTCAP2	0	0	0	0	0.002942449	0	0
ASH1L	0.151219586	0	0	0	0	0	0
YY1AP1	0.027880997	0	0	0	0.004883146	0	0
LAMTOR2	0	0	0	0	0.004586672	0	0
HDGF	0.001371011	0	0	0	0.01388703	0.327955	0
AL138900.	0.012993273	0	0	0	0	0	0
SPTA1	0	0	0	0	0.008737823	0	0
ACKR1	0.180718579	0.11832988	0.143752516	0	0.040500108	0	0
DCAF8	0	0	0	0	0.127185267	0	0
ITLN1	0.009664522	0.046556019	0	0	0.018210632	0	0
UFC1	0.158225958	0.243627349	0	0	0.062517748	0	0
B4GALT3	0.003081234	0.050920645	0	0	0.052522907	0	0
NDUFS2	0	0.020890521	0	0	0.002505092	0	0
APOA2	0	0	0.191670021	0	0	0	0
UHMK1	0.01644439	0.052563247	0	0	0.004418913	0	0
PBX1	0.152424857	0.027157677	0	0	0.028168418	0	0
CREG1	0	0	0.154270992	0	0.071930503	0	0
MPC2	0.157940456	0.049377595	0	0	0.04704558	0	0
DCAF6	0.017120706	0.017906161	0	0	0.039859575	0	0
SCYL3	0	0.049377595	0	0	0	0	0
PRRC2C	0.040434519	0.115327657	0	0	0	0	0
PIGC	0.467765019	0.251294189	0.272761184	0	0.167994225	0	0
SUCO	0.020949977	0	0	0	0.004720232	0	0
PRDX6	1.706299043	2.682662005	2.732007537	2.588385523	0.89782784	4.389765	3.76506
RC3H1	0.075609793	0	0	0	0	0	0
AL121983.	0.007434436	0	0	0	0	0	0
RABGAP1L	0.019365996	0	0.150597874	0	0.010602569	0	0
GLUL	0.290012639	0.45013635	1.382357172	0.028624161	0.463641318	0	0
NPL	0.005684557	0.130596121	0.570047178	0	0.057736969	2.047502	0

SMG7	0.007985753	0.025262956	0	0	0.005632629	0	0
SWT1	0.236938395	0	0	0	0	0	0
SHISA4	0.02224636	0	0	0	0.041068416	0	0
RNPEP	0	0	0	0	0.004883146	0	0
SYT2	0.042305479	0.078078323	0	0	0	0	0
KLHL12	0	0	0	0	0.011435929	0	0
ADIPOR1	1.718334308	1.723835616	2.355475788	1.413409409	2.995606052	4.872914	0
TMEM183/	0.084508038	0.135696638	0.344160435	0	0.073839215	0	0
TMCC2	0	0	0	0	0.01880885	0	0
DYRK3	0	0	0	0	0.006556141	0	0
PFKFB2	0	0	0	0	0.004165556	0	0
YOD1	0.315918172	0.124302729	0.239317822	0	0.063628414	0	0
CR1L	0.101635165	0.050920645	0	0	0.011178727	0	0
RCOR3	0.040336096	0.002331131	0	0	0.035948283	0	0
HLX	0	0.049377595	0	0	0	0	0
AIDA	0.24869221	0.13603646	0.191551904	0.501253133	0.070524864	0	0
BROX	0.003177166	0	0	0	0	0	0
WDR26	0	0	0	0.280112045	0.019030248	0	0
CNIH3	0.002609049	0.090055193	0.186032667	0	0.015281971	0.393546	0
TMEM63A	0.0066987	0	0	0	0	0	0
PYCR2	0	0	0	0	0.010912443	0	0
H3F3A	0.006686096	0.035423058	0	0	0.055617353	0	0
ARF1	0.056237211	0.038522765	0	0	0.083028206	0	0
GUK1	3.229703657	6.867871048	4.537224693	11.33205479	4.300949604	2.8855	0
TRIM11	0	0	0	0	0.006082984	0	0
HIST3H2A	0.007027482	0.025813856	0	0	0	0	0
HIST3H2BE	0	0	0	0	0.004029739	0	0
RNF187	0.022083802	0.047925313	0	0	0.018084749	0	0
SDCCAG8	0.137671905	0	0	0	0	0	0
COX20	0	0	0	0	0.011727979	0	0
TRIM58	0.108575373	0.067200852	0	0.638091363	0.260240743	0	0
ACP1	0.094686663	0.050943794	0	0.793650794	0.118298111	0	0
RPS7	0.443518115	0.137922438	0.45877787	0	0.051116228	0	0
NRIR	0.002349527	0	0	0	0	0	0
CMPK2	0	0	0	0	0.012104256	0	0

AC017076.	0	0.029626557	0	0	0	0	0
MBOAT2	0.014300444	0	0	0	0.008675177	0	0
HPCAL1	0	0	0	0.003170728	0.016408991	0	3.650968
ODC1	0	0	0	0	0.094347288	0	0
LINC00570	0.033339215	0.101940842	0	0	0.118594908	0	0
MFSD2B	0	0	0	0	0.015602701	0	0
FKBP1B	0.029925411	0.092701688	0.154270992	0	0.028042129	0	0
FAM228B	0	0	0	0	0.004586672	0	0
POMC	0	0.050920645	0	0	0.003231623	0	0
RAB10	0.209038839	0.146771272	0	0	0.007327215	0	0
HADHA	0.001646738	0.039742943	0	0	0.076570674	0	0
SELENOI	0.001529443	0	0	0	0	0	0
OST4	0.478133182	1.067385628	1.338134836	0.035982178	0.516529184	0.94697	0
SNX17	0	0.038796682	0	0	0.011350224	0	0
NRBP1	0	0	0	0	0.011862302	0	0
AC092164.	0.005543932	0	0	0	0	0	0
PPP1CB	0	0	0	0	0.059089319	0	0
YPEL5	0	0.005990664	0	0	0.010672463	0	0
LBH	0.054538331	0.066619584	0	0	0.053010588	0	3.76506
YIPF4	0	0	0	0	0.003587418	0	0
STRN	0.000846009	0	0	0	0	0	0
CEBPZOS	0	0.136099591	0.077135496	0	0	0	0
COX7A2L	0.001226881	0.040736516	0	0	0.00426694	0	0
SIX3	0	0	0.107205266	0	0	0	0
CALM2	0.004678947	0	0	0	0.011838847	0	0
ACYP2	0.001063493	0.047925313	0	0	0	0	0
RPS27A	0.183566403	0.13331031	0.162182325	0	0.075025376	0	0
CCDC88A	0	0.023962657	0	0	0.003735302	0	0
USP34	0	0	0	0	0.007939469	0	0
CCT4	0.029128363	0.029861799	0	0	0.00566229	0	0
C1D	0.067723348	0.042880543	0	0	0.03201406	0	0
PPP3R1	0	0	0	0	0.00475432	0	0
FBXO48	0.131617047	0	0	0	0.010223679	0	0
PCBP1	0	0.016294606	0	0	0.021460957	0	0
DGUOK	0	0	0	0	0.003837505	0	0

INO80B	0.009314968	0	0	0	0	0	0
MRPL53	0.070101572	0	0	0	0.019531869	0	0
HTRA2	0.002096555	0	0	0	0.003897314	0	0
TMSB10	0.161530012	0	0	0	0	0	0
KDM3A	0	0	0	0	0.004456149	0	0
CHMP3	0.061543381	0.036210237	0	0	0.033941054	0	0
RMND5A	0	0	0	0	0.0151003	0	0
CYTOR	0.00245928	0	0	0	0	0	0
KRCC1	0.053116103	0	0	0	0	0	0
RPIA	0.327535832	0.296875905	0.191670021	0.56022409	0.238223095	0	0
ANKRD39	0.00306218	0	0	0	0	0	0
RPL31	3.843375262	0.73462153	1.072751382	0.154440154	0.387785167	1.847746	0
MAP4K4	0	0.050920645	0	0	0	0	0
FHL2	0	0	0	0	0.008095068	0	0
GCC2	0.003177166	0	0	0	0	0	0
MIR4435-2	0.002318108	0.050920645	0	0	0.004343351	0	0
ACTR3	0	0	0	0	0.004360376	0	0
PTPN4	0.003620642	0.000764287	0	0	0.0159099	0	0
CLASP1	0	0	0	0	0.002281173	0	0
GYPC	17.81933113	21.40766128	27.82093407	26.52994512	7.088722703	27.4684	20.27983
UGGT1	0	0.040736516	0	0	0	0	0
MZT2B	0.427950373	0.086911459	0	0.083664505	0.055221873	0	0
MZT2A	0.052693186	0.052381772	0.180717448	0	0.019344971	0	0
UBXN4	0.130931179	0	0	0	0	0	0
MBD5	0.044557016	0.172684193	0.131773139	0.432900433	0.01193858	0	0
KCNJ3	0	0.051795193	0.341538235	0	0	0	0
DAPL1	0.001757932	0	0	0	0.010909051	0	0
TANK	0.029439818	0	0	0	0.007650335	0	0
CYBRD1	0.001330959	0.00347433	0	0	0.008364574	0	0
SP3	0.001172438	0	0	0	0.004716091	0	0
LNPK	0.008951285	0	0	0	0	0	0
UBE2E3	0.001712195	0	0	0	0.008688968	0	0
NEUROD1	0.001569985	0.046556019	0	0	0	0	0
NUP35	0	0.040736516	0	0	0	0	0
C2orf88	0.038040925	0.084988671	0.191670021	0	0.067910233	2.228164	0

ALS2CR12	0.005636257	0	0.097309395	0	0.005651378	0	0
TRAK2	0.042664199	0.073046464	0	0	0.067577547	0	0
STRADB	0.961181007	2.361196959	3.472837032	1.524130219	1.889822951	1.037775	0
SUMO1	0	0	0	0	0.003279258	0	0
NOP58	0.025844802	0	0	0	0	0	0
NBEAL1	0.018737593	0	0	0	0	0	0
NDUFS1	0.000853835	0	0	0	0	0	0
EEF1B2	0.142254956	0	0	0	0.004202458	0	0
RPL37A	1.695506145	0.40662849	0	0	0.124624512	0	0
TNS1	0.019800782	0.416336796	0.341412225	0.921658986	0.180618665	0	0
PNKD	0.001112257	0	0	0	0	0	0
TMBIM1	0.00457228	0	0	0	0.019866285	2.367424	0
CNPPD1	0.007350828	0.072510575	0.197659709	0	0.06444911	0	0
RETREG2	0.0192775	0.034669376	0	0.004175278	0.072108114	0	0
STK16	0.004439301	0.052049063	0	0	0.011095111	0	0
TUBA4A	0.025262079	0.044039477	0	0	0	0	0
DNAJB2	0.166120392	0.237283762	0.244707672	0	0.124932025	2.164502	0
TRIP12	0.044484246	0.042880543	0	0	0.044551361	0	0
SP100	0.018208541	0	0.639598952	0	0.089115052	0	0
PSMD1	0	0	0	0	0.003837505	0	0
NCL	0	0.047925313	0	0	0.003217441	0	0
PTMA	0.326385865	0.037033197	0.197659709	0	0.017196836	0	0
GIGYF2	0	0.013247648	0	0	0	0	0
UBE2F	0.458336898	0.116625063	0.496409247	0	0.102959214	0	0
NDUFA10	0	0	0	0	0.015324496	0	0
MTERF4	0.003827313	0	0	0	0.004456149	0	0
HDLBP	0.029259919	0.086763489	0.170948938	0	0.016875273	0	0
TADA3	0.00186446	0.001493548	0	0	0.007697721	0	0
ARPC4	0	0	0	0	0.006207422	0	0
RPUSD3	0	0	0	0	0.003083061	0	0
EMC3	3.275297645	1.147611439	0.366750116	0.940434979	0.435785296	0	0
BRK1	0.004789768	0	0.107205266	0	0.043052248	0	0
RPL32	6.926344797	0.68282321	2.807494786	0.435817078	0.665248046	7.970072	0
IQSEC1	0.030158297	0	0	0	0.004954395	0	0
PLCL2	0.023355719	0.045262796	0	0	0.004837017	0	0

RAB5A	0.014046977	0.026928729	0	0	0.023755298	0	0
KAT2B	0.053101437	0	0	0	0.00799949	0	0
RPL15	0.786368895	0.201462993	0.407009765	1.638162206	0.083747583	0	0
LRRFIP2	0.009228735	0.081072674	0	0	0.025380264	0	0
GOLGA4	0.033367702	0	0	0	0.004517936	0	0
OXSR1	0.131617047	0	0.175697519	0	0.012190493	0	0
ACVR2B	1.476021687	0.078030579	71.53874645	19.57931231	0.060487866	13.00774	1.525088
SLC25A38	0.009091983	0	0.186032667	0	0.012843094	0	0
RPSA	0.060644925	0.114917258	0	0	0.018041102	0	0
EIF1B	0.10379462	0.275729139	0	0	0.250148004	0	0
RPL14	0.087322461	0.016798563	0	0	0.054686496	0	0
TRAK1	0	0	0	0	0.003279258	0	0
ZDHHC3	0.017679736	0	0	0	0.023815604	0	0
LIMD1	0	0.122843653	1.414226505	0.892857143	0	0	0
AC098613.	0.024101506	0	0	0	0.007745789	0	0
CCRL2	0.022915358	0	0	0	0	0	0
LRRC2	0.007950185	0	0	0	0	0	0
KLHL18	0.020110541	0	0	0	0.003607074	0	0
TMA7	0.239328656	0.130938802	0.197659709	0.865800866	0.120984208	0	3.442341
IP6K2	0	0.030175197	0	0	0	0	0
ARIH2	0	0	0	0	0.015324496	0	0
NDUFAF3	0.00781499	0	0	0	0.026978694	0	0
MST1	0	0	0	0	0.004811046	0	0
RNF123	0.003593636	0.143186262	0.321275464	0	0.059300582	0	0
IFRD2	0	0.037894434	0	0	0.010836153	0	0
TUSC2	0.002694208	0	0	0	0.022073695	0	0
RPL29	0.678507767	0.454335715	1.040941574	0.506553312	0.157205022	0	0
BAP1	0	0	0	0	0.004065801	0	0
PBRM1	0.002464397	0	0	0	0.013856557	0	0
TKT	0.005296309	0	0	0	0	0	0
CACNA1D	0	0.016459198	0	0	0	0	0
SELENOK	0.388736755	0.298159175	0.158127767	0.019175455	0.288404692	0	0
CCDC66	0.151219586	0	0	0	0	0	0
ARF4	0.007128707	0	0	0	0.0144655	0	0
ARF4-AS1	0.006686096	0.018105118	0	0	0.00766322	0	0

SLC25A26	0	0	0.09583501	0	0.007110696	0	0
TMF1	0.01186531	0	0	0	0	0	0
LMOD3	0.161819509	0.440550472	0.790653291	0.714285714	0	0	0
FOXP1	0.438300857	1.039995574	2.08269997	0.533781204	0.004738259	0	0
PPP4R2	0.018654385	0.004750614	0	0	0.020776134	0	0
CGGBP1	0.003550632	0	0	0	0.036445396	0	0
NSUN3	0.169627408	0.121903178	0	0	0.08226598	0	0
MTRNR2L1	0.010927682	0.00601277	0	0	0.048019829	0	0
CPOX	0	0	0	0	0.012801105	0	0
NIT2	0	0.040736516	0	0	0.005072023	0	0
PCNP	0	0	0	0	0.005072023	0	0
RPL24	0.216364652	0.030997031	0.110966854	0	0.095425485	0	0
ATG3	0	0	0.186032667	0	0.006562856	0	0
COX17	0	0	0	0	0.0144655	0	0
NDUFB4	0	0	0	0	0.060088945	0	0
ABTB1	0.07659686	0.304976692	0.191670021	0.003170728	0.249184048	0	0
CNBP	0.006680623	0.046556019	0	0	0	0	0
IFT122	0.001144128	0	0	0	0	0	0
RAB6B	0.001665648	0.047925313	0	0	0.00426694	0	0
ATP1B3	0.004336376	0	0	0	0.002611355	0	0
TFDP2	0.037259502	0.047925313	0.313306765	0	0.003231623	0	6.013358
PAQR9	0.003511522	0.018729433	0	0	0.017724211	0	0
SIAH2	0.048349119	0	0.175697519	0.005273427	0.010675589	0	0
RSRC1	0.105771349	0	0	0	0.033717695	0	0
KPNA4	0.003817036	0	0	0	0	0	0
PDCD10	0.075609793	0.056715489	0	0	0.033770459	0	0
SEC62	1.699769772	1.095839274	0.522322441	0.040159445	1.333927693	0.220868	0.971628
NCEH1	0	0	0	0	0.003482088	0	0
MFN1	0	0.044039477	0	0	0	0	0
DCUN1D1	0.007489014	0.070688449	0	0	0.070715229	0	0
KLHL24	0	0	0	0	0.007092802	0	0
AP2M1	0.237134249	1.153303991	0.2671814	0.004175278	0.314941686	0.99681	0
PSMD2	0	0	0	0	0.026786171	0	0
EIF4G1	0	0	0	0	0.012250138	0	0
IGF2BP2	0.148536042	0.106534217	0	0	0.074252962	0	0

OPA1	0.037014848	0.047925313	0	0	0	0	0
TNK2-AS1	0	0	0	0	0.002387894	0	0
BDH1	1.306766799	0.542470177	82.13510707	17.51265504	0.047704363	18.76506	10.51938
RPL35A	6.327719287	0.852012329	1.114003342	1.352835689	0.489826058	2.172457	0
ATP5ME	0.007660074	0	0	0	0.004811405	0	0
TACC3	0.001371011	0	0	0	0	0	0
MXD4	0.004654575	0.000764287	0	0	0.004519202	0	0
ADD1	0	0	0	0	0.017479571	0	0
LRPAP1	0	0	0	0	0.003115517	0	0
STX18	0	0	0	0	0.004360376	0	0
MSX1	0	0.189894684	0.227203367	0	0.058438523	0	0
PPP2R2C	0	0.045262796	0	0	0	0	0
MRFAP1	0.008245417	0.031950209	0	0	0	0	0
KIAA0232	0.033968917	0	0	0	0.005632629	0	0
FAM184B	0.125256606	0.045262796	0	0	0.005651378	0	0
SEPSECS-A	0	0	0	0	0.007096399	0	0
AC106047.	0.002378621	0.039742943	0	0	0.014479678	0	0
KLF3	0.072888809	0.051770897	0	0	0.045827296	0.50844	0
RPL9	0.513763909	0.066179934	0	0.035984167	0.03856803	0	0
OCIAD1	0.047382137	0.022019738	0	0	0.003010271	0	0
DCUN1D4	0.008440998	0	0	0	0	0	0
EXOC1	0	0	0	0	0.00589727	0	0
SRP72	0	0	0	0	0.009773696	0	0
REST	0.134100388	0	0	0	0.006540565	0	0
POLR2B	0	0	0	0	0.002281173	0	0
YTHDC1	0.002744139	0	0	0	0.012392649	0	0
DCK	0.006742122	0	0	0	0	0	0
G3BP2	0.014387289	0	0	0	0	0	0
CCNI	1.867678175	0.743688616	1.081915167	1.796825967	1.047824633	1.485443	0
LINC01094	0.001026624	0	0	0	0	0	0
BMP2K	0	0	0	0	0.005499159	0	0
HSD17B11	0	0	0	0	0.005940869	0	0
ABCG2	0.002169512	0	0	0	0.006641094	0	0
SNCA	1.614279959	1.309519459	1.309800821	1.383196767	2.753696629	0	0
TSPAN5	0.128370718	0	0	0	0.085372859	0	0

AC114811.	0	0	0	0	0.004569131	0	0
UBE2D3	0.175669904	0.6809449	1.237637263	0	0.357803205	0	0
CISD2	0.074257856	0.016294606	0	0	0.222852529	0	0
SLC9B2	0.018707647	0	0	0	0	0	0
RPL34	0.794248229	0.179752154	0	0.865800866	0.169954561	0	0
ELOVL6	0	0	0	0	0.0144655	0	0
SNHG8	0.127770273	0.042880543	0	0	0.012184034	0	0
C4orf3	0	0	0	0	0.003279258	0	0
AC108866.	0	0	0.039531942	0	0	0	0
LARP1B	0.049701542	0	0	0	0	0	0
SCLT1	0.027441392	0.025068625	0.166450281	0	0	0	0
GYPE	0	0.011893873	0	0	0.00589727	0	0
GYPB	0.157952969	0.175444536	0.170948938	0	0.076584874	0	3.650968
GYPA	0.003048399	0.04810411	0	0	0.011064919	0	0
RPS3A	0.823366818	0.148201663	0.204035829	0	0.118634853	0	0
FBXW7	0.082136258	0	0	0	0	0	0
RNF175	0	0	0	0	0.006866677	0	0
MSMO1	0	0	0	0	0.002405702	0	0
CLCN3	0.179062413	0.044039477	0	0	0	0	0
MFAP3L	0.012969563	0	0	0	0.003248546	0	0
HMGB2	0	0	0	0	0.002281173	0	0
GPM6A	0.977365903	0.103321273	56.46300652	7.232007666	0.034520596	11.32902	1.54464
IRF2	0.041201858	0.025864455	0	0	0.004854487	0	0
CFAP97	0.003879542	0	0	0	0	0	0
EXOC3	0	0	0	0	0.003532421	0	0
MRPL36	0	0	0	0	0.004165556	0	0
CMBL	0	0	0	0	0.004144683	0	0
6-Mar	0.078641214	0	0	0	0.016811851	0	0
DAP	0	0	0	0	0.016747876	0	0
ANKH	0.00771696	0.087130882	0	0	0.018623616	0	0
MYO10	0.01496278	0.088174278	0.480517836	0.56022409	0	0	0
C5orf22	0	0.005060437	0	0	0.005367398	0	0
NIPBL	0.01324815	0	0	0	0	0	0
RPL37	1.425731053	0.487485464	0.359842034	0.272108844	0.104552839	0	0
DHX29	0.004984096	0.149271115	0.137502406	0	0.008763709	0	0

GPBP1	0	0	0	0	0.003587418	0	0
TRIM23	0	0.025068625	0.219545905	0	0	0.190346	0
ERBIN	0	0	0	0	0.003587418	0	0
TAF9	0	0	0	0	0.004214537	0	0
RAD17	0.00794114	0.022631398	0	0	0	0	0
BTF3	5.266225517	0.006994687	0.466915476	38.28122978	1.020949214	0	0
NSA2	0.00125927	0	0	0	0	0	0
TENT2	0	0	0	0	0.005367398	0	0
RPS23	0.204872141	0.029097512	0	0	0.015747732	0	0
COX7C	0.112700325	0.038796682	0.124021778	0.892857143	0.073582435	0	0
RASA1	0	0	0.197659709	0	0	0	0
SLF1	0.145047358	0	0	0	0	0	0
RFESD	0	0	0	0	0.003279258	0	0
LINC02062	0.001246024	0.038796682	0	0	0.003868337	0	0
SLC25A46	0	0	0	0	0.003115517	0	0
EPB41L4A-	0	0	0	0	0.006835844	0	0
SRP19	0.02242057	0	0	0	0	0	0
REEP5	0	0	0	0	0.002405702	0	0
DCP2	0.116474523	0	0	0	0.013720103	0	0
SRFBP1	0.182937604	0	0	0	0	0	0
PRDM6	0.079678677	0.003519353	21.73053814	0	0.018979449	1.942502	0
3-Mar	0	0	0	0	0.031162393	0	0
ACSL6	0	0	0	0	0.010601393	0	0
AC116366.	0.001998122	0	0	0	0	0	0
C5orf15	0	0	0	0	0.0144655	0	0
SKP1	0.074009542	0.109052984	0.150597874	0	0.170756696	0	0
UBE2B	0.582763247	0.136622725	0.140558015	0	0.112602837	0.376903	3.256268
CAMLG	0	0.014169223	0	0	0.004217843	0	0
CXCL14	0	0.074198655	0	0	0	0	0
FAM53C	0	0	0	0	0.003821593	0	0
PAIP2	0.102217436	0.02295015	0	0	0.0727925	0	0
UBE2D2	0.010239633	0.029097512	0	0	0.068554881	0	0
CYSTM1	0.063956155	0.036210237	0.24867563	0	0.066960923	0	3.256268
NDUFA2	0.017636839	0	0	0	0.053156237	0	0
ZMAT2	0.139754828	0.175665454	0.191670021	0.426439232	0.128146914	0	0

RNF14	0	0	0	0	0.00964127	0	0
NDFIP1	0	0	0	0	0.011409701	0	0
LARS	0	0	0	0	0.005939618	0	0
CSNK1A1	0	0	0	0	0.005367398	0	0
CD74	0.323060025	0	0	0	0	0	0
RPS14	3.682483577	1.012980505	1.359229474	0.675431463	0.763525504	3.551136	0
TNIP1	0.205092585	0.028944367	0.320310093	0	0.186742969	0	0
SAP30L-AS	0.003883782	0	0	0	0	0	0
FAXDC2	0.127581568	0.023278009	0	0.529100529	0.092962338	1.99362	0
AC008609.	0.214544991	0.30410517	0.324364651	0.432900433	0	1.761804	0
SLU7	0	0.004893275	0	0	0.004160184	0	0
PTTG1	0.033844384	0	0	0	0.002505092	0	0
MAT2B	0	0.022321379	0	0	0.002281173	0	0
CPEB4	0.019660476	0	0	0	0.027016664	0	0
THOC3	0.002899764	0	0	0	0	0	0
AC139491.	0.003418625	0	0	0	0	0	0
CLTB	0.021058345	0	0	0	0.118594769	0	0
TSPAN17	0	0	0	0	0.003071027	0	0
UIMC1	0.094369164	0	0	0	0.004998925	0	0
NSD1	0	0	0	0	0.006994377	0	0
PDLIM7	0.00142832	0.063367914	0	0.607902736	0.015175864	0	0
B4GALT7	0.308902715	0.158715516	0.5040802	0	0.042681513	0	0
SQSTM1	0.003992467	0	0	0	0.012392981	0	0
CNOT6	0	0	0	0	0.003892611	0	0
MGAT1	0	0	0	0	0.003304114	0	0
TRIM41	0	0.038796682	0	0.492610837	0	0	0
RACK1	0.467967671	0.293454393	0.279681765	0	0.158707751	0	0
MYLK4	0.002061886	0	0	0	0.002901949	0	0
TUBB2A	0	0	0	0	0.009037299	0	0
CDYL	0.226283088	0.100298241	0.329076705	0	0.023395374	0	0
TMEM14B	0.181204447	0.043205396	0	0	0.058756337	0	0
GMPR	0.081499735	0.037033197	0	0	0.553694242	0	0
AL009031.	0.004170963	0	0	0	0.002636755	0	0
ALDH5A1	0.00590309	0	0	0	0	0	0
C6orf62	0.002759279	0	0	0	0.053282719	0	0

HIST1H1C	0	0	0	0	0.008305372	0	0
HIST1H4C	0.00239465	0	0	0	0	0	0
ZSCAN16-A	0	0.011475075	0	0	0.013387957	0	0
HLA-A	0.01817729	0	0	0	0.040322209	0	0
ZNRD1	0	0	0	0	0.005652368	0	0
PPP1R11	0.00710022	0	0	0	0.00458081	0	0
TRIM10	0.012801375	0.037033197	0	0	0.020856317	0	0
BAG6	0.030153069	0.064014525	0	0	0.193222476	0	0
CSNK2B	0	0	0	0	0.002971998	0	0
CLIC1	0	0	0	0	0.013164405	0	0
C6orf48	0.071663986	0	0	0	0.004029739	0	0
NELFE	0.155175403	0.042880543	0	0	0.017132052	0	0
RPS18	0.087755977	0.142407985	0	0.004175278	0.116531909	0	0
RPS10	2.313970903	0.41016185	0.154270992	0.811212175	0.360341213	0	0
TAF11	0	0	0	0	0.007643186	0	0
LHFPL5	0.016376315	0.341066852	1.539350869	0	0	0.102792	0
PIM1	0.093804386	0.401143183	0.626268912	0	0.304404234	0	0
CCDC167	0	0	0	0	0.004861803	0	0
ZFAND3	0.006426149	0	0	0	0.006039705	0	0
TSPO2	0.032513178	0.019170125	0	0	0	0	0
TREML2	0	0.046556019	0.209286751	0	0	0	0
UBR2	0.005682602	0	0	0	0	0	0
BICRAL	0	0	0	0	0.005652368	0	0
MEA1	0	0	0	0	0.003685372	0	0
YIPF3	0.006821735	0.130934088	0	0.154440154	0.035693476	0	0
TMEM63B	0.026840533	0.037894434	0	0.016806723	0.134929489	0	0
HSP90AB1	0.001998122	0.094960122	0	0.003170728	0.013659352	0.591856	0
CD2AP	0	0	0	0	0.015926504	0	0
FBXO9	0.411935009	0.741239421	0.906825905	0.876108019	0.2982652	2.643961	0
GCLC	0.001009419	0	0	0	0.008051347	0	0
KIAA1586	0.025469616	0	0	0	0	0	0
ZNF451	0.000846009	0.039742943	0.197659709	0	0	0	0
KCNQ5	0.576518325	0	18.91028506	1.755092276	0.05055276	4.460186	0
EEF1A1	0.478865708	0.165051773	0.197659709	0	0.156552792	0	0
COX7A2	0.045882926	0.015518673	0	0	0.005939618	0	0

PNRC1	0.221121143	0.050920645	0.310274342	0	0.110354891	0	3.543586
SNX3	0.304598793	0.351257587	0.313699522	0.344234079	0.458903069	2.047502	0
FOXO3	0.372579976	0.078669854	0	0	0.091324157	0	0
SLC22A16	0.001108959	0	0	0	0.004341013	0	0
AC002464.	0	0	0	0	0.008689696	0	0
TSPYL1	0	0	0	0	0.004341013	0	0
RWDD1	0	0	0	0	0.016104532	0	0
ARG1	0	0	0	0	0.011675125	0	0
RPS12	54.49936624	11.15654245	11.28690188	13.96874495	4.495104915	21.88418	15.04605
TBPL1	0.002361236	0.000853121	0	0	0.011593274	0	0
CCDC28A	0	0	0	0	0.002405702	0	0
SF3B5	0.115259038	0	0	0	0	0	0
EPM2A	0	0	0	0	0.004181756	0	0
SOD2	0.132105251	0	0	0	0.024182704	0	0
TCP1	0	0.018729433	0	0	0.0110271	0	0
SFT2D1	0.002370687	0	0	0	0	0	0
AC093627.	0	0.039742943	0	0	0	0	0
GPR146	0.11807084	0.049377595	0.180717448	0.19436346	0.178729389	0	7.207401
AC073957.	0	0.045262796	0	0	0	0	0
MICALL2	0.016226759	0	0	0	0.003279258	0	0
PSMG3	0.003418625	0	0	0	0.008293221	0	0
NUDT1	0	0.050920645	0	0	0.013020908	0	0
CHST12	0	0	0.197659709	0	0	0	0
IQCE	0	0	0	0	0.00426694	0	0
GNA12	0	0.021440272	0	0	0.011005257	0	0
WIPI2	0	0	0	0	0.022762988	0	0
ACTB	0.217399846	0.303291027	0.711876395	0	0.331880395	0	0
EIF2AK1	0.151177058	0.294395899	0.577337513	0	0.295698606	0	0
RAC1	0.002808108	0	0	0	0	0	0
KDELR2	0	0	0	0	0.031645204	0	0
NDUFA4	0.249118128	0.100298241	0	0	0.009167505	0	0
ARL4A	0.168800003	0.059354575	0	0	0.068341206	0	0
SNX13	0.064612005	0	0	0	0.003786602	0	0
TOMM7	0.219319683	0	0	0	0	1.13071	0
TAX1BP1	0.135501415	0.096232402	0.175697519	0	0.053840242	0	0

JAZF1	0.692412048	0.407338996	0.363599848	0	0.371194462	0	0
JAZF1-AS1	0	0.037894434	0	0	0.012713966	0	0
MTURN	0.067050194	0.084800653	0	0	0.012614269	0	0
NT5C3A	0	0	0	0	0.018362813	0	0
HERPUD2	0.021994477	0.05466353	0	0	0.021961934	0	0
7-Sep	0.161530012	0	0	0	0.006062406	0	0
STK17A	0.088248704	0.049377595	0	0	0.014713917	0	0
DBNL	0	0	0	0	0.003279258	0	0
CHCHD2	0.00515381	0.054675163	0	0.055157198	0.007093374	0	0
TMEM248	0.00245673	0	0	0	0.008163505	0	0
GTF2I	0	0	0	0	0.007786378	0	0
MDH2	0.038625158	0.050920645	0	0	0.003479459	0	0
HSPB1	0.226398695	0.275114297	0.504068781	0.035984167	0.172034656	0.102792	0
SEMA3E	0	0.216136631	0.107205266	0.064495324	0	0	0
AKAP9	0.004439301	0.085352701	0.296759149	0	0.008142311	0	0
SAMD9	0.031605965	0.206759393	0	0	0.01305644	0	0
PDAP1	0.004209792	0.056796291	0	0	0.037154985	0	0
BUD31	0	0	0	0	0.004499033	0	0
ATP5MF	0.027748047	0	0	0	0.037347943	0	0
ZNF394	0	0	0	0	0.004510987	0	0
ZNF655	0	0	0	0	0.006487741	0	0
TAF6	0	0	0.197659709	0	0.005462839	0	0
LAMTOR4	0	0	0	0.079365079	0.003786602	0	0
TSC22D4	0.024764183	0	0	0	0.013603642	0	0
TFR2	0	0	0	0	0.003685372	0	0
GNB2	0.008234542	0.039742943	0	0	0.022534399	0	0
POP7	0.0025934	0	0	0	0.019318716	0	0
ACHE	0	0.051449294	0	0	0.008229062	0	0
ZNHIT1	0.043532148	0	0.204035829	0	0.010636352	0	0
FIS1	0.603581446	0.3474678	0	0.875989205	0.367509693	0.190346	0
POLR2J	0	0	0	0	0.043668181	0	0
UPK3BL1	0	0	0	0	0.004569131	0	0
DNAJC2	0	0.050920645	0	0	0	2.295684	0
KMT2E	0.033057305	0.050920645	0	0	0.003343498	0	0
GPR85	0	0	0.186032667	0	0	0	0

ARF5	0	0	0	0	0.003209119	0	0
SND1	0	0	0	0	0.016204199	0	0
ATP6V1F	0.008632384	0.041781042	0.180717448	0	0.020688012	0	0
UBE2H	0.094084148	0.016459198	0	0	0.055733896	0	0
BPGM	0.105014035	0.232677734	0	0.221483942	0.590953445	0	0
STMP1	0.641356437	0.761759089	0.303164347	0.655914267	0.364053184	0	0
MTPN	0.157943889	0.092521781	0.180717448	0	0.025774306	0	0
KDM7A	0	0.052563247	0	0	0.021003785	0	0
MKRN1	1.221048596	1.54423779	0.670474849	0.019175455	1.975863788	0	0
NDUFB2	0	0	0	0.013502565	0	0	0
BRAF	0	0	0	0	0.003587418	0	0
KEL	0	0	0	0	0.021645763	0	0
GSTK1	0.05835795	0.123389015	0	0	0.016603246	0	0
CASP2	0.002532901	0	0	0	0	0	0
FASTK	0.001191903	0	0	0	0.004683227	0	0
NUB1	0.002405999	0.029097512	0	0	0.003587418	0	0
RBM33	0	0.047925313	0	0	0.003279258	0	0
DNAJB6	0	0	0	0	0.008595274	0	0
SLC25A6	0.111645862	0.080479459	0.22470788	0	0.047286025	0	0
CD99	0.009972854	0	0	0	0.008362526	0	0
XG	0.001082938	0	0	0	0	0	0
HCCS	0	0.050920645	0.162182325	0	0.0144655	0	0
TMSB4X	0.161530012	0	0	0	0	0	0
GLRA2	0.01813092	0.361520367	1.233054958	0	0.007407233	0	0
SYAP1	0	0	0	0	0.017245667	0	0
TAB3	0.131617047	0.044039477	0	0	0	0	0
XK	0.0016882	0.042880543	0	0	0.03942398	0	0
DDX3X	0	0.029792276	0	0	0.003217441	0	0
CDK16	0.001199548	0	0.091668271	0	0	0	0
UXT	0.035447579	0	0	0	0	0	0
SLC38A5	0.193919384	0.007306998	0	0.004175278	0.16874956	0	0
TBC1D25	0.006455332	0	0	0	0	0	0
WDR13	0.030279962	0.038796682	0.089086066	0.523655798	0.03335513	0	1.853568
GATA1	0.148239513	0.123376899	0.112948405	0	0.03901198	0	0
PCSK1N	0.008475975	0.013140812	0	0	0.031245459	0	0

TIMM17B	0	0	0	0	0.012025404	0	0
PQBP1	0.001019117	0	0	0	0	0	0
TFE3	0	0	0	0	0.002611355	0	0
WDR45	0.055060003	0.348548935	0.279029176	0	0.147095818	0	0
GPKOW	0.015585253	0	0	0	0.004226686	0	0
HUWE1	0.005425436	0	0	0	0	0	0
ALAS2	2.286258932	7.943543836	5.851919249	6.86666489	5.421271028	3.299883	5.519615
PAGE2B	0.433368862	0.009935736	0.129083892	0.005273427	0.04164019	0	0
PAGE2	0.004461595	0	0	0	0	0	0
FAM104B	0.014297922	0	0	0	0.009254368	0	0
YIPF6	0.034887686	0.09544379	0	0	0.01926792	0	0
SNX12	0	0	0	0	0.006280776	0	0
FOXO4	0.003177166	0.136262374	0.319296453	0	0.044317078	0	0
RPS4X	0.532427511	0.132099101	0.297334263	0	0.149427623	0.323659	0
ATRX	0.00504423	0	0	0	0	0	0
COX7B	0.029998481	0	0	0	0.005591622	0	0
CYSLTR1	0	0	0.191670021	0	0	0	0
LPAR4	0	0.038796682	0	0	0	0	0
HMGN5	0	0.028587029	0	0	0.002505092	0	0
SH3BGRL	0.00995423	0.095933614	0.170948938	0	0.034440879	0	0
RPL36A	1.39813057	0.2207599	0.158127767	0	0.170596012	0	0
SLC25A53	0	0.049377595	0	0	0.008816664	0	0
RPL39	1.811008435	0.183888499	0	0.405275842	0.186168127	0	0
STAG2	0.232145192	0.891956763	2.738909579	1.974836757	0.008708733	0	0
BCORL1	0.126916438	0	0	0	0	0	0
MBNL3	0.162285092	0.298696784	0.494410362	0.865800866	0.054134482	0	0
FAM122B	0	0	0	0	0.003583831	0	0
MOSPD1	0.152175734	0	0	0	0	0	0
RTL8C	0	0	0	0	0.007885129	0	0
INTS6L	0.001397428	0	0	0	0	0	0
IDS	0.00170113	0	0	0	0.00372409	0	0
GABRE	0.221538359	0	37.50742084	1.586152414	0.011854491	6.051109	0
CETN2	0	0.097302909	0	0	0.012293172	0	0
SLC6A8	0.011150048	0	0.26736579	0	0.046105129	0	0
IDH3G	0.002196329	0.039742943	0	0	0.00868925	0	0

SSR4	0.011622387	0	0	0	0.006161472	0	0
NAA10	0.003809778	0.029097512	0	0	0.003090445	0	0
RPL10	0.846715661	0.198569356	0.124021778	0	0.043508172	0	0
G6PD	0	0.046556019	0	0	0.015350713	0	0
MPP1	0.164119051	0.656699229	0.561677871	0.39900546	0.77234719	1.721763	0
FUNDC2	0.090251338	0.040892595	0.312534881	0.539083558	0.114842913	0	0
VBP1	0	0	0	0	0.01306315	0	0
CLIC2	0	0.040736516	0	0	0.009307001	0	0
VAMP7	0	0	0	0	0.0144655	0	0
AC100810.	0.061578429	0.231591733	0.338217724	0.079365079	0.079242934	0	0
MFHAS1	0.00453273	0	0	0	0.002597477	0	0
NEIL2	0.003177166	0	0	0	0.006402405	0	0
FDFT1	0.182078598	0.050920645	0.158127767	0	0.066849367	0	0
CTSB	0.012712669	0.277341457	0.541142846	0	0.221713876	0	0
ZDHHC2	0.005710421	0.094960122	0	0	0.006489251	0	0
XPO7	0.009556365	0.150232959	0	0	0.183657178	0	0
DMTN	0.668667162	0.747330553	1.59270444	0.82401363	0.925044817	0	3.886514
REEP4	0	0	0	0	0.004360376	0	0
R3HCC1	0	0	0	0	0.006911949	0	0
AC104561.	0	0	0	0	0.020735884	0	0
SLC25A37	48.8467528	10.73040691	9.871750673	11.27901417	13.37293982	11.26451	9.935713
BNIP3L	5.378512151	0.233586147	3.3167999	2.36591614	1.623891868	5.729933	247.8076
PTK2B	0.002320379	0.003711755	0.186032667	0	0.010090065	0	0
SARAF	0.002686062	0.05176144	0	0	0.021405475	0	0
LSM1	0.003871571	0.063573654	0	0	0.044435097	0	0
NSD3	0.012490897	0.045891137	0.306672033	0	0.014593345	0	0
GOLGA7	0	0	0	0	0.0144655	0	0
ANK1	0.035225113	0	0	0	0.028026706	0	0
VDAC3	0	0.023615372	0	0	0.0144655	0	0
PCMTD1	0.202617901	0.103361682	0.180717448	0	0.10841949	0	0
RB1CC1	0.197425571	0.036210237	0	0.274725275	0.005639522	0	0
TCEA1	0.433996531	0	0.137502406	0	0.01689745	0	0
RPS20	1.615958873	0.151787251	0.356981605	0	0.116899918	1.13071	0
SDCBP	0.001183961	0	0	0	0.004160184	0	0
ARMC1	0	0.034669376	0	0	0.004341013	0	0

CSPP1	0.022110513	0	0	0	0	0	0
TRAM1	0	0	0	0	0.006076302	0	0
RPL7	0.893174211	0.519320634	0.140558015	0.352733686	0.508977758	2.164502	0
C8orf59	0.010985039	0	0	0	0	0	0
CA1	0.651090265	0.837463495	0.345045426	0	0.69036289	0	0
CA3-AS1	0.002305326	0	0	0	0.03862397	0	0
AC100801.	0.009982192	0.047925313	0	0	0.018531301	0	0
C8orf88	0	0	0	0	0.00426694	0	0
RUNX1T1	0	0.047925313	0	0	0	0	3.886514
UQCRB	1.04660663	0.068878168	0.332228036	0.005273427	0.177285802	1.847746	0
MTDH	0	0	0	0	0.005499159	0	0
RPL30	5.7938007	2.243725259	3.063685616	0.408729403	0.57002806	8.243061	0
COX6C	0	0.039742943	0	0	0.0144655	0	0
RNF19A	0.134100388	0	0	0	0.012776116	0	0
PABPC1	0.293398235	0.10660526	0.147095597	0	0.009839626	0	0
NCALD	0.109395231	0.341232783	0.589737021	0.303951368	0	0	0
EIF3H	0.126392996	0	0	0	0.020892598	0	0
RAD21	0.103004646	0	0.158127767	0	0	0	0
RNF139	0.004960408	0	0	0	0	0	0
NDUFB9	0	0	0	0	0.007174836	0	0
ASAP1	0	0.076598577	0	0	0.003568765	0	0
NDRG1	0	0	0	0	0.005072023	0	0
ST3GAL1	0.029439574	0	0	0	0.056750166	0	0
ZFAT	0.008178735	0	0	0	0	0	0
EEF1D	0.314909089	0.162021899	0	0.013127236	0.059543382	0	0
TSTA3	0.16293126	0.349676451	0	0	0.167383408	0	0
GRINA	0.015163767	0.114526284	0.034375602	0	0.113143242	0	0
GPAA1	0	0	0	0	0.007723518	0	0
SHARPIN	0.228808615	0.170765111	0.38932973	1.169220664	0.205421577	0	3.543586
MAF1	0.157405417	0.39833217	0	0	0.42571711	1.306165	0
VPS28	0.178834698	0.23285717	0.197659709	0.664451827	0.136152539	0	0
RPL8	0.76663739	0.421829631	0.4670473	0.131693703	0.204526625	0.923873	0
WASHC1	0.002824849	0	0	0	0.014225505	0	0
RCL1	0	0	0	0	0.005651378	0	0
CNTLN	0.011632276	0	0	0	0	0	0

RRAGA	0	0	0	0	0.031797919	0	0
RPS6	1.121014073	0.204875423	0.166450281	0	0.128051259	0	0
SMIM27	0	0	0	0	0.004775789	0	0
BAG1	3.147059187	1.247025329	1.790679961	1.508795129	1.860805201	2.483247	0
CHMP5	0.004278941	0.020116798	0	0	0.003942565	0	0
UBE2R2	0	0	0	0	0.008020683	0	0
UBAP2	0.084737981	0	0.137502406	0	0.015656625	0	0
DCAF12	1.313027786	0.920557641	1.103712624	0.494275378	1.598508256	1.645374	0
UBAP1	0.030652189	0.041781042	0	0	0.031423892	0	0
STOML2	0.065170242	0	0	0	0.032037155	0	2.231147
FAM214B	0	0	0	0	0.151656153	0	0
EBLN3P	0.003403889	0	0	0	0.00700653	0	0
DCAF10	0	0.032589213	0.158127767	0	0.016144894	0	0
SLC25A51	0.050962166	0	0	0	0.018689493	0	0
PIP5K1B	0.009996231	0.035423058	0.020602966	0	0.003279258	0	0
ZFAND5	0	0.069745854	0	0	0.031817807	0	0
C9orf40	0.038211401	0	0	0	0	0	0
NMRK1	0.002361236	0	0	0	0.007421151	0	0
UBQLN1	0	0.041781042	0	0	0.019250199	0	0
AGTPBP1	0.011408219	0	0	0	0	0	0
ISCA1	0.104132395	0.232054757	0.150597874	1.308791618	0.364450609	0	2.077275
CKS2	0.134100388	0.017906161	0	0	0.0144655	0	0
SECISBP2	0.12514801	0	0.129083892	0	0.060486492	0	0
AUH	0	0	0	0	0.003892611	0	0
FAM120AC	0.00160183	0.030744541	0.186032667	0	0.005842794	0	0
TMOD1	0.011751453	0	0	0	0.078098289	0	0
HEMGN	0.244764994	0.001264128	0.083225141	0	0.474901355	211.8077	0
ANP32B	0.066746938	0.268549789	0.520299958	0.751879699	0.440134857	0.670421	0
NANS	0.002249152	0.037033197	0	0	0.00384987	0	0
ERP44	0.00139141	0.026281623	0.162182325	0	0	0	0
RNF20	0.000944118	0.036210237	0	0	0.002611355	0	0
RAD23B	0	0	0	0	0.008057497	0	0
CTNNAL1	0.022263326	0.004893275	0	0	0.025368102	0	0
TXN	0	0	0	0	0.028756469	0	0
HSDL2	0.064612005	0	0	0	0	0	0

CDC26	0.001110519	0	0	0	0	0	0
FBXW2	0	0.028587029	0	0	0.004811046	0	0
STOM	0	0	0	0	0.015334474	0	0
AL359644.	0.002058303	0	0	0	0	0	0
PSMB7	0.006629055	0.050920645	0.147095597	0.079365079	0.023053471	0	0
RPL35	0.099475151	0.013247648	0	0	0.01838751	0	1.853568
PPP6C	0	0	0	0	0.02394507	0	0
MAPKAP1	0.209038839	0	0.191670021	0	0.00769411	0	0
RPL12	7.406107086	2.197169706	1.746314303	3.985149934	0.682972641	2.6012	8.061658
AK1	0.106693624	0	0	0	0.17562372	0	0
ST6GALNA	0.209678039	0.409578012	0.331940642	0	0.218507889	0	3.256268
PIP5KL1	0	0	0	0	0.005732729	0	0
DPM2	0.044650881	0.429101252	0.645647584	1.369047619	0.31295154	0.122585	0
C9orf16	0	0.002483934	0	0	0.002636755	0	0
CIZ1	0.007784023	0	0	0	0	0	0
ZER1	0.0085941	0	0	0	0.026182915	0	0
LRRC8A	0.001108959	0	0	0	0.004834842	0	0
SH3GLB2	0	0.020890521	0	0	0.013496287	0	0
CRAT	0.026808135	0	0	0	0.024309373	0	0
ASB6	0	0	0	0	0.004789616	0	0
TOR1A	0	0	0	0	0.017754729	0	0
C9orf78	0.642512208	0.65985134	0.9488652	1.296392496	0.960410499	0.378788	0
USP20	0.005180263	0.009585063	0	0	0.014132588	0	0
GFI1B	0.002370417	0.062689292	0	0	0.00641907	0	0
GBGT1	0.046144173	0	0	0	0.056777049	0	0
RPL7A	0.405934826	0.253393641	0.117131679	1.124934458	0.074958829	1.305888	0
RXRA	0.011845534	0	0	0	0	0	0
QSOX2	0	0	0	0	0.010522345	0	0
SNHG7	0.161530012	0	0	0	0	0	0
EDF1	0.046123631	0.082263481	0.316255534	0	0.00823505	0	0
SSNA1	0.000946129	0	0	0	0.017504179	0	0
TUBB4B	0.010447104	0.084395421	0.170948938	0	0.018425791	0	0
TALDO1	0.14387199	0.488054534	0.34716773	0	0.422312987	0	0
RPLP2	8.61062258	1.265279809	1.447833473	1.718137603	0.993718266	3.99422	0
PNPLA2	0.029957715	0.287248216	0	0.56022409	0.095691333	0	0

POLR2L	0.002318108	0.034669376	0	0	0.08799971	0	0
CHID1	0.001009419	0	0	0	0.047814965	0	0
TOLLIP	0.04276905	0.022799512	0	0	0.022353325	0	0
MOB2	0.017435861	0	0	0	0.021978175	0	0
CTSD	0.002218265	0	0	0	0.022701824	0	0
MRPL23	0	0	0	0	0.002971998	0	0
C11orf21	0.001539714	0	0	0	0.004217843	0	0
TSSC4	0	0	0	0	0.012058297	0	0
NAP1L4	0.042279083	0	0	0	0.019260752	0	0
NUP98	0.045559747	0	0	0	0.002505092	0	0
HBB	4382.814471	6612.590778	6074.30383	6159.71513	5026.870444	5846.683	5991.053
HBD	0.938271196	1.843173153	1.455225345	0.344234079	4.529817496	0.327955	0.696427
HBG1	0.090148288	0.044039477	0	0.462246826	0.190127155	0	0
AC104389.	0.058496465	0	0	0.087085651	0.053774808	0	0
HBG2	1.275898501	0.001473292	1.971956613	226.0260748	12.2223398	9.303195	0
AC104389.	0	0.037894434	0	0.004175278	0.007735747	0	0
TPP1	0.169221918	0	0	0	0	0	0
RPL27A	1.56917632	0.160396821	0.191670021	0	0.191603039	3.310128	0
TMEM9B	0.135917997	0.032589213	0	0.174216028	0.03022047	0	0
ZNF143	0.002739908	0	0	0	0	0	0
SBF2-AS1	0.005150477	0	0	0	0.004811046	0	0
MTRNR2L8	0.051241347	0	0	0	0.013927203	0	0
USP47	0.011979211	0	0	0	0	0	0
MICAL2	0.017130914	0.019632056	0	0	0.063550348	0	0
PDE3B	0.002644092	0	0	0	0	0	0
C11orf58	0.316995524	0.002417597	0	0	0.069203472	0	0
RPS13	2.266992251	0.549793096	0.553447185	0.432900433	0.30534858	1.761804	0
NUCB2	0	0	0	0.004175278	0	0	0
PAX6	0	0.322685936	0.175697519	0	0.002597477	0	0
AL035078.	0.00604364	0.142067857	0.356414967	0	0	0	0
CD59	0	0	0	0	0.011073291	0	0
CAT	0	0	0.175697519	0	0.162896941	0	0
CD44	0.018365169	0	0	0	0.009674033	0	0
AC021713.	0	0	0	0.414078675	0.002971998	0	0
PHF21A	0	0	0	0	0.008998065	0	0

PSMC3	0	0	0	0	0.002942449	0	0
SLC43A1	0	0	0	0	0.003735302	0	0
UBE2L6	0.00426354	0	0	0	0.014426726	0	0
YPEL4	0.001360253	0	0	0	0.132482053	0	0
VWCE	0.075972501	0.052563247	0	0.621118012	0.039595955	0	0.696427
DDB1	0	0	0	0	0.017946664	0	0
TKFC	0	0	0	0	0.003587418	0	0
RAB3IL1	0	0.011556459	0.263546279	0	0.008117411	0	0
FTH1	3.272945147	4.149465929	4.910499571	5.271207486	2.173801266	11.25477	4.619318
EEF1G	0.019580486	0	0	0	0.015598855	0	0
UBXN1	0.044914881	0.030744541	0	0.336401192	0.036410385	1.646904	0
STX5	0.004336376	0	0	0	0	0	0
DNAJC4	0.027979179	0	0	0	0.020941722	0	0
BAD	0.038626742	0	0	0	0	0	0
PRDX5	0.195442223	0.210634727	0	0.736776011	0.259110949	0	0
PPP2R5B	0.003922694	0.091640846	0.162182325	0	0.034177807	0	0
VPS51	0	0.046556019	0	0	0.020380548	0	0
FAU	1.62270151	0.342544607	0.28932798	0	0.321620666	0	0
CAPN1	0	0	0	0	0.014161391	0	0
MALAT1	0.273578067	0.065285451	0.143752516	0	0.379162098	1.721763	0
SCYL1	0.001924017	0	0	0	0	0	0
DRAP1	0.001242104	0.014293514	0	0	0.014727674	0	0
BANF1	0	0	0	0	0.004068878	0	0
CCS	0	0.067837092	0	0	0.034770657	0	0
POLD4	0.128247784	0.078538622	0	0	0.043295656	0	0
RPS6KB2	0.002061886	0.036210237	0.166450281	0	0.019713775	0	0
GSTP1	0.006642356	0	0	0	0	0	0
GAL	0	0.045262796	0	0.680272109	0.004181756	0	0
AP002387.	0	0	0	0	0.004029739	0	0
LAMTOR1	0	0	0.191670021	0	0.018545249	0	0
CLPB	0.00764228	0	0	0	0.003587418	0	0
AP005019.	0.002971288	0	0	0	0	0	0
ARAP1	0	0.028094149	0	0	0	0	0
RAB6A	0.043872349	0.019632056	0	0	0.018450927	0	0
UCP2	0.208725732	0.390969656	0.485746734	0.732600733	0.075268751	0	0

C2CD3	0	0	0	0	0.003993818	0	0
PGM2L1	0.040370849	0	0	0	0.017282579	0	0
RPS3	0.421679862	0.302797885	0.097309395	0.858146118	0.133416411	0	0
THAP12	0.057783094	0	0	0	0	0	0
CAPN5	0.006577841	0	0	0	0.008139682	0	0
AAMDC	0	0	0.191670021	0	0.004861803	0	0
TAF1D	0	0.031335782	0	0	0.0144655	0	0
SRSF8	0.005790237	0	0	0	0.047427501	0	0
SESN3	0.166165733	0.206112852	0.254669575	0.272108844	0.25088337	0.626096	0
BIRC2	0.268536735	0.23336712	0	0.634920635	0.076118322	0	0
DCUN1D5	0	0	0	0	0.003279258	0	0
NKAPD1	0	0	0	0	0.004217843	0	0
TIMM8B	0	0	0	0	0.003587418	0	0
REXO2	0.017107976	0	0.121636744	0	0.07707155	0	0
SIK3	0	0	0	0	0.003587418	0	0
TMPRSS13	0	0	0	0	0.004168247	0	0
ATP5MG	0.128031578	0.055770231	0	0	0.116961158	0	0
DDX6	0.003099573	0	0.162182325	0	0.004499033	0	0
RPS25	2.187208488	0.579458896	0.549326095	0.179144385	0.423793192	4.734848	0
HMBS	0.003475609	0.021440272	0	0	0.028930462	0	0
RNF26	0.001108959	0	0	0	0	0	0
ARHGEF12	0.017775994	0.073325729	0.166450281	1.367053999	0.016088863	0	0
TBCEL	0.229585812	0	0	0	0.029480756	0	0
HSPA8	0.060746329	0.089120538	0	0	0.021972613	0	0
ZBTB44	0.033272902	0.040736516	0	0	0.005256325	0	0
AP002986.	0.024678196	0	0	0	0	0	0
GDI2	0.016226759	0.049377595	0	0	0	0	0
FBH1	0	0	0	0	0.004789616	0	0
UPF2	0.095245883	0.000840795	0	0	0.023886787	0	0
OPTN	0.542736792	0.850853184	0.722545295	0.352232809	0.652717196	0	0
FRMD4A	0.001388149	0.020626084	0	0	0	0	0
RPP38	0	0	0	0	0.004360376	0	0
RSU1	0.002058303	0.117683269	0	0	0.025175866	0	0
PIP4K2A	0.187024971	0.100444424	0.03880436	0.865800866	0.174161177	0	0
YME1L1	0.003463607	0	0	0	0	0	0

KIF5B	0	0	0	0	0.003115517	0	0
8-Mar	0.340711852	0.215714323	0.421008073	0	0.12923862	0	0
WASHC2C	0	0	0	0	0.006676597	0	0
NCOA4	0.297309222	0.153354501	0.301880283	0	0.174344393	0	0
HK1	0	0	0.175697519	0.224971879	0.097224219	0	0
SAR1A	0	0	0	0	0.002281173	0	0
EIF4EBP2	0.066354746	0.028587029	0	0	0.038569165	0	0
ANAPC16	0.001998122	0.084450452	0.315219705	0	0.033493113	0	0
ECD	0.001122445	0	0	0	0	0	0
MRPS16	0.00235732	0	0.150597874	0	0.003786602	0	0
RPS24	4.15113763	0.765101175	0.964609167	0	0.288177987	0.642013	3.650968
NUTM2B-A	0.001097656	0	0	0	0	0	0
GHITM	0	0	0	0	0.022926673	0	0
WAPL	0.005288185	0	0	0	0.011340852	0	0
NUTM2A-A	0.023941403	0.086721995	0	0	0.150073136	0	0
IFIT1B	0.372830704	0.07662869	0	0	0.072424655	0	0
KIF20B	0.002872805	0.212115651	0	0	0	0	0
PCGF5	0.85069718	1.006936049	1.188956853	3.568142304	0.779319078	1.183712	3.650968
PLCE1	0.126916438	0.119928569	0.294362404	0.368446692	0	0	0
HPS1	0.271705099	0.757063493	0.418166359	0.772200772	0.205886397	0.205584	0
DPCD	0.016207921	0.041756842	0	0.019175455	0.026228582	0	0
POLL	0.009321021	0.07863095	0.170948938	0	0.030487672	0	0
FBXW4	0	0	0	0	0.013049944	0	0
OGA	0	0	0	0	0.019862545	0	0
ACTR1A	0	0	0	0	0.010778852	0.658762	0
ATP5MD	0	0	0	0	0.03567248	0	0
SLK	0.026716683	0	0	0	0	0	0
MXI1	0.286143442	0.237119186	0	0	0.139304561	0	0
SHOC2	0.351195098	0.206292468	0.162182325	0	0.007747842	0	0
VTI1A	0.001246024	0.013692947	0	0	0	0	0
CCDC186	0.044221671	0	0	0	0	0	0
FAM204A	0	0.036210237	0	0	0.035197232	0	0
RGS10	1.043172535	0.831117933	0.566268356	0.820501809	0.727628532	1.389304	0
TIAL1	0.015654891	0.036210237	0	0	0.025434865	0	0
IKZF5	0	0	0	0	0.003942565	0	0

OAT	0.001163037	0.042880543	0	0	0.006866677	0	0
LHPP	0.005429143	0.047925313	0.170948938	0	0	0	0
UROS	0.002349527	0.070487483	0	0	0.048880658	0	0
FOXI2	0	0.037033197	0.575362397	0	0	0	0
GLRX3	0	0.088364066	0	0	0.008775858	0	0
ZNF511	0	0	0	0	0.004181756	0	0
NINJ2	0.008728537	0.56760714	0.473613758	0	0.081102606	2.228164	0
WNK1	0.22100684	0.501932618	0.297451151	0.639095913	0.081698075	0	0
GAPDH	0.161530012	0.047925313	0	0	0	0	0
MLF2	0	0	0	0	0.007047453	0	0
PTMS	0.137518641	0	0	0	0.038704141	0	0
FOXJ2	0.001529443	0	0	0	0	0	0
AC092745.	0.001109652	0	0	0	0.002611355	0	0
AC092490.	2.246765578	3.615990998	2.912550368	4.864567452	1.278124793	5.588178	3.08928
GABARAPL	0.001574157	0.040736516	0	0	0	0	0
YBX3	0.439973292	0.532629182	0.908499792	0.200968143	0.655267973	0.41855	0
CREBL2	0	0.020626084	0	0	0.003587418	0	0
CDKN1B	0.024092612	0	0	0	0	0	0
HEBP1	0.057399019	0.049377595	0	0	0.089385884	0	3.543586
H2AFJ	0.003861903	0.037033197	0	0.003170728	0.035970679	0	0
STRAP	0.031422802	0	0	0	0.046113826	0	0
CMAS	0	0	0	0	0.004341013	0	0
ST8SIA1	0	0	0	0	0.008263723	0	0
ITPR2	0	0	0	0	0.004834842	0	0
FGFR1OP2	0.19447117	0.235753361	0	0.634920635	0.293043721	2.228164	0
CCDC91	0	0.038565481	0.137502406	0	0.008034387	0	0
KIAA1551	0.089274141	0	0	0	0.004937711	0	3.256268
ZCRB1	0.003327397	0	0	0	0	0	0
AC008035.	0	0	0	0	0.003821593	0	0
SLC48A1	0.009156916	0.015518673	0	0	0.032915882	0	0
METTL7A	0	0	0	0	0.002505092	0	0
DAZAP2	0.004779637	0.092440556	0	0	0	0	0
KRT1	0.030985432	0.345575432	0.479403231	0.166512941	0.267089786	0	3.012048
EIF4B	0.028316018	0	0.186032667	0	0.007939469	0	0
PFDN5	4.493073085	1.670897199	0.946945528	0.829562594	0.735466682	0.814598	0

C12orf10	0.002464397	0.049377595	0	1.068907563	0.05820016	0	0
PCBP2	0.948391254	0.288269708	0.36736754	0.003170728	0.246751137	0.41855	0
HNRNPA1	0	0.020626084	0	0	0.141360526	0	0
NFE2	0.088617895	0.407757354	0.478410919	0	0.301359649	0.670421	0
BLOC1S1	0.002530196	0.082082461	0.067288412	0	0.044028503	0	0
PYM1	0	0.027157677	0	0	0.006476223	0	0
RAB5B	0	0	0	0	0.00806744	0	0
RPS26	0.558211031	0.264794137	0.329076705	0	0.028050122	0	0
PA2G4	0.065133576	0.203933878	0.32344316	0	0.091620397	1.683502	0
RPL41	17.79051774	3.484005782	2.471405464	1.494566676	1.776048631	1.221896	4.318349
MYL6B	0	0	0	0	0.004569131	0	0
MYL6	0.259399264	0.46622256	0.358120302	0.22465467	0.493382003	0	0
ATP5F1B	0.042651127	0.121568173	0.575362397	0.005273427	0.065258459	0	0
PTGES3	0.088365301	0	0.170425807	0	0.092219457	0	0
NACA	1.28872134	1.046891866	0.527637273	1.059021624	0.529866057	1.262626	0
MBD6	0	0.029097512	0	0	0	0	0
CTDSP2	0.005218297	0	0	0	0.014016647	0	0
USP15	0.61500427	0.250117986	0.058028538	0.680272109	0.062589712	0.376903	0
HMGA2	0.002532901	0.033947097	0	0	0	0	0
CPSF6	0.002055327	0	0	0	0	0	0
CCT2	0.135911501	0	0.109053633	0	0	0	0
MGAT4C	0	0.031335782	0	0	0	0	0
POC1B	0.031036334	0	0	0	0.020459414	0	0
POC1B-AS1	0	0	0	0	0.003279258	0	0
BTG1	0	0	0	0	0.008777284	0	0
NUDT4	0.001273257	0.049377595	0	0	0.059515647	0	0
VEZT	0.126766774	0.033254299	0	0	0	0	0
CHPT1	0.021065358	0	0	0	0.022745427	0	0
CHST11	0.004830441	0	0	0.30075188	0.033099729	0	0
TCP11L2	0.020633684	0.157390104	0.086645352	0	0.029350272	0	0
ISCU	0.027279673	0.104633476	0	0	0.067914095	0	0
ARPC3	0	0	0	0	0.002405702	0	0
VPS29	0	0	0	0	0.002405702	0	0
HECTD4	0	0	0	0	0.003279258	0	0
RPL6	0.30895082	0.331931655	0.186032667	0.634920635	0.111933897	1.183712	0

TPCN1	0	0	0	0	0.003543285	0	0
MED13L	0	0.033254299	0	0	0	0	0
TESC	0.238324244	0.604018137	1.036728984	0.030362836	0.493874557	1.306165	0
SUDS3	0.082708429	0.040736516	0	0.638265571	0.075215914	0	0
RPLP0	0.029059639	0	0	0	0.067760979	0	0
COX6A1	0.147489736	0	0	0	0.045860384	0	0
GATC	0	0.023449028	0	0	0.003837505	0	0
DYNLL1	0.024764183	0.046556019	0	0	0.031593681	0	0
RNF10	0.432948155	0.748080572	0.658636291	0.607902736	1.348757536	0	0
PSMD9	0.048349119	0	0	0	0.01938289	0	0
BCL7A	0	0.064929998	0.257803139	0	0	0	0
ARL6IP4	0	0.018516598	0	0	0.007268604	0	0
EIF2B1	0	0	0	0	0.002942449	0	0
UBC	0.062006146	0.347918176	0	0	0.030553092	0	0
PSPC1	0	0.036210237	0	0	0.003217441	0	0
SAP18	0.006047697	0.002925423	0	0	0.012749545	0	0
USP12	0.1900959	0	0	0.228571429	0.015183202	0	0
RPL21	39.07407739	6.878735322	7.413751748	7.270837136	3.87909086	15.87699	11.53722
GTF3A	0.011490318	0.038735229	0	0	0.043572939	0	0
POLR1D	0.290335584	0.094634329	0	0	0.30279551	0	0
N4BP2L2	0.041178328	0.082836159	0.09583501	0	0	0	0
WBP4	0	0.00185166	0	0	0	0	0
RGCC	0.001970971	0	0	0	0.05377717	0	0
TPT1	11.39986242	4.752405121	5.714575532	5.995304294	1.784592096	6.99369	2.749092
MED4	0	0	0	0	0.0144655	0	0
ITM2B	0.066448157	0.106094086	0.143752516	0	0.056628814	0	0
INTS6-AS1	0	0	0	0	0.004883146	0	0
AL359513.	0	0	0	0	0.002405702	0	0
SUGT1	0.00306218	0	0	0	0.013977817	0	0
RAP2A	0.004294453	0	0	0	0	0	0
CUL4A	0.004678947	0.025460323	0	0	0.069217455	0	0
TFDP1	0.217588976	0	0	0	0.022861741	0	0
CDC16	0	0.136622725	0	0.178571429	0.015749723	0	0
UPF3A	0	0	0	0	0.004854487	0	0
PNP	0.005146503	0	0	0	0.0144655	0	0

RAB2B	0.145211098	0.050920645	0	0.030362836	0.032800703	0	0
TOX4	0	0	0	0	0.007683218	0	0
LRP10	0.003463607	0	0	0	0.08907039	0	0
C14orf119	0	0	0	0	0.0144655	0	0
DCAF11	0.010418801	0	0	0.751879699	0.018642395	0	0
PSME1	0.001331707	0.050920645	0.124021778	0	0.011692384	0	0
EMC9	0.01083433	0	0	0	0.011017099	0	1.323977
CHMP4A	0.001009419	0	0	0	0	0	0
NEDD8	0	0	0	0	0.01873244	0	0
KHNYN	0	0.025460323	0	0.192385487	0.007632615	1.646904	0
STRN3	0.02292684	0	0.170948938	0	0	0	0
C14orf28	0	0	0	0	0.004065801	0	0
RPS29	1.099469632	0.090415046	0.292829199	0.472559353	0.086087981	0	3.543586
RPL36AL	0.439657511	0.069156132	0	0	0.115643034	0	0
KLHDC2	0.005087559	0	0	0	0.018738354	0	0
NEMF	0.013720696	0	0	0	0	0	0
ATP5S	0.009108772	0	0	0	0.003892611	0	0
CDKL1	0	0.035423058	0.09583501	0	0.01345485	0	0
MAP4K5	0.046880926	0	0	0	0.006251256	0	0
TXNDC16	0.010261097	0	0	0	0.005652368	0	0
LGALS3	0.447621515	0.844508521	0.904354611	1.983515462	1.146522815	2.30115	0
ATG14	0	0	0	0	0.005042458	0	0
KTN1	0.002227302	0.073466058	0	0	0.005639522	0	0
PPM1A	0.069187855	0.17591584	0	0	0.313435602	0	0
SPTB	0	0	0	0	0.00904085	0	0
CHURC1	0.007312033	0	0	0	0	0	0
MAX	0.010814902	0	0.175697519	0.041832253	0.022864282	0	0
ATP6V1D	0.001199548	0	0	0	0	0	0
PLEK2	0.089720365	0.136947249	0.343863574	0	0.079322394	2.228164	0
VTI1B	0.262587558	0.157812068	0.34030366	0	0.157174834	0	0
SRSF5	0.087744698	0	0	0	0	0	0
ENTPD5	0.159293844	0.094640391	0	0	0.012803207	0	0
BBOF1	0.368468739	0.570876456	0.309277923	0	0.145550682	1.803752	0
LTBP2	0.004461595	0	0	0	0.003564033	0	0
DLST	0	0.031335782	0	0	0.003735302	0	0

AHSA1	0.002899764	0	0	0	0	0	0
KCNK10	0	0.104540208	0.197659709	0.064495324	0.003774639	0	0
FOXN3	0.008159955	0	0	0	0	0	0
CALM1	0.029490957	0	0	0	0	0	0
NDUFB1	0.002896219	0.015087599	0	0	0.006198773	0	0
IFI27	0.044878151	0.037894434	0	0.005273427	0.069009846	0	0
SNHG10	0.00359137	0	0	0	0.012279397	0	0
GLRX5	0.009325499	0.087130882	0.158127767	0	0.097833659	0	0
LINC02318	0.016582245	0	0	0	0	0	0
VRK1	0.017126074	0	0	0	0	0	0
SETD3	0.00197316	0	0	0	0	0	0
YY1	0	0.144108754	0.361730186	0	0.016162938	0	0
CINP	0	0	0	0	0.003685372	0	0
EIF5	0.005547677	0.085999312	0.272385719	0	0.030773456	0	0
MARK3	0.043004483	0.170201068	0	0.478767856	0.094066194	0.220868	0
BAG5	0.002644092	0	0	0	0	0	0
APOPT1	0.001665648	0.001153192	0.175697519	0	0.019972013	0	0
ZFYVE21	0	0	0	0	0.003821593	0	0
ATP5MPL	0.071253253	0.049377595	0	0.004175278	0.043217702	0	0
LINC02280	0	0.044039477	0	0	0	0	0
LINC02352	0.045439805	0.132994655	0.277417136	0.56022409	0.089812993	0	0
AC012236.	0	0	0	0	0.00458081	0	0
KLF13	0	0	0	0	0.017190549	0	0
GREM1	0.177683014	0.452250235	0.654909312	0	0	0	0
SLC12A6	0	0	0	0	0.004214537	0	0
SRP14	0	0	0	0	0.010742197	0	0
CCDC32	0	0.191957996	0.75798987	0	0	0	0
CHP1	0.032602524	0	0	0	0.006753392	0	0
NUSAP1	0.12922401	0.022631398	0.138177068	0	0	0	0
CCNDBP1	0.016385819	0.313145568	0	0.710973563	0.101653514	0	0
EPB42	0.133574946	0.69714001	0.612987742	0.082338411	0.340257255	0	0
LCMT2	0	0	0.137502406	0	0	0	0
SERF2	8.312617034	2.594456501	2.579186875	1.754128265	3.713891235	3.693771	5.319023
EIF3J	0	0	0	0.030362836	0.008569509	0	0
BLOC1S6	0.009100937	0.09045768	0.158127767	0	0	0	0

COPS2	0.072523679	0	0	0	0.003730981	0	0
CCPG1	0.124767311	0.090595496	0	0	0.005235849	0	0
ADAM10	0.111657164	0.338660351	0.985220515	0.064495324	0	0	0
SLTM	0	0	0	0	0.00854347	0	0
TPM1	0.026000483	0	0	0	0.059621913	0	0
HERC1	0.006781794	0	0	0	0	0	0
FAM96A	0.007870787	0	0	0	0.003587418	0	0
OAZ2	0.045289541	0.188755619	0.140558015	0.680272109	0.067473535	0	0
MTFMT	0	0	0	0	0.002281173	0	0
RPL4	0.597979107	0.263151757	0.191670021	0	0.119437866	0	0
ITGA11	0	0.045262796	0.117131679	0	0	0	0
RPLP1	3.951225308	2.240408229	1.454682435	0.378772193	0.549880836	0	0
TMEM202·	0	0	0	0	0.005499159	0	0
SEMA7A	0.040382503	0	0	0	0	0	0
UBL7	0.048245468	0.193519779	0.543390415	0.004175278	0.119292694	0	3.012048
CLK3	0.002442378	0.046556019	0.129083892	0	0.040067711	0.196773	0
AC100835.	0.025013766	0.055603003	0	0	0.078563232	0	0
ETFA	0.001605449	0	0	0	0	0	0
DNAJA4	0.103137194	0	0.058028538	0	0.014781934	0	0
PSMA4	0.031036334	0	0	0	0.003115517	0	0
MORF4L1	0.033498194	0.152940605	0.197659709	0	0.026929486	0	0
FAH	0.004870396	0	0	0	0	0	0
RPS17	2.368326133	0.088337061	0	0	0.263026107	0	0
WHAMM	0.21537335	0	0	0	0	0	0
ZSCAN2	0.038417949	0	0	0	0	0	0
AKAP13	0.139359226	0	0	0	0	0	0
ISG20	0	0.006466114	0	0	0.022431402	0	0
AC091167.	0	0.000764287	0	0	0	0	0
FURIN	0.001331707	0.018308547	0	0	0.042770565	0	0
MAN2A2	0	0	0	0	0.002611355	0	0
UNC45A	0.001020287	0	0	0	0.032918031	0	0
CHD2	0.035359804	0.046556019	0	0	0.059395971	0	0
NPRL3	0.374808378	0.436175516	0.481913195	0.082338411	0.320461697	0	3.012048
HBZ	0.264570996	0.033254299	0	0	0.089194563	0	0
HBM	6.929647453	2.861061265	3.393689024	3.611740061	3.965571955	0.658762	1.25502

HBA2	3740.501851	2098.011564	2216.507667	2193.497673	3104.668648	2385.3	2301.518
HBA1	1240.354466	877.5683202	900.6366516	905.9240651	1623.764788	1006.926	1000.462
HBQ1	1.186405525	0.536466802	0.860455482	0.835976416	0.688967543	0	0
NME4	0.061239461	0.081827233	0	0	0.046580669	0	0
STUB1	0	0	0	0	0.008270645	0	0
UBE2I	0.004183238	0	0	0	0	0	0
SPSB3	0.005492672	0.066588536	0.276367449	0	0.063825243	0	0
HAGH	0.555361606	0.919400663	1.102820137	0.858082117	0.559234945	1.147842	0
NDUFB10	0.014713847	0	0	0	0.018469276	0	0
RPS2	0.74876272	0.513941894	0.483532707	0.004175278	0.340623401	0	0
TRAF7	0	0	0	0	0.011028152	0	0
ATP6V0C	0.004857375	0	0	0	0.020882547	0	0
ELOB	0.485922468	2.384036013	1.089860282	2.253185965	0.825394537	0.378788	0
HCFC1R1	0.017671136	0	0	0	0	0	0
NAA60	0.024764183	0	0	0	0.012352236	0	0
UBALD1	0.007200933	0.049377595	0	0	0.051592144	0	0
ROGDI	0.063162963	0.147068178	0.345786987	0	0.044871651	0	3.650968
GLYR1	0.000846009	0.047925313	0	0	0	0	0
METTL22	0.040439403	0.02671247	0	0	0.173487363	0	0
CARHSP1	0.052988018	0.049321099	0	0.004175278	0.029752577	0	0
USP7	0	0	0	0	0.0144655	0	0
AC022167.	0.129607017	0.156586029	0	0	0.04883525	0	0
GSPT1	0.236031463	0.405436943	0.150597874	0.577030812	0.582891386	0	0
SNX29	0.007688828	0	0.021886196	0	0.010280212	0	0
NTAN1	0.034378546	0.050920645	0	0	0.013917727	0	0
MARF1	0.453092942	0.426337992	0.143752516	0	0.103062212	0	0
FOPNL	0.006642356	0	0	0	0	0	0
RPS15A	9.909475803	0.738079836	1.615006198	2.249716178	0.795453129	0	3.442341
ARL6IP1	1.151121199	0.179043271	0.134576823	0.214555596	0.081431636	1.99362	2.738226
SMG1	0.013186123	0	0	0	0.003587418	0	0
AC130456.	4.527900643	1.393814141	2.388025051	1.02553607	0.437605889	3.841049	3.543586
GDE1	0.004833959	0.07863095	0.129083892	0	0.12021388	0	0
ACSM3	0	0	0	0	0.004660223	0	0
METTL9	0.002157002	0.024688798	0	0	0.04987895	0	0
UQCRC2	0	0	0	0	0.003304114	0	0

POLR3E	0	0	0	0	0.014185604	0	0
NPIPB5	0.007327135	0.00085716	0	0	0	0	0
UBFD1	0.002793758	0	0	0	0.00426694	0	0
CDIPT	0.242434361	0.128653312	0	0	0.060241777	0	0
YPEL3	0.02434268	0.627576335	0.364062487	1.332734352	0.261098082	0	0
BOLA2B	0.004950043	0	0	0	0.011452612	0	0
1-Sep	0.001766672	0.037894434	0	0	0.062448851	0	0
AC093249.	0.015874067	0	0	0	0	0	0
RNF40	0.000849244	0	0	0	0	0	0
STX4	0	0	0	0	0.003942565	0	0
C16orf58	0	0	0	0	0.003821593	0	0
AHSP	1.423213255	2.200163642	2.249464189	0.687473538	2.713803339	2.164502	3.442341
DNAJA2	0	0.042880543	0	0	0.08402699	0	0
AMFR	0.003819969	0	0	0	0.009813832	0	0
MT2A	0.258178967	0.163481821	0.206363028	0	0.183008936	0	0
MT1A	0.002381012	0	0	0	0	0	0
ARL2BP	0	0	0	0	0.004217843	0	0
KIAA0895L	0	0.23422909	0.838114692	0.793650794	0	0	0
E2F4	0	0	0	0	0.016250829	0	0
GFOD2	0	0	0	0	0.033379796	0	0
RANBP10	0	0.008621485	0	0	0.020608177	0	0
THAP11	0.001963348	0	0	0	0.009566449	0	0
NUTF2	0	0	0	0	0.004217843	0	0
VPS4A	0	0.029626557	0	0	0.019463959	0	0
COG4	0	0.023615372	0	0	0.007092802	0	0
ATXN1L	0	0	0	0	0.042509615	0	0
TXNL4B	0.00142832	0	0	0	0	0	0
CFDP1	0	0	0	0	0.0094675	0	0
GABARAPL	0.209059545	0.034857555	0.559389896	0	0.160221334	0	0
KARS	0.001319837	0	0	0	0	0	0
TERF2IP	0.036610209	0.047925313	0.143752516	0	0.132352764	0	0
COX4I1	0.002671925	0	0	0.793650794	0.010836017	0	0
MAP1LC3E	0.130375274	0.008576109	0.170948938	0	0.046863565	0	0
SLC7A5	0	0	0	0	0.018666393	0	0
ACSF3	0	0	0	0	0.003543285	0	0

RPL13	2.766259728	0.949052347	1.013241403	0	0.421383964	0	0
CHMP1A	0	0	0	0	0.005648889	0	0
GLOD4	0.161530012	0	0	0	0	0	0
YWHAE	0	0	0	0	0.008305372	0	0
INPP5K	0	0	0	0	0.100391705	0	0
PITPNA	0	0	0	0	0.003279258	0	0
RILP	0.013064964	0.153235518	0.137502406	0	0.103546281	0	0
MIR22HG	0	0	0	0	0.008595274	0	0
WDR81	0	0	0	0	0.003217441	0	0
RPA1	0	0	0	0	0.002405702	0	0
UBE2G1	0	0	0	0	0.003942565	0	0
ARRB2	0.080951283	0.243491579	0.552782783	0	0.060192271	0	0
RNF167	0	0	0	0	0.004168247	0	0
PFN1	0	0	0	0	0.004444484	0	0
CAMTA2	0	0	0	0	0.002405702	0	0
C1QBP	0.014714711	0	0	0	0.025256338	0	0
RNASEK	0.004779637	0	0	0	0	0	0
ACADVL	0.002150475	0.037033197	0	0	0.012816334	0	0
GABARAP	0.458569871	0.050920645	0	0	0.302074433	0	0
EIF5A	0.028804091	0.190244482	0	0	0.048290229	0	0
GPS2	0.003620642	0	0.121636744	0	0.028621864	0	0
RPL26	1.238580302	0.098553008	0	0	0.066257499	0	0
NCOR1	0.033844384	0	0	0	0	0	0
UBB	4.061573639	9.134554717	6.331988126	11.38697637	7.623041567	13.29724	0
LRRC75A	0.008351728	0	0	0	0.003231623	0	0
FLCN	0.008244738	0.041781042	0.180717448	0	0.019889536	0	0
COPS3	0.097080479	0.011807686	0	0	0.070085319	0	0
GID4	0.001144128	0	0	0	0.033114473	0	0
ALKBH5	0	0	0	0	0.030242701	0	0
FLII	0	0	0	0	0.004068878	0	0
PRPSAP2	0.002396265	0	0	0	0.008595274	0	0
SPECC1	0.135224936	0.126098034	0.29503923	0	0.018089537	0	0
TMEM11	0	0	0	0	0.003543285	0	0
MAP2K3	0.334349057	0.550215622	0.93907656	0.483536482	0.705720829	1.37741	0
MTRNR2L1	0.006425067	0	0	0	0.003343498	0	0

IFT20	0	0	0	0	0.027580907	0	0
PIGS	0	0	0	0	0.007769769	0	0
RPL23A	3.211956521	0.835768518	1.074098448	0.947681704	0.209876542	0	0
ERAL1	0.017348706	0	0	0	0.008299031	0	0
AC024267.	0.046680457	0	0	0	0.006154087	0	0
AP2B1	0.398977551	0.221995262	1.08076999	0	0.07927487	0	0
SYNRG	0	0.002582347	0	0	0	0	0
RPL19	0.656715041	0.599781846	0.651545817	1.483696163	0.1473719	0	3.76506
CDK12	0.007267199	0.038796682	0	0.446428571	0.02120167	0	0
NEUROD2	0	0.0767851	0	0	0	0	0
MIEN1	0.008075752	0.021161827	0.3421478	0	0.014960471	0	0
ORMDL3	0	0.012830399	0.137502406	0	0.047142514	0	0
PSMD3	0	0	0	0	0.007049791	0	0
CASC3	0.045346571	0.258220633	1.498784486	0	0.051875266	0	0
TNS4	0.044700129	0.343489063	1.861457564	0	0	0	0
EIF1	2.002521545	2.373440835	1.92341238	2.534955824	1.483974341	0	0
TTC25	0.008157636	0	0	0	0	0	0
NKIRAS2	0	0	0	0	0.003115517	0	0
RAB5C	0.004560197	0.237843046	0.102017914	0	0.106069491	0	0
RETREG3	0	0	0	0	0.004517936	0	0
BECN1	0.019157198	0	0	0	0	0	0
RPL27	0.181288358	0	0	0	0.023907706	0	0
LSM12	0.001183961	0	0	0	0	0	0
TMUB2	0.002567674	0	0	0	0.011379825	0	0
SLC4A1	0.213269231	0.553250439	0.828538744	0	0.50686412	0	0
RUNDC3A	0.081213441	0.060635066	0	0	0.085546543	0	0
SLC25A39	7.285891073	22.63636151	22.56162729	22.13965298	9.217742257	13.2872	21.45329
DCAKD	0	0.049377595	0.191670021	0	0	0	0
HEXIM1	0	0.020368258	0	0	0.01957783	0	0
PLEKHM1	0	0	0	0	0.004341013	0	0
KANSL1-AS	0.02113803	0.079261095	0	0	0.037604493	0	0
CDC27	0.013993381	0.014293514	0.27153253	0.549450549	0.149102521	0.190346	0
MYL4	2.325470438	1.220314696	1.060010572	0.197225471	0.388705708	2.541406	0
HOXB2	0	0	0	0	0.003872052	0	0
CALCOCO2	0.009524986	0.050920645	0	0	0.016604796	0	0

SNF8	0.051623227	0	0	0	0.008607508	0	0
PHOSPHO1	0.268762681	0.300570467	0.629755029	0.732600733	0.268832074	0	0
SPOP	0	0	0	0	0.012589092	0	0
PDK2	0.001629746	0	0	0.921658986	0.017163569	0	0
PPP1R9B	0	0	0	0	0.010714309	0	0
PCTP	0.001168391	0	0	0	0.033656416	0	0
MSI2	0.00170113	0	0	0	0.006939377	0	0
SUPT4H1	0	0	0	0	0.0316215	0	0
CLTC	0.001422887	0	0	0	0	0	0
USP32	0.00142832	0.031950209	0	0	0.013856557	0	0
MRC2	0.001108959	0.044039477	0	0	0.031300688	0	0
SNHG25	0.868323352	0.103114812	0	0	0.026312211	0.797448	0
AMZ2	0	0	0	0	0.014787193	0	0
WIPI1	0.000944118	0	0	0	0.009776629	0	0
SSTR2	0.016027904	0.113126617	0.732569943	0.732600733	0.058018102	0	0
FAM104A	0.067977485	0.319169775	0.197659709	0	0.32925724	2.367424	0
RPL38	7.22651072	1.620524003	1.381219269	1.504447409	0.357717731	0.797448	0
JPT1	0	0	0	0	0.011939062	0	0
MRPS7	0	0	0	0	0.003821593	0	0
SMIM5	0.00414421	0.111293227	0.154270992	0	0.076550062	0	0
SAP30BP	0.003100925	0	0	0	0	0	0
H3F3B	0	0	0	0	0.004658204	0	0
WBP2	0.031823688	0.26391461	0.569372709	0	0.08160296	0	0
UBALD2	0.01275176	0	0	0	0.141184955	0	0
UBE2O	0	0	0	0	0.003279258	0	0
SEC14L1	0.632652307	0.205906445	0.072702422	0.816326531	0.173470521	0	0
GPS1	0	0.046556019	0	0.004175278	0.008083316	0	0
DUS1L	0.007577101	0	0	0	0	0	0
HEXDC	0	0.023615372	0	0	0	0	0
NARF	0.002318108	0	0	0	0.022655552	0	0
USP14	0.008391169	0	0	0	0.004861803	0	0
LPIN2	0.065454639	0.049377595	0.266437309	0	0.026711187	0	0
MYL12A	0	0	0	0	0.006173783	0	0
MYL12B	0.009850811	0	0	0	0	0	0
DLGAP1-AS	0.00364478	0	0	0	0.00630265	0	0

NDUFV2	0	0	0	0	0.007642272	0	0
ANKRD12	0	0	0.039531942	0	0	0	0
RALBP1	0.569734476	0.112513628	0.287653613	0	0.217534912	0	0
RIOK3	1.214823609	0.400828649	0.451800395	0	0.425698214	0	0
RMC1	0.001661365	0	0	0	0.011614509	0	0
TPGS2	0.086828028	0.172969865	0.351131426	0.501068224	0.171244602	0	0
SLC14A1	0.01321607	0.02671247	0	0	0.08288775	0	0
CTIF	0	0	0	0	0.005652368	0	0
RPL17	0.002272689	0	0	0	0.031471824	0	0
ME2	0	0	0.337879845	0	0	0	0
TXNL1	0.021293162	0	0	0	0.016810808	0	0
FECH	0.32029227	0.587371634	0.323093024	0.079365079	0.171607378	0	0
NARS	0.001330959	0	0	0	0	0	0
AC027097.	0.108679179	0	0	0	0.012199289	0	0
NEDD4L	0.000853835	0.177359821	0.191670021	0	0.011953941	0.548968	0
PIGN	0.86999911	0	47.84898591	4.386229577	0.027051587	5.06047	3.012048
CYB5A	0	0	0	0	0.020521635	0	0
MBP	0.0073744	0	0	0	0.013083279	0	0
CTDP1	0	0	0	0	0.003892611	0	0
PQLC1	0	0.000764287	0	0	0	0	0
CSNK2A1	0	0.034669376	0	0	0.073120731	0	0
PSMF1	0.30366396	1.546934899	2.568467993	1.541173229	0.603830629	0.41855	0
FKBP1A	0	0	0	0	0.010027592	0	0
NSFL1C	0	0	0	0	0.004217843	0	0
MAVS	0	0.050920645	0	0	0	0	0
SMOX	0.327205484	0.149857585	0.292384995	0	0.051209872	1.683502	0
TMEM230	0	0	0	0	0.003071027	0	0
SHLD1	0.002434008	0	0	0	0.01857358	0	0
SNX5	0	0	0	0	0.00426694	0	0
BCL2L1	0.129947321	0.741644287	0.540171036	0.524753947	0.702715875	0	0
ABALON	0.001755761	0.008807895	0	0	0.00458081	0	0
CHMP4B	0.197413954	0.165723555	0	0	0.046866328	0	0
RALY	0	0	0.129083892	0	0.007040876	0	0
EIF2S2	0.045269558	0.040736516	0	0	0.00589727	0	0
EDEM2	0	0	0	0	0.006964176	0	0

MMP24OS	0.240119495	0.22920947	0	0.016806723	0.066816792	0	0
ERGIC3	0.015206316	0	0	0	0.007043492	0	0
CPNE1	0.023850069	0.045262796	0	0	0.01417878	0	0
RBM39	0	0.049377595	0	0	0.007749878	0	0
SCAND1	0.003922626	0	0	0	0.009471843	0	0
RAB5IF	0.003418625	0	0	0	0	0	0
BLCAP	0.001063493	0	0	0	0.005909388	0	0
TGM2	0	0	0	0	0.007129129	0	0
FAM83D	0	0	0	0	0.003868337	0	0
TOP1	0.329066919	0.246495655	0.154948194	1.369047619	0.05406934	0	2.738226
IFT52	0.001290129	0	0	0	0.002405702	0	0
OSER1	0.007051997	0.091359929	0	1.182503752	0.01042718	0	0
SERINC3	0.122445977	0.029626557	0	0	0.011540312	0	0
PKIG	0.002872805	0	0	0	0	0	0
SLC35C2	0	0	0	0	0.004834842	0	0
AL031666.	0.004678947	0	0	0	0.003343498	0	0
STAU1	0.015756078	0.065509152	0	0	0.045449153	0	0
ZFAS1	0.00160183	0	0	0	0.005632629	0	0
UBE2V1	0.207843373	0	0	0	0.037089002	0	0
FAM210B	0.760913321	0.344733195	0.55693883	0.027353577	1.681304487	0	0
AURKA	0	0.007578887	0	0	0	0	0
RTF2	0.003742665	0	0	0	0.011135604	0	0
RBM38	0.094105441	0.517921217	0.640347927	0	0.216016683	0	0
STX16	0	0	0	0	0.004202458	0	0
GNAS	1.231876998	0.418976399	0.334493532	0.279998702	0.419734473	0	0
ATP5F1E	3.715227068	1.764447413	1.320854223	1.365055862	1.835486572	1.975645	0.700476
PRELID3B	0	0.013692947	0	0	0.010310854	0	0
FAM217B	0	0.096224289	0	0	0.002505092	0	0
PSMA7	0.002350024	0.047925313	0	0.274725275	0.011423702	0	0
OSBPL2	0.004183238	0	0	0	0.007123443	0	0
ADRM1	0.205759488	0.069897934	0	0.010546854	0.006039705	0	0
RPS21	0.166011299	0	0	0	0.015217218	0	0
GID8	0.034682	0	0	0	0.05916282	0	0
PPDPF	1.84524517	0.732790112	0.366750116	0.071968334	0.566820496	0.211613	0
PCMTD2	0.017167441	0	0	0	0.009313648	0	0

CDC34	0.06058195	0.155745166	0.158127767	0	0.161437588	0	0
BSG	0.18604229	1.108196933	1.226454064	0.224971879	1.000474517	0	3.543586
RNF126	0.010285558	0	0	0	0	0	0
R3HDM4	1.148816892	1.226062537	0.895814557	1.778635459	0.793997562	0	0
ARID3A	0.044144848	0.032589213	0	0	0	0	0
POLR2E	0	0.036210237	0	0	0.015257276	0	0
GPX4	0.110658655	0.177047137	0.175697519	0	0.144109101	0	0
STK11	0	0	0	0	0.006483407	0	0
ATP5F1D	0.003245352	0	0.069506711	0	0.041883406	0	0
CIRBP	0.001974256	0	0	0	0.003032058	0	0
C19orf24	0	0	0	0	0.013934152	0	0
NDUFS7	0.169107113	0	0	0	0.006592975	0	0
RPS15	4.800913422	0.385379245	6.132903318	1.089892767	0.840785014	2.367424	0
UQCR11	0.324495828	0.34446799	0.447067411	0.840336134	0.484561486	0	0
TCF3	0.072523679	0	0.082144295	0	0.007829668	0	0
ABHD17A	0.034501556	0	0	0	0.004589935	0	0
SF3A2	0.007550902	0	0	0	0	0	0
JSRP1	0	0.040736516	0	0	0.004789616	0	0
OAZ1	3.592592891	11.80476409	12.59772993	13.2148868	6.001755723	12.11492	2.801905
TMPRSS9	0.00457652	0.080713377	0	0	0.038438799	0	0
AES	0.012352235	0	0	0	0.014275582	0	0
SMIM24	0.165250443	0	0	0	0.053745877	0	0
DOHH	0	0	0	0	0.005458921	0	0
FZR1	0	0	0	0	0.007165853	0	0
MFSD12	0	0	0	0	0.004181756	0	0
EEF2	0.499958955	0	0.162182325	2.617086809	0.280424029	0	3.543586
MAP2K2	0.001191903	0.022019738	0.119341711	0	0.019076162	0	0
YJU2	0.001026624	0	0	0	0.00426074	0	0
SH3GL1	0	0	0	0	0.020232479	0	0
UBXN6	0.955123337	1.232977579	0.806741684	0.548817904	0.831037551	3.218681	0
DPP9	0	0	0	0	0.003837505	0	0
FEM1A	0.126916438	0.128404772	0	0	0.009658978	0	0
KDM4B	0.001019117	0	0	0	0	0	0
RPL36	1.012914871	0.194994875	0	0.130463144	0.172498307	1.052189	0
NDUFA11	0.001986395	0.038796682	0.154270992	0	0.015507691	0	0

ALKBH7	0.001273257	0	0	0	0	0	0
GTF2F1	0	0	0	0	0.008595274	0	0
TEX45	0.118455342	0	0	0	0	0	0
MCOLN1	0.012815217	0.120163146	0.074413067	0	0.077820629	0.923873	0
PNPLA6	0	0	0	0	0.003479459	0	0
PET100	0.090442531	0.041781042	0	0	0	0	0
AC008763.	0.018508647	0	0	0	0	0	0
STXBP2	0	0.046556019	0	1.189474949	0.043260664	0	0
TRAPPC5	0.024540452	0.040736516	0	0.003170728	0.017511826	0	0
MAP2K7	0	0	0	0	0.02034212	0	0
SNAPC2	0	0	0	0	0.003343498	0	0
NDUFA7	0.001982516	0	0	0	0.005349596	0	0
RPS28	0.671379205	0.202024212	0.420476108	0.004175278	0.105076087	0	0
RAB11B	0.071453353	0.007578887	0	0	0.157770461	0	0
2-Mar	0.186595237	0.042386282	0.180717448	0	0.070098809	0.327955	0
UBL5	0.183877895	0.071481057	0	0	0.191574375	0	0
PIN1	0	0	0.097309395	0	0.002971998	0	0
CDC37	0	0.007274378	0	0	0.006483871	0	0
CDKN2D	0	0.017334688	0	0	0.039836525	0	0
SLC44A2	0	0	0	0	0.017318142	0	0
TMED1	0	0	0	0	0.003587418	0	0
CARM1	0	0	0	0	0.016299712	0	0
YIPF2	0.003100925	0	0.154270992	0	0.026788469	0	0
KANK2	0	0	0	0	0.023834673	0	0
AC011472.	0.002442378	0	0	0	0	0	0
ZNF653	0.012570166	0	0	0	0.003532421	0	0
ECSIT	0.031492058	0.159446477	0.170948938	0	0.151367376	1.942502	0
ELOF1	0.105918037	0.184015477	0	0	0.159867044	0	0
ACP5	0	0.006291354	0	0	0.022814363	0	0
WDR83OS	0.104861983	0.004893275	0	0.732600733	0.04384484	0	0
DHPS	0.00764879	0	0	0	0.105611864	0	0
TRIR	0	0	0	0	0.020156588	0	0
ASNA1	0.023001037	0.169330377	0	0	0.032438411	0	0
PRDX2	0.212275452	0.390414382	0.224147758	0	0.287054034	0.923873	0
DNASE2	0.001122445	0	0	0	0.017183139	0	0

KLF1	0.162525616	0.125919226	0.186032667	0.004175278	0.048127633	0	3.886514
CALR	0	0.050920645	0	0	0.030816933	0	0
RAD23A	0.06738789	0.451486984	0.124021778	0.875989205	0.162416962	0	0
NFIX	0.372508612	0.607301559	0.463653528	0.003170728	0.392557009	0.533504	3.170577
LYL1	0.185554625	0.133581786	0	0	0.112629839	0	0
CCDC130	0.010124388	0	0	0	0	0	0
C19orf53	0.020139004	0	0	0.064495324	0.01860063	0	0
TECR	0	0.035423058	0	0	0.002942449	0	0
BRD4	0.001026624	0.02295015	0	0	0.023588278	0	0
BABAM1	0.026787141	0.303263473	0.215176722	0	0.137762675	0	0
ANKLE1	0.001545742	0	0	0	0	0	0
DDA1	0	0	0	0	0.010497525	0	0
PLVAP	0	0.041781042	0	0	0.010857632	0	0
RPL18A	2.530181234	1.069184172	0.614526983	1.686507937	0.304344911	3.333188	0
CCDC124	0.055683093	0.047925313	0	0	0.042377191	0	0
JUND	0.001735186	0.050920645	0	0	0.013704678	0	0
FKBP8	8.253981422	14.06160949	14.62602242	18.48493074	6.11318592	10.99195	20.0574
KXD1	0.001020287	0.076206398	0	0	0.007779787	0	0
UBA52	64.96715823	12.87628637	14.7441289	17.35682388	6.227754701	26.59048	11.08411
COPE	0.000946129	0.119627885	0.105418511	0	0.022417931	0	0
DDX49	0.020382469	0.056427475	0	0	0.006545873	0	0
RFXANK	0	0	0	0	0.029478056	0	0
NDUFA13	0	0	0	0	0.003543285	0	0
AC123912.	0	0.123248391	0.175697519	0	0.005639522	0	0
AC123912.	0.00306218	0	0	0	0	0	0
UQCRFS1	0	0	0	0	0.002281173	0	0
GPATCH1	0	0.050920645	0	0	0	0	1.798238
LSM14A	0.074992425	0.045262796	0.176157189	1.423426513	0.047595663	0	0
ZNF302	0	0.273660349	0.474992266	0	0	0.327955	0
TMEM147	0.00229936	0	0	0	0	0	0
COX6B1	0.635601455	0.510611196	0.373357228	0.019175455	0.119780589	0	2.563445
POLR2I	0.010985039	0	0	0	0.012361706	0	0
CAPNS1	0.00236674	0	0	0	0	0	0
EIF3K	0.118246645	0.245500803	0.162182325	0	0.082723703	0	0
ECH1	0	0.132648084	0	0	0.007353793	0	0

RINL	0.007157422	0	0	0	0	0	0
RPS16	0.038749189	0	0	0	0.038480824	0	0
SUPT5H	0	0	0	0	0.011933526	0	0
DYRK1B	0.021723317	0	0	0	0	0	0
AKT2	0	0	0	0	0.00915202	0	0
BLVRB	2.140328401	3.999801447	4.247647566	4.069401297	2.056587997	9.03086	0
B3GNT8	0	0	0	0	0.004214537	0	0
RPS19	0.644687477	0.070911713	0.322793117	0	0.047348101	0	0
DEDD2	0	0	0	0	0.006974833	0	0
LIPE-AS1	0.002644092	0	0	0	0	0	0
PSG2	0	0.1207116	0.158127767	0	0	0	0
ZNF428	0.012804893	0	0	0	0.041363519	0	0
ZNF283	0	0	0	0	0.00458081	0	0
BCAM	0.003436809	0	0	0	0.03509897	0	0
CLPTM1	0	0.041781042	0	0	0.008822162	0	0
RELB	0.069471768	0	0	0	0	0	0
PPM1N	0	0	0.086645352	0	0	0	0
SNRPD2	0.001110519	0	0	0	0.008595274	0	0
CALM3	0.014214163	0.262754316	0	0.004175278	0.084402798	0	0
DACT3	0	0.201010014	0.100015813	0	0	0	0
SLC1A5	0	0.017711529	0	0	0.064781802	0	0
AP2S1	0.080371023	0.235832592	0.352482949	0	0.143242483	0	0
NAPA	0.139310467	0.209784833	0.175697519	0.004175278	0.186067055	0	0
KDELR1	0.006232849	0	0	0	0.002405702	0	0
RPL18	0.221917881	0.228602845	0	0	0.042662068	0	0
NUCB1	0.063201544	0.032589213	0	0	0.085148451	0	0
FTL	37.89481051	32.08602977	39.23465271	28.03426729	8.617400139	29.52205	63.92363
RPL13A	0.608937427	0.290452266	0.444151219	0.896027871	0.241157578	0	1.54464
RPS11	5.868315933	1.399631899	1.007909616	1.015153958	0.644521336	6.806449	4.916692
AP2A1	0.004183238	0.053492395	0	0	0.125930822	0	0
MED25	0.013530698	0.281834382	0.191670021	0.56022409	0.089404181	0	0
AKT1S1	0.060195802	0.010935978	0.186032667	0	0.025786588	0	0
TBC1D17	0.006129854	0	0	0	0.00475432	0	0
VRK3	0	0	0.137502406	0	0	0	0
ETFB	0	0.035423058	0	0.003170728	0.009139145	0	0

NDUFA3	0.030270613	0.091779023	0.351395038	0	0.087290004	0.642013	0
RPS9	0.221993351	0.212301487	0	1.343101343	0.098332461	0	0
TMEM86B	0.0421006	0	0.137502406	0.892857143	0.028462883	0	0
PPP6R1	0	0	0	0	0.013006185	0	0
RPL28	1.431386085	0.535390904	0.601164298	0.921658986	0.363040687	0	0
UBE2S	0	0	0	0.426439232	0.008304063	0	0
ZNF580	0.002359668	0	0	0	0.005638536	0	0
EPN1	0.002555442	0	0.197659709	0	0.017354663	0	0
ZSCAN5A	0	0.02671247	0	0	0.004897975	0	0
RPS5	0.412462664	0.1070566	0	0	0	0	0
CHMP2A	0.007577101	0.032589213	0	0	0.037377242	0	0
ZFY	0	0.044039477	0	0	0	0	0
AC010722.	0	0.084775993	0	0	0	0	0
EIF1AY	0.035838229	0.294947365	0.671932983	0	0.623623822	0	0
BCL2L13	0.028316018	0.041781042	0	0	0.030382486	0	0
MICAL3	0.00102293	0	0	0	0	0	0
C22orf39	0.001330959	0	0	0	0.010092903	0	0
UFD1	0.001063493	0.053321781	0.150597874	0	0.035124261	1.578283	0
TXNRD2	0	0.088815079	0	0	0.012380513	0	0
COMT	0.021865304	0	0.471673646	0	0.027547455	0	0
TANGO2	0.091256981	0.238700525	0	0.569958435	0.239439656	1.578283	0
MED15	0.006265336	0	0	0	0.004202458	0	0
GUCD1	0.037822865	0.015518673	0	0.865800866	0.435616899	0	0
SNRPD3	0.001982516	0	0	0	0	0	0
SRRD	0.012881193	0.009364716	0	0	0.148727926	0	0
TPST2	0.003099573	0	0	0	0.008305372	0	0
TTC28	0	0.154463247	0.771101597	0.664451827	0	0	0
CCDC117	0.003058227	0	0	0	0	0	0
EMID1	0.008178735	0	0	0	0.007511745	0	0
ASCC2	0.215275755	0.774763346	1.206159889	0.848686691	0.536279023	0	2.362391
MTMR3	0.001125645	0	0	0	0.012211143	0	0
CASTOR1	0	0	0	0	0.003837505	0	0
SEC14L3	0.011408219	0	0	0	0.01129736	0	0
AC004832.	0	0	0	0	0.005499159	0	0
OSBP2	0.188688228	0.149792017	0	0	0.077155372	0.701459	0

FBXO7	1.781662404	4.946979532	3.882032988	3.008466739	2.261111524	3.989968	7.53104
Z95114.4	0	0.091158638	0	0	0	0	0
TXN2	0.005846986	0.041781042	0	0.064495324	0	0	0
EIF3D	0	0	0	0	0.003304114	0	0
MPST	0.002218265	0	0	0	0.016606333	0	0
H1F0	0	0	0	0	0.023783787	0	0
GCAT	0	0	0	0	0.005259753	0	0
POLR2F	0	0.042880543	0	0	0.007000577	0	0
DDX17	0	0.013247648	0	0	0.002405702	0	0
GTPBP1	0	0	0	0	0.003587418	0	0
APOBEC3C	0.09136036	0	0	0	0.002283757	0	0
RPL3	0.305987429	0.0653527	0.166450281	0.663059934	0.050028982	0	0
ATF4	0.008667464	0.035423058	0	0	0.0172177	0	0
RPS19BP1	0.003245352	0	0	0	0.007110423	0	0
GRAP2	0.003436809	0	0	0	0.00458081	0	0
TNRC6B	0.010333167	0	0	0	0	0	0
ST13	1.391781341	1.11460075	1.189518674	0.696864111	0.509110426	2.047502	2.190581
RBX1	0.152550284	0.050920645	0	0	0.066479621	0	0
PHF5A	0	0	0	0	0.004569131	0	0
ACO2	0	0.050920645	0	0	0.010340707	0	0
DESI1	0.005653498	0	0	0.680272109	0.02423919	0	0
SMDT1	0.072094031	0.070751947	0.221451244	0	0.028599375	0	0
NDUFA6	0	0	0	0	0.003279258	0	0
CYB5R3	0.021726574	0	0	0	0.042468668	0	0
TSPO	0.088148466	0.116800388	0.112948405	0.7753715	0.017190269	0	0
PRR5	0.011853529	0.119946409	0.137502406	0	0.062106577	0	0
ATXN10	0	0	0	0	0.003587418	0	0
PRR34-AS1	0.011786695	0	0	0	0.010363683	0	0
PPARA	0	0.037894434	0	0	0	0	0
TBC1D22A	0	0	0	0	0.014133282	0	0
FP236383.	0	0.050920645	0	0	0	0	0
CR381653.	0.001970971	0	0	0	0	0	0
LINC02573	0.028052831	0	0	0	0.008115236	0	0
ATP5PF	0.001545742	0.040736516	0	0	0.003279258	0	0
CCT8	0	0	0	0	0.004181756	0	0

SOD1	0.003817036	0	0	0	0.025766856	0	0
DONSON	3.745336218	42.82070459	47.47420067	41.78646637	0.335486015	33.07609	57.11018
ITSN1	0.268263272	0	0	0	0.002405702	0	0
ATP5PO	0	0	0	0	0.015393602	0	0
NDUFV3	0.02817369	0	0	0.0999001	0.023402859	0	0
CSTB	0	0.000853121	0	0	0.016838311	0	0
SUMO3	0.003100925	0	0	0	0	0	0
YBEY	0.00186446	0.06083804	0	0	0.024240127	0	0
C21orf58	0	0	0.134576823	0	0.010507461	0.99681	0
MT-ND2	0.001026328	0	0.204035829	0	0.002387894	0	2.077275
MT-CO1	0.00422969	0.282949295	0.83205231	0.418253953	0.171177444	1.721763	0
MT-CO2	0.000853835	0.037033197	0.079063884	0	0.018039009	0	0
MT-CO3	0.22176588	0.063800781	0.097309395	0	0.023298886	0	0
MT-ND3	0.001026328	0	0	0	0.008537834	0	0
MT-ND4L	0.000853835	0	0	0	0.00384987	0	0
MT-ND4	0.044144848	0.210718342	0	0	0.035353252	0	0
MT-ND5	0	0.198236528	0.150597874	0	0.017564105	0	0
MT-CYB	0.030503522	0	0	0	0.010951056	0	0
AURKAIP1	0.009182585	0	0	0	0.005591622	0	0
GNB1	0	0	0	0	0.008457804	0	0
PARK7	0	0	0	0	0.008451157	0	0
USP48	0.019964384	0	0	0	0	0	0
FUCA1	0	0	0	0	0.002636755	0	0
SYF2	0	0	0	0	0.115461391	0	0
TMEM50A	0	0	0	0	0.008123411	0	0
AL020996.	0	0	0.191670021	0	0	0	0
UBXN11	0	0.038796682	0	0	0	0	0
THEMIS2	0	0	0	0	0.004499033	0	0
LAPTM5	0.002096555	0	0	0	0	0	0
BSDC1	0	0	0	0	0.008457804	0	0
HYI	0	0	0	0	0.002636755	0	0
KDM4A	0	0	0	0	0.011286936	0	0
HECTD3	0	0	0	0	0.008226999	0	0
GPBP1L1	0	0	0	0	0.004270894	0	0
MKNK1	0	0	0	0	0.009882682	0	0

MOB3C	0	0	0	0	0.002283757	1.99362	0
EFCAB14	0	0	0	0	0.004467876	0	0
ECHDC2	0	0	0.180717448	0	0	0	0
MRPL37	0	0	0	0	0.009700215	0	0
DNAJC6	0	0	0	0	0.00372409	0	0
LEPROT	0.173349281	0	0	0	0	0	0
WDR78	0.005253009	0	0	0	0	0	0
DNAJB4	0	0	0	0	0.00403781	0	0
C1orf52	0	0	0	0	0.055617353	0	0
PKN2	0.005883543	0	0	0	0	0	0
ZNF326	0.002227302	0	0	0	0	0	0
FAM102B	0	0.021440272	0.332228036	0	0	0	0
DRAM2	0	0	0	0	0.055617353	0	0
HIPK1	0.048680278	0	0	0	0.005286474	0	0
NRAS	0	0	0.060239149	0	0	0	0
RNF115	0	0	0	0.003170728	0.007709862	0	0
HIST2H2A/	0	0	0	0.003170728	0	0	0
HIST2H2A(	0	0	0	0	0.003010271	0	0
ANP32E	0.007621922	0.049377595	0	0	0	0	0
CRTC2	0	0	0	0	0.004897975	0	0
TPM3	0	0.083899028	0	0.739918609	0.010511698	0	0
ZBTB7B	0.001212853	0	0	0	0	0	0
ASH1L-AS1	0	0	0	0	0.003508981	0	0
COPA	0	0	0	0.005273427	0	0.94697	0
PPOX	0.006127	0	0	0	0	0	0
MGST3	0.002312828	0.074214674	0	0	0.003238761	0	0
Z97198.1	0	0	0	0	0.003337271	0	0
STX6	0	0	0	0	0.006545873	0	0
KIF14	0	0	0	0	0.00566229	0	0
TMEM9	0	0	0	0	0.008457804	0	0
NAV1	0	0	0.170948938	0	0	0	0
DSTYK	0.001212853	0	0	0	0	0	0
SRGAP2	0	0	0	0	0.003654891	0	0
MAPKAPK2	0	0	0	0	0.003136114	0	0
CD55	0	0	0	0	0.006856124	0	0

CR1	0	0.052563247	0	0	0.055617353	0	0
NENF	0.005792437	0	0	0	0	0	0
NSL1	0	0	0.020602966	0	0.005286474	0	0
CENPF	0.034839807	0	0	0	0	0	0
EPRS	0.187034751	0	0	0	0	0	0
MRPL55	0.001567907	0	0	0	0	0	0
IBA57	0	0	0	0	0.003786602	0	0
RAB4A	0	0.047925313	0	0	0	0	0
EFCAB2	0	0.050920645	0	0	0.004897975	0	0
ADI1	0.001237993	0.130713563	0	0	0	0	0
RSAD2	0	0	0	0	0.012810987	0	0
IAH1	0	0	0	0	0.003226129	0	0
ROCK2	0	0	0	0	0	0.642013	0
GREB1	0	0	0.147095597	0	0	0	0
SMC6	0.00166292	0.17417472	0	0.064495324	0.005881142	0	0
SF3B6	0	0	0	0	0.137854977	0	0
PTRHD1	0	0.003754518	0	0	0.005149755	0	0
HADHB	0.003758498	0	0	0	0	0	0
PSME4	0.100103106	0	0	0	0	0	0
SNRNP27	0	0	0	0	0.004362145	0	0
FAM136A	0	0	0	0	0.003298033	0	0
EXOC6B	0.001485333	0	0	0	0	0	0
BOLA3	0	0	0.027620571	0	0.003790607	0	0
RNF181	0.034006318	0	0	0	0	0	0
MAL	0	0	0.112948405	0	0.009971817	2.047502	0
ITPRIPL1	0	0.090663588	0	0	0	0	0
EIF5B	0	0	0	0	0.006062406	0	0
ZC3H6	0.00764228	0	0	0	0.005286474	0	0
EPC2	0	0	0	0	0.005256325	0	0
BAZ2B	0.004042844	0.106128376	0	0	0.004567514	0	0
DYNC1I2	0	0	0	0	0.137854977	0	0
NFE2L2	0	0.074446221	0	0	0.003136114	0	0
PDE11A	0	0	0.33311972	0	0	0	0
ITGA4	0.010167841	0	0	0	0	0	0
SLC40A1	0	0	0	0	0.063178594	0	0

FZD5	0	0	0	0	0.004464784	0	0
ZFAND2B	0	0	0	0	0.008370022	0	0
RHBDD1	0	0	0	0	0.003607074	0	0
RNPEPL1	0	0	0	0	0.019235578	0	0
PPP1R7	0	0	0	0	0.003774639	0	0
FGD5-AS1	0.02292684	0	0	0	0	0	0
RBSN	0	0	0	0	0.005230755	0	0
CAPN7	0.009982192	0	0	0	0	0	0
MOBP	0	0.045262796	0	0	0	0	0
SS18L2	0.00306218	0	0	0	0	0	0
ABHD5	0	0	0	0	0.137854977	0	0
TMEM42	0.002961384	0	0	0	0	0	0
LZTFL1	0.002684033	0	0	0	0	0	0
RHOA	0	0	0	0	0.01728728	0	0
ABHD14B	0	0	0	0	0.005472106	0	0
DNAH1	0	0.044039477	0	0	0	0	0
THOC7	0.019365996	0	0	0	0.01377979	0	0
CADM2	0	0	0	0	0.004347448	0	0
CHMP2B	0	0	0	0	0.004418913	0	0
DZIP3	0.001665648	0.044039477	0.03880436	0	0.009369174	0	0
NAA50	0.008963342	0	0	0	0	0	0
GSK3B	0	0	0	0.840336134	0	0	0
KPNA1	0	0	0	0	0.003505168	0	0
CDV3	0.002693187	0.004750614	0	0	0.017724211	0	0
PCCB	0	0	0	0	0.004362145	0	0
RNF7	0	0	0	0	0.00426074	0	0
XRN1	0	0	0	0	0.005018367	0	0
MFSD1	0	0.046556019	0.110966854	0	0.008123411	0	0
TBL1XR1	0.006362978	0	0	0	0.005256325	0	0
FXR1	0	0	0	0	0.023269652	0	0
FAM131A	0	0	0	0	0.003226129	0	0
TFRC	0	0.038796682	0	0	0	0	0
LINC00885	0.002378621	0.032589213	0	0.714285714	0.005337205	0	0
NELFA	0	0	0	0	0.008006469	0	0
POLN	0.187034751	0	0	0	0	0	0

HTT	0	0.032589213	0	0	0.006489251	0	0
MED28	0	0	0	0	0.007542218	0	0
UBE2K	0	0	0	0	0.003298033	0	0
TMEM165	0	0	0	0	0.005412881	0	0
PAICS	0.033057305	0	0	0	0.002901949	0	0
MOB1B	0.031036334	0.013247648	0	0.030362836	0	0	0
HNRNPDL	0	0	0.121636744	0	0.002901949	0	0
AFF1	0.005888418	0	0	0	0	0	0
AC097478.	0	0.032589213	0	0	0.00384987	0	0
AP001816.	0	0	0	0	0.002283757	0	0
MANBA	0	0	0.175697519	0	0	0	0
ELF2	0.071073205	0.089621025	0	0.352733686	0	0	0
GAB1	0.00459426	0.036210237	0	0	0	0	0
ETFDH	0	0	0	0	0.00458016	0	0
NEK1	0	0	0	0	0.011907739	0	0
AC097534.	0	0	0	0	0.003238761	0	0
SPATA4	0.088841507	0	0	0	0	0	0
ING2	0.003489112	0	0	0	0	0	0
FRG1	0	0	0	0	0.00793583	0	0
SLC6A19	0	0	0	0	0.005018367	0	0
SREK1IP1	0.173349281	0	0	0	0	0	0
CENPK	0.036261839	0.052563247	0	0	0	0	0
MAST4	0	0	0.097309395	0	0	0	0
CENPH	0	0.045262796	0	0	0	0	0
MRPS36	0	0	0	0	0.055617353	0	0
BDP1	0	0.027617977	0	0	0.013744382	0	0
TNPO1	0	0	0	0	0.004226686	0	0
LYSMD3	0	0	0	0	0.004270894	0	0
C5orf63	0.002157002	0	0	0	0	0	0
SLC22A4	0	0	0	0	0.002907966	0	0
UQCRQ	0.001735186	0	0	0	0	0	0
CTNNA1	0	0	0	0	0.002802369	0	0
SRA1	0	0	0	0	0.007542218	0	0
RNF145	0	0	0	0	0.003774639	0	0
TTC1	0	0	0	0	0.006099086	0	0

STK10	0	0	0	0	0.006831441	0	0
AC106795.	0	0	0.158127767	0	0	0.102792	0
FAM153C	0	0	0	0	0.005286474	0	0
TMEM14C	0	0	0	0	0.009999862	0	0
NEDD9	0	0.030744541	0	0	0	0	0
NOL7	0	0	0	0	0.004270894	0	0
RANBP9	0	0.049377595	0	0	0	0	0
MCUR1	0.00276227	0	0	0	0	0	0
DEK	0	0.046556019	0	0	0	0	0
TRIM15	0	0	0	0	0.002907966	0	0
FLOT1	0.01536371	0	0	0	0	0	0
AGPAT1	0	0	0	0	0.004128768	0	0
RNF5	0	0	0	0.892857143	0.003136114	0	0
AGER	0.029009472	0	0	0	0	0	0
HMGA1	0	0	0	0	0.003607074	0	0
AL451165.	0	0	0	0	0.004058129	0	0
TBC1D22B	0	0.016133274	0	0	0.013673031	0	0
BTBD9	0	0	0.372065335	0.816326531	0	0	0
TAF8	0	0	0	0	0.003607074	0	0
RHAG	0	0	0	0	0.005286474	0	0
PHIP	0	0	0	0	0.004716091	0	0
LYRM2	0.01333456	0	0	0	0	0	0
FBXL4	0.031036334	0	0	0	0	0	0
AK9	0.022280002	0	0	0	0	0	0
HDAC2	0	0	0	0	0.003505168	0	0
STX7	0	0	0	0	0.003032058	0	0
SLC18B1	0	0.045262796	0	0	0.006128078	0	0
HECA	0.020600929	0	0	0	0	0	0
CITED2	0	0	0	0	0.002597477	0	0
ADAT2	0	0.049377595	0	0	0	0	0
AL023806.	0	0	0	0	0.003308859	0	0
FBXO30	0.003647586	0	0	0	0	0	0
TAB2	0	0	0	0	0.00566229	0	0
PCMT1	0	0	0	0	0.00700653	0	0
ARID1B	0	0	0	0	0.005337205	0	0

C6orf120	0	0	0	0	0.005011351	0	0
MAFK	0	0	0	0	0.013309298	0	0
EIF3B	0	0	0	0	0.003010271	0	0
C7orf26	0	0.052563247	0	0	0	0	0
C1GALT1	0	0.007178241	0	0	0.006751633	0	0
CBX3	0.116513451	0	0	0	0	0	0
SUMF2	0	0	0	0	0.004954395	0	0
ZNF273	0	0	0	0	0.004362145	0	0
AC027644.	0	0	0	0	0.011126067	0	0
TP53TG1	0	0	0	0	0.006489251	0	0
VPS50	0	0	0	0	0.275709953	0	0
COPS6	0	0	0	0	0.002706441	0	0
TRIM24	0	0.049377595	0	0	0	0	0
ZC3HAV1	0	0	0	0	0.009094934	0	0
FMC1	0	0	0	0	0.004897975	0	0
TMUB1	0	0.038796682	0	0	0.006489251	0	0
ABCF2.1	0	0	0	0	0.002907966	0	0
GTPBP6	0	0	0	0	0.005632629	0	0
ASMTL-AS:	0	0	0	0	0.003337271	0	0
AC073529.	0.000946129	0	0	0	0	0	0
EIF1AX	0.187034751	0	0	0	0.008791642	0	0
TSPAN7	0	0	0	0	0.00393535	0	0
ATP6AP2	0	0	0	0	0.005632629	0	0
NDUFB11	0.01083433	0.036210237	0	0	0.004125072	0	0
ARAF	0	0	0	0	0.005842794	0	0
FTSJ1	0.003504596	0	0	0	0.006062406	0	0
HDAC6	0	0	0	0	0.004647466	0	0
PIM2	0	0	0	0	0.003337271	0	0
OTUD5	0	0	0	0	0.005680237	0	0
HSD17B10	0	0	0	0	0.004464784	0	0
ARHGEF9	0	0.027157677	0	0	0	0	0
IGBP1	0	0	0	0	0.004837017	0	0
NDUFA1	0	0	0	0	0.006452258	0	0
MECP2	0	0.011807686	0.175697519	0	0.005286474	0	0
FAM50A	0	0	0	0	0.005592591	0	0

SLC10A3	0	0	0	0	0.003774639	0	0
FAM167A	0.030901394	0	0	0	0	0	0
AC107959.	0.012624015	0.07909078	0	0	0.009813832	2.104377	0
PLPBP	0	0	0	0	0.003010271	0	0
SLC20A2	0	0	0	0	0.004226686	0	0
VCPIP1	0	0.050920645	0.170948938	0	0.008998065	0	0
CA2	0	0	0	0	0.066582815	0	0
LRRC69	0	0.085819747	0.536793426	0.816326531	0	0	0
AZIN1	0	0	0	0	0.003298033	0	0
OXR1	0.006544494	0.055762011	0	0	0	0	0
EBAG9	0	0.107617836	0.140558015	0	0.002283757	0	0
DERL1	0	0.050920645	0	0	0.005149755	0	0
CPSF1	0	0	0	0	0.003786602	0	0
DOCK8	0	0.041781042	0	0	0	0	0
PUM3	0.020364815	0.047925313	0.158127767	0	0	0	0
DCTN3	0	0	0	0	0.002706441	0	0
ZCCHC7	0.003136505	0	0	0	0	0	0
FAM122A	0	0	0	0	0.010477644	0	0
PTAR1	0.187034751	0.042880543	0	0	0	0	0
SMC5	0	0.038796682	0	0	0	0	0
FRMD3	0	0.005882529	0	0	0	0	0
AL354920.	0.008451035	0	0	0	0	0	0
HNRNPK	0.01483783	0.301283926	0.196300486	0	0.015129941	0	0
SEC61B	0	0.030175197	0	0	0.002283757	0	0
PTBP3	0.008264326	0	0	0	0	0	0
ALAD	0	0.014813279	0	0	0.002636755	0	0
POLE3	0.002254861	0	0	0	0	0	0
PDCL	0	0	0	0	0.005632629	0	0
DENND1A	0.002227302	0	0	0	0	0	0
GAPVD1	0	0	0	0	0.055617353	0	0
SURF1	0	0	0	0	0.004179047	0	0
UBAC1	0.004271226	0	0	0	0.003774639	0	0
FBXW5	0	0.050920645	0.121636744	0	0.008970057	0	0
TAF10	0.004238116	0	0	0	0	0	0
SOX6	0	0.050920645	0	0	0	0	0

TSG101	0.004439301	0	0	0	0.003032058	0	0
MTCH2	0	0	0	0	0.003790607	0	0
SDHAF2	0.001963348	0	0	0	0	0	0
FADS3	0	0.046556019	0	0	0	0	0
BEST1	0	0.042880543	0	0	0	0	0
COX8A	0.001212853	0	0	0	0	0	0
FERMT3	0.002332563	0	0	0	0	0	0
EHD1	0	0	0	0	0.017228524	0	0
SNX15	0	0	0	0	0.00458016	0	0
TM7SF2	0	0	0.175697519	0	0.004270894	0	0
NEAT1	0.012624015	0.013035685	0.29047206	0	0.002283757	0	0
FAM89B	0	0	0	0	0.005287341	0	0
RNASEH2C	0	0	0	0	0.011066982	0	0
AP003716.	0	0	0.197659709	0	0	0	0
ANAPC15	0	0	0	0	0.002901949	0	0
PICALM	0	0.031335782	0.175697519	0	0.004464784	0	0
CHORDC1	0	0	0	0	0.005316095	0	0
AP000787.	0.001560676	0	0	0	0.01728728	0	0
PTS	0	0	0	0	0.008451157	0	0
SIDT2	0	0.038796682	0	0	0.004845736	0	0
RNF214	0.04683038	0	0	0	0	0	0
EI24	0	0.050920645	0	0	0	0	0
PRPF18	0	0	0.204035829	0	0.004160184	0	0
HNRNPF	0	0	0	0	0.003774639	0	0
ASAH2B	0.025474267	0	0	0	0	0	0
CCAR1	0	0	0	0	0.007590133	0	0
DNAJB12	0	0.030873416	0	0.178571429	0.013503315	0	0
MICU1	0.00811338	0	0	0	0	0	0
ANXA7	0	0	0	0	0.018818507	0	0
NUTM2E	0	0	0	0	0.006540565	0	0
PTEN	0	0	0	0	0.013261571	0	0
FRA10AC1	0.077253484	0	0	0	0	0	0
RRP12	0	0	0	0	0.005632629	0	0
BLOC1S2	0.007759084	0	0	0	0	0	0
NDUFB8	0	0	0	0	0.004418913	0	0

BORCS7	0	0	0	0	0.011243661	0	0
ATE1	0	0	0	0	0.003790607	0	0
ZRANB1	0.002961384	0.044039477	0	0	0	0	0
TUBGCP2	0	0	0	0	0.005287341	0	0
AC021054.	0.001567215	0	0	0	0.006489251	0	0
SMIM10L1	0	0	0	0	0.003778618	0	0
ART4	0	0	0	0	0.137854977	0	0
ETFBKMT	0	0.032589213	0	0	0	0	0
PRR13	0.020600929	0.114774981	0.197659709	0	0	0	0
MAP3K12	0.072523679	0	0	0	0.009203442	0	0
COPZ1	0.002959881	0.030744541	0	0.005273427	0	0	0
AC034102.	0	0	0	0	0.008006469	0	0
STAT6	0.003347772	0	0	0	0.004362145	0	0
FAM19A2	0.029988694	0	0	0	0	0	0
RAB3IP	0	0	0	0	0.003337271	0	0
C12orf29	0	0	0	0	0.005621831	0	0
SCYL2	0.000946129	0.008531208	0	0	0	0	0
TDG	0.002405999	0	0	0	0	0	0
PPP1CC	0	0	0	0	0.005842794	0	0
SDSL	0	0	0	0	0.005842794	0	0
ACADS	0.001659813	0	0	0	0.005286474	0	0
WDR66	0	0.030744541	0.166450281	0	0	0	0
MTIF3	0	0	0	0	0.020665	0	0
HMGB1	0	0	0	0	0.006062406	0	0
SETDB2	0	0	0	0	0.005802854	0	0
SPRYD7	0	0	0	0	0.003298033	0	0
RBM26	0	0	0	0	0.004954395	0	0
ABCC4	0.016567181	0	0	0	0	0	0
UBAC2	0	0	0.052273642	0	0.005394325	2.295684	0
TPP2	0	0.049377595	0	0	0	0	0
DAD1	0	0	0	0	0.005018367	0	0
OXA1L	0	0	0.170948938	0	0	0	0
RBM23	0.21537335	0	0	0	0.017131208	0	0
THTPA	0	0	0	0	0.003298033	0	0
PSME2	0	0	0	0	0.009784066	0	0

NOVA1	0	0	0.197659709	0	0	0	0
ARHGAP5	0	0	0	0	0.005192027	0	0
PPP2R3C	0.006820845	0	0	0	0	0	0
PSMA6	0	0	0	0	0.002706441	0	0
TRAPPC6B	0.173349281	0	0	0	0	0	0
MIS18BP1	0	0	0	0	0.00458016	0	0
POLE2	0.004286683	0	0	0	0	0	0
ARF6	0	0	0	0	0.004125072	0	0
GMFB	0	0	0.121636744	0	0	0	0
FBXO34	0	0	0	0	0.00458016	0	0
ARG2	0	0.046556019	0	0	0.004125072	0	0
DCAF5	0	0.258332236	0.124021778	0	0.007010337	0	0
RGS6	0.003468678	0	0	0	0.00609678	0	0
FCF1	0.001665648	0	0	0	0.009369174	0	0
TMED10	0.009816741	0	0	0	0	0	0
PSMC1	0	0.047925313	0	0	0	0	0
ATXN3	0.173349281	0	0	0	0	0	0
PAPOLA	0	0	0	0	0.005557902	0	0
MEG3	0	0	0.186032667	0	0	0	0
HSP90AA1	0	0	0	0.003170728	0	0	0
ZNF770	0.029613836	0	0	0	0	0	0
EIF2AK4	0.001646738	0	0	0	0	0	0
RMDN3	0	0	0	0	0.008298062	0	0
B2M	0	0	0	0	0.00384987	0	0
SNX22	0	0	0	0	0.011183243	0	0
CLPX	0.002332563	0	0	0	0.005591622	0	0
DENND4A	0.001360253	0	0	0	0	0	0
C15orf61	0	0	0	0	0.013818621	0	0
FEM1B	0	0	0	0	0.005604737	0	0
AC010931.	0	0.050920645	0	0	0	0	0
ARID3B	0	0	0	0	0.004897975	0	0
C15orf40	0	0	0	0	0.005011351	0	0
AEN	0	0	0	0	0.002283757	0	0
IDH2	0	0.011160689	0	0	0.006074364	0	0
HDDC3	0	0.037894434	0	0	0.003238761	0	0

LRRC28	0.015026048	0	0	0	0	0	0
AC140725.	0	0	0	0	0.007846768	0	0
SNRNP25	0	0	0	0	0.004125072	0	0
RHOT2	0	0	0	0	0.005842794	0	0
FAHD1	0	0	0	0	0.006043712	0	0
RPL3L	0	0	0	0	0.005632629	0	0
ZNF213	0	0	0	0	0.003778618	0	0
CREBBP	0	0	0	0	0.043655386	0	0
MGRN1	0	0	0	0	0.006099086	0	0
EMP2	0	0	0.175697519	0	0	0	0
TMC5	0.002305326	0	0	0	0	0	0
LCMT1	0.002953999	0.06342467	0.154270992	0	0	0	0
SBK1	0	0	0	0	0	0	0.700476
EIF3C	0	0	0	0	0.002636755	0	0
FBRS	0	0	0	0	0.002636755	0	0
ZNF720	0	0	0	0	0.008006469	0	0
PHKB	0	0	0	0	0.005942901	0	0
WWP2	0	0	0	0.005273427	0	0	0
AC010547.	0.0748139	0	0	0	0	0	0
ADAT1	0.002824849	0	0.103690339	0	0	2.295684	0
SPG7	0	0	0.197659709	0.793650794	0	0	0
DEF8	0	0	0	0	0.00384987	0	0
RNASEK-C1	0	0	0	0	0.010243907	0	0
CTDNEP1	0	0.088288866	0	0	0.006489251	0	0
TMEM256	0.003746611	0.052563247	0	0	0.005621831	0	0
POLR2A	0	0	0	0	0.003786602	0	0
ATP1B2	0	0.041781042	0	0	0.002901949	0	0
TRAPPC1	0	0	0	0	0.004160184	0	0
VAMP2	0	0.039742943	0	0	0	0	0
AC093484.	0	0.052563247	0	0	0.004058129	0	0
CCDC144A	0.002227302	0	0	0	0	0	0
NATD1	0	0	0	0	0.006099086	0	0
LGALS9	0	0	0	0	0.009521271	0	0
POLDIP2	0	0	0	0	0.004897975	0	0
FLOT2	0	0	0	0	0.007735747	0	0

NSRP1	0	0.013140812	0	0	0	0	0
ATAD5	0	0.049377595	0	0	0	0	0
ZNF207	0	0	0	0	0.002706441	0	0
PSMD11	0	0	0	0	0.011777022	0	0
SLFN14	0.0748139	0	0	0	0	0	0
ZNHIT3	0	0	0	0	0.006545873	0	0
DDX52	0	0	0	0	0.003308859	0	0
RPL23	0	0	0	0	0.005192027	0	0
LASP1	0	0	0	0	0.002636755	0	0
AC003043.	0	0	0	0	0.002901949	0	0
LINC02210	0	0	0	0	0.003778618	0	0
HOXB-AS1	0	0	0	0	0.00458016	0	0
ZNF652	0.009182585	0	0	0	0	0	0
PHB	0	0	0	0	0.002706441	0	0
LUC7L3	0	0.001706242	0	0	0	0	0
SPAG9	0	0	0	0	0.009512847	0	0
C17orf67	0.007585187	0	0	0	0	0	0
VEZF1	0	0.041781042	0	0	0	0	0
DYNLL2	0	0	0	0	0.004058129	0	0
DHX40	0.017505716	0	0.073547799	0	0.003663608	0	0
METTL2A	0.101533151	0	0	0	0	0	0
BPTF	0.187034751	0.052563247	0	0	0	0	0
EXOC7	0	0	0	0	0.005591622	0	0
C17orf99	0	0	0	0	0.004589935	0	0
HGS	0.008731352	0	0	0	0.004937711	0	0
MAFG	0	0	0	0	0.003208162	0	0
FN3K	0	0	0	0	0.003505168	0	0
CEP76	0.015518167	0	0	0	0	0	0
C18orf21	0	0	0	0	0.003618403	0	0
C18orf32	0	0	0	0	0.00372409	0	0
RBCK1	0	0	0	0.064495324	0.008145876	0	0
TBC1D20	0	0.000853121	0	0	0.013395515	0	0
RBL1	0.037604871	0	0	0	0	0	0
OSER1-DT	0	0	0	0	0.011184212	0	0
TTPAL	0	0	0.137502406	0	0	0	0

SYS1	0.002227302	0	0	0	0	0	0
ELMO2	0	0.049377595	0.186032667	0	0	0	0
RIPOR3	0.07036951	0.020368258	0.291773399	0	0	0	0
NELFCD	0	0	0	0	0.004347448	0	0
AC009005.	0.003948511	0	0.180717448	0	0	0	0
MED16	0.006028262	0.050920645	0	0	0	0	0
MKNK2	0	0	0	0	0.004897975	0	0
DAPK3	0	0.033947097	0	0	0.007841659	0	0
SIRT6	0	0	0	0	0.00384987	0	0
ATG4D	0	0	0.103690339	0	0.003308859	0	0
ILF3	0.069003112	0	0	0	0.004347448	0	0
EPOR	0	0	0	0	0.004716091	0	0
GADD45GI	0	0	0	0	0.009516622	0	0
NDUFB7	0	0.038796682	0	0	0.004125072	0	0
AC123912.	0	0	0	0	0.005394325	0	0
UBA2	0	0.137264348	0	0	0	0	0
ETV2	0	0	0	0	0.002283757	0	0
LIN37	0.004026811	0	0	0	0	0	0
TBCB	0	0	0	0	0.004495897	0	0
LINC01534	0	0	0	0	0.011286936	0	0
YIF1B	0	0	0	0	0.005985909	0	0
PSMD8	0	0	0	0	0.004270894	0	0
EGLN2	0	0	0	0	0.003508981	0	0
GSK3A	0.005509551	0	0	0	0.006062406	0	0
XRCC1	0	0	0	0	0.004837017	0	0
ZNF227	0	0	0	0	0.01093344	0	0
KLC3	0.00566772	0	0	0	0.008256479	0	0
ERCC1	0	0	0	0	0.004495897	0	0
BAX	0	0	0	0	0.005287341	0	0
NOSIP	0	0	0	0	0.007055668	0	0
SCAF1	0	0	0	0	0.00426074	0	0
IRF3	0	0	0	0	0.00566229	0	0
IGLON5	0	0	0.362618958	0	0	0	0
SYT5	0	0.037033197	0	0	0	0	0
ZNF581	0	0	0	0	0.004716091	0	0

ZNF787	0	0	0	0	0.003774639	0	0
ZNF419	0	0	0	0.052328624	0	0	0
UBE2M	0	0	0	0	0.003298033	0	0
ZFY-AS1	0	0	0	0	0.006541039	0	0
ESS2	0	0	0	0	0.003607074	0	0
MAPK1	0	0	0	0	0.003010271	0	0
SMARCB1	0	0	0	0	0	0	3.76506
AP1B1	0	0	0	0	0.011460682	0	0
CCDC157	0	0	0	0	0.019235578	0	0
SEC14L4	0.001360253	0	0	0	0	0	0
CDC42EP1	0	0.049377595	0	0	0.005194954	0	0
EP300	0	0.047925313	0	0	0	0	0
SNU13	0	0	0	0	0.003136114	0	0
FAM118A	0	0	0	0	0.005273511	0	0
SIM2	0	0	0.103690339	0	0	0	0
VPS26C	0	0	0	0	0.007231129	0	0
BX322562.	0.006169549	0	0	0	0	0	0
PRMT2	0	0	0	0	0.017628218	0	0
MT-ND1	0.001646738	0.06799629	0.147095597	0	0.003505168	0	0
MT-ATP6	0	0.002586445	0	0	0	0	0

ype
